# Electromagnetic field controlled domain wall displacement for induced strain tailoring in BaTiO_3_-epoxy nanocomposite

**DOI:** 10.1038/s41598-022-11380-9

**Published:** 2022-05-07

**Authors:** Danning Li, James Barrington, Stephen James, David Ayre, Marcin Słoma, Meng-Fang Lin, Hamed Yazdani Nezhad

**Affiliations:** 1 Enhanced Composites and Structures Centre, School of Aerospace, Transport and Manufacturing, Cranfield University, Cranfield, UK; 2 Centre for Engineering Photonics, School of Aerospace, Transport and Manufacturing, Cranfield University, Cranfield, UK; 3 Faculty of Mechatronics, Warsaw University of Technology, Warsaw, Poland; 4 Department of Materials Engineering, Ming Chi University of Science and Technology, New Taipei, Taiwan; 5 Department of Mechanical Engineering and Aeronautics, Aeronautics and Aerospace Research Centre, City, University of London, London, UK

**Keywords:** Engineering, Materials science

## Abstract

Failure in an epoxy polymer composite material is prone to initiate by the coalescence of microcracks in its polymer matrix. As such, matrix toughening via addition of a second phase as rigid or/and rubber nano/micro-particles is one of the most popular approaches to improve the fracture toughness across multiple scales in a polymer composite, which dissipates fracture energy via deformation mechanisms and microcracks arrest. Few studies have focused on tailorable and variable toughening, so-called ‘active toughening’, mainly suggesting thermally induced strains which offer slow and irreversible toughening due to polymer’s poor thermal conductivity. The research presented in the current article has developed an instantaneous, reversible extrinsic strain field via remote electromagnetic radiation. Quantification of the extrinsic strain evolving in the composite with the microwave energy has been conducted using in-situ real-time fibre optic sensing. A theoretical constitutive equation correlating the exposure energy to micro-strains has been developed, with its solution validating the experimental data and describing their underlying physics. The research has utilised functionalised dielectric ferroelectric nanomaterials, barium titanate (BaTiO_3_), as a second phase dispersed in an epoxy matrix, able to introduce microscopic electro-strains to their surrounding rigid epoxy subjected to an external electric field (microwaves, herein), as result of their domain walls dipole displacements. Epoxy Araldite LY1564, a diglycidyl ether of bisphenol A associated with the curing agent Aradur 3487 were embedded with the BaTiO_3_ nanoparticles. The silane coupling agent for the nanoparticles’ surface functionalisation was 3-glycidoxypropyl trimethoxysilane (3-GPS). Hydrogen peroxide (H_2_O_2_, 30%) and acetic acid (C_2_H_4_O_2_, 99.9%) used as functionalisation aids, and the ethanol (C_2_H_6_O, 99.9%) used for BaTiO_3_ dispersion. Firstly, the crystal microstructure of the functionalised nanoparticles and the thermal and dielectric properties of the achieved epoxy composite materials have been characterised. It has been observed that the addition of the dielectric nanoparticles has a slight impact on the curing extent of the epoxy. Secondly, the surface-bonded fibre Bragg grating (FBG) sensors have been employed to investigate the real-time variation of strain and temperature in the epoxy composites exposed to microwaves at 2.45 GHz and at different exposure energy. The strains developed due to the in-situ exposure at composite, adhesive and their holding fixture material were evaluated using the FBG. The domain wall induced extrinsic strains were distinguished from the thermally induced strains, and found that the increasing exposure energy has an instantaneously increasing effect on the development of such strains. Post-exposure Raman spectra showed no residual field in the composite indicating no remnant strain field examined under microwave powers < 1000 W, thus suggesting a reversible strain introduction mechanism, i.e. the composite retaining its nominal properties post exposure. The dielectric composite development and quantifications presented in this article proposes a novel active toughening technology for high-performance composite applications in numerous sectors.

## Introduction

Among their high-performance engineering applications, fibre-reinforced polymer (FRP) composite materials have extensively been used with advantages of high specific strength and modulus, facile fabrication, considerable thermal resistance, and economic efficiency^1^. High-performance FRP composites have two major damage initiation modes when exposed to dynamic events; intra-laminar damage (e.g., matrix cracking, fibre fracture and fibre-matrix debonding) and inter-laminar damage (e.g., delamination)^2–4^. The intra-laminar damage is mainly dominated by matrix, fibre, and fibre-matrix interphase properties. However, it is challenging to tailor the properties of the fibre during composite’s fabrication process.

To overcome these property-driven drawbacks, numerous researches have been carried out for property enhancement via modifying epoxy with the inclusion of various micro- and nano-fillers as a second phase, such as rubber tougheners^5^, silica particles^6^, carbon nanoparticles^7,8^, clay^9^ and fibre coating^10^. There are also various methods developed such as inter-penetrating network^11^, polymer plasticisation^12^, the addition of rubber^13^ and/or rigid inorganic particles like glass^14^, self-healing^15^ and volume dilation^16^. It has been observed that the rubber tougheners are the most effective ones, having substantial increase in the fracture toughness^17^ of a brittle epoxy by multiple orders of magnitude, though reduction in strength and stiffness are also observed^18^. An increase in fracture toughness of the glass/epoxy composites up to 82% is achieved by incorporating both core–shell rubber particles and silica nanoparticles^19^. It is also observed by many studies that a threshold exists and above which the particles tend to agglomerate and cause a degradation in mechanical properties^20–23^. The quality of particle/matrix interfacial bonding is another crucial factor that determines the mechanical properties of a modified epoxy matrix. Thus the application of coupling agents for surface treatment of particles is introduced to achieve a better interfacial bonding between particles and matrix^24^. Despite the excellent performance of particle-toughened epoxy composites, loss in elastic modulus, tensile strength, and glass transition temperature are also reported by other studies^25^. The addition of particles into the epoxy matrix often leads to a higher viscosity, and shear thinning behaviour^26,27^. Optimal uniform particles dispersion in epoxy resin is also a critical factor that requires to be achieved for a smooth load transfer between epoxy and fillers^26^. Although the modified epoxy with particles exhibits a promising future with excellent toughening performance, microcracks still formed in FRPs when subjected to varying or extreme operating conditions or during the manufacturing^28^, indicating an inherent level of uncertainty in the material’s response that will require active toughness enhancement across the material. However, the toughness enhancement via second phase inclusion is only performed before service. When the epoxy matrix is exposed to varying mechanical loading during fabrication, storage, and service, microcracks are formed that are extremely difficult to detect and repair due to techniques limitation. Consequently, these internal micro-defects reduce the composite’s performance, and their propagation may result in disastrous structural failure. Apart from such ‘one-off’ and irreversible toughening, a few studies focused on enabling the toughening effect by introducing an internal residual compressive stress field in the epoxy resin as a result of heating, offering ‘active’ and reversible toughening^29^. Such compression mechanism mainly relies upon the discrepancy in filler/matrix thermal expansion. However, the drawback of this approach is that the control of the toughening process is extremely challenging due to the poor thermal conductivity of polymer which slows down the material’s response, mechanically. The toughening via volume dilation of embedded fillers is first proposed in a study by Ho Sung et al. It is achieved by pre-stressing the epoxy matrix via the expandable hollow microspheres under heating^30^. Thus far, there are no attempts made to date to investigate the strain behaviour of embedded fillers in rigid polymer composites under an electromagnetic field. Therefore, a study on radiation field induced strains has been investigated and presented, by incorporating ferroelectric crystals that demonstrates an electro-strain under an applied electric field.

## Research hypothesis

Dielectric nanomaterials exhibit electric field induced strain which is attributed to intrinsic mechanisms from lattice deformation and extrinsic mechanisms due to domain wall (DW) movement^31^, extensively used as actuators and transducers. The inclusion of such material within a rigid epoxy FRP materials can impose a compressive stress field in its surrounding epoxy matrix when activated its DW movements by external electric field stimulation. As result of the DW movements, a microwave stimulation at GHz frequencies induces effective dipolar displacement (leading to intrinsic strains) to the nanomaterial’s molecules that, at the interface with their surrounding rigid polymer, is converted to compressive mechanical strain. The hypothesis of this research was based upon suggesting that microcrack propagation during dynamic and impact events would be suppressed under such microwave induce d compressive stress–strain field, i.e. higher strain energy would be required to create new fracture surfaces, however the current article presents attempts on the quantification of the field induced strains. Therefore, the study focused on: 1—development of a modified epoxy with uniformly distributed dielectric nanomaterials that exhibit electro-strain, 2—in-situ quantification of its strain response under an electromagnetic field (e.g. microwave), and 3—establishment of a theoretical constitutive equation underpinning the correlation between the induced mechanical field and the microwave field.

To investigate the microstructural response of such dielectric nanomaterial embedded nanocomposite under an electromagnetic field, firstly, we developed a modified epoxy system with uniformly distributed barium titanate (BaTiO_3_) nanoparticles. Materials’ properties, functionalisation and fabrication methods developed in the research are described in "Materials and fabrication" section. Constitutive equations underpinning the multi-physics field induced strain and temperature have been provided in "Theoretical multi‑physics constitutive equations" section. With a second phase added to the epoxy system, the dielectric properties have been improved. Meanwhile, it also has effects on the curing extent of the epoxy system. Basic dielectric and cure state characterisation methods and their results are presented and described in "General material characterisations" section and "General material characterisations" section, respectively. To experimentally measure the perceivable strain effect induced by the stimulation of embedded BaTiO_3_ particles’ strains under microwave stimulation, fibre optic sensors utilising fibre Bragg grating (FBG) technique have been employed. The results of the in-situ measurements have been presented in In‑situ strain response under microwave at different power levels" section. Raman spectroscopy was employed to observe residual internal stresses post microwave exposure. Its results are presented and discussed in "Raman characterisation" section. The theoretical solution from the newly established constitutive expressions have been presented, where their ability to underpin the experimental data have been assessed.

## Electric field induced strain in ferroelectric materials

BaTiO_3_ like other ferroelectric materials exhibit spontaneous polarisation at its polar phase that could be switched by applying an external electric field^32,33^, containing crystals having a net permanent polarisation which is the vector sum of all the dipole moments in a unit cell^34,35^, due to the absence of a centre of symmetry. When an electric field is applied, while the strength of the field increases, the polarisation increases and reaches a saturation state where all dipoles are aligned with the field direction. When the external field is removed, the polarisation may not return back to zero, i.e. displaying a remnant polarisation $P_{r}$^36^.

**Figure 1:**
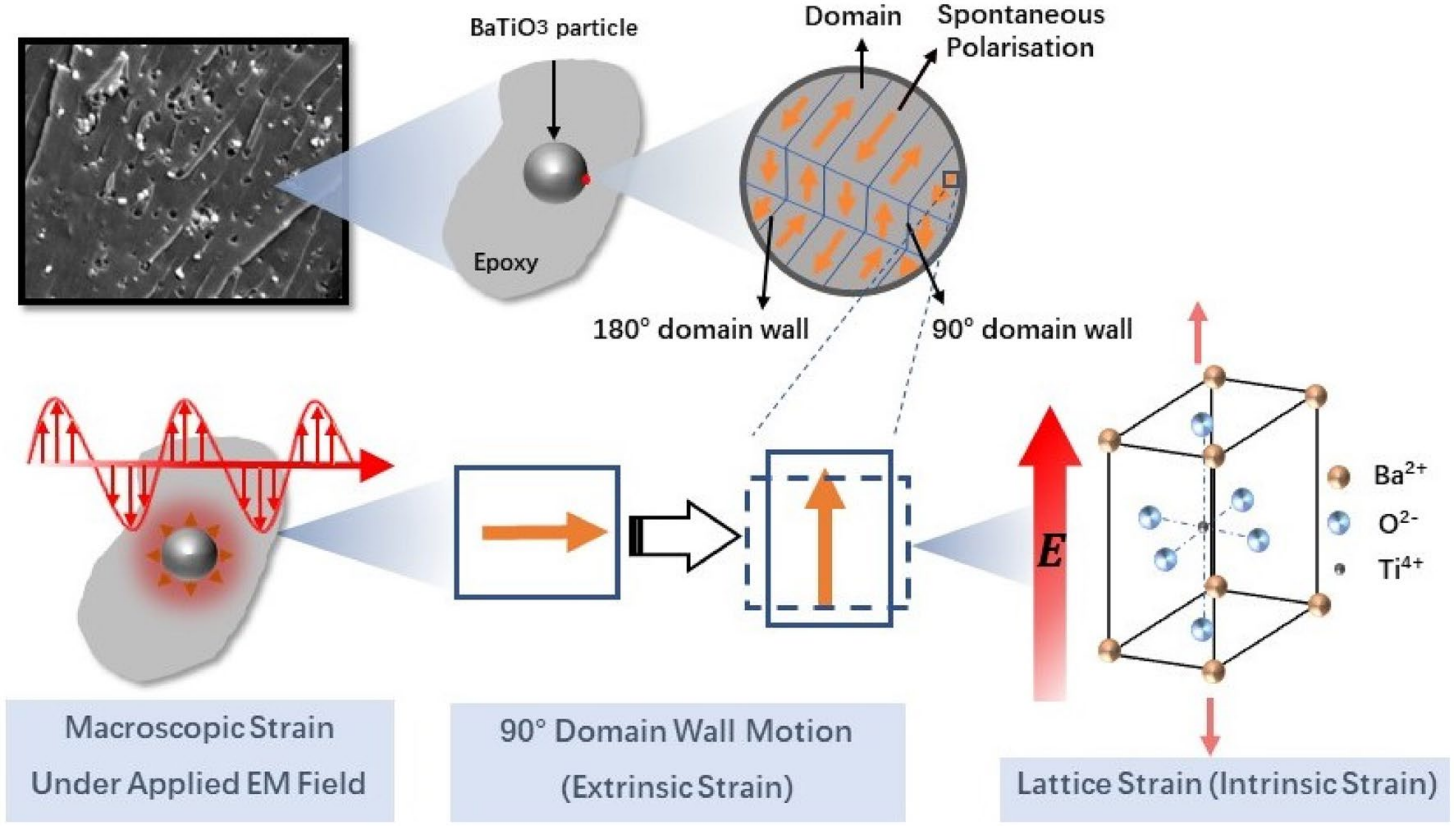
Schematic diagram of the extrinsic strain and intrinsic strain that contributes to macroscopic strain in a tetragonal BaTiO_3_ crystal embedded in epoxy (red, green and blue arrows represent electric field induced intrinsic strain, extrinsic strain and macroscopic strain, respectively).

BaTiO_3_ domain wall is characterised by the angle of polarisation axes between two adjacent domains, such as 180° and 90° domain walls^37^, existing in a tetragonal phase of BaTiO_3_^38^. Generally speaking, 180° domain walls are only responsive under electric fields while the other non-180° domain walls are responsive under both electric and mechanical stress fields^37^. Therefore, the DW configuration of BaTiO_3_ is defined by its own structure, and it changes by dipole displacements under an electric field or mechanical loading^39^. When an electric field is applied to the BaTiO_3_ crystals with a perovskite structure, three main representative deformations contribute to its mechanical strain response^40^, the intrinsic strain is induced by its electrostriction and piezoelectric effects while the extrinsic strain is attributed to the domain wall movement in the polydomain materials as presented in Fig. 1.

The 90° domain switching in BaTiO_3_ introduces a large electro-strain due to the exchange of two different crystallographic axes, and the field induced strain is one or two orders of magnitude larger than the linear electro-strain of piezoelectric materials. After the removal of the electric field, the domain structure cannot be restored to its original form due to lack of energy and remnant polarisation in each domain. Therefore, the 90° domain switching is theoretically a one-time process^41^. Due to the lack of driving force to restore the original domain configuration after removing the field, the domain wall movement is irreversible and consequently the macroscopic strain^42^. The 90° domains in BaTiO_3_ crystals are capable of introducing a large strain that is attributed to the crystallographic axes exchange under alternating electric field^42^. However, it is difficult to distinguish the contributions from intrinsic and extrinsic (domain wall movement) contributions and conventionally, the single-domain crystals are considered to be only affected by the intrinsic properties while the contributions of the extrinsic domain wall movement are investigated in the polydomain crystals, as observed in^37^. Therefore, in this research, polydomain BaTiO_3_ nanoparticles is employed to achieve the field-induced strain effect.

## Interaction of ferroelectric materials with the microwave field

The mechanism of the material and microwave interaction is particularly complex and it involves the orientation of dipoles, free-to-move electrons, domain wall movement and electron spins, which are activated by the electric and/or magnetic field components^43^. The essence of the interaction between the dielectric material and the electromagnetic field is energy transformation at the molecular level. Therefore, the dielectric properties of a material, which is measured as the complex permittivity quantifies the ability of a dielectric material to absorb and store electrical potential energy when exposed to a microwave field^44^. The complex permittivity that represents the microwave-material interactions are expressed by:

1 $$\varepsilon = \varepsilon^{\prime} - j\varepsilon ^{\prime\prime}$$

where $\varepsilon^{\prime}$ is the real parts of the permittivity that indicating the ability to store microwave energy of the material while $\varepsilon ^{\prime\prime}$ is the imaginary part of the permittivity that indicating the ability to dissipate the microwave energy^44^. The dielectric loss tangent is expressed by:

2 $$\tan \delta = \frac{\varepsilon ^{\prime\prime}}{{\varepsilon^{\prime}}}$$

The loss tangent is the measure of the material to convert absorbed energy into other forms of energy such as heat^45^. The dipoles in an alternating electric field will reorient themselves to align with the new field direction. However, this process cannot occur instantaneously due to inherent non-zero inertia, and thus some time is required to fulfil the reorientation of the dipoles. At microwave frequencies, dipole polarisation is assumed to be the most dominant one for energy transfer at the molecular level.

## Theoretical multi-physics constitutive equations

In this section, the theoretical analysis of the strain field induced in BaTiO_3_ nanoparticles embedded epoxy under microwave exposure is established. Firstly, the theory of dynamic elastic response of tetragonal BaTiO_3_ nanoparticles under alternating electric field at the microwave exposure is presented and discussed^46^. The displacement of the DWs is utilised as the eigenstrain for the estimation of the strain field in the BaTiO_3_-epoxy nanocomposite. A novel approach based on the principle of virtual work of the equivalent eigenstrain is incorporated in the calculation of the inhomogeneous inclusion^47^. Based on this approach, a theoretical framework of microwave activated dynamic elastic response of the nanocomposite has been established. Finally, the non-uniform distribution of the microwave field and the interaction with the samples have been incorporated in the framework.

### Deformation in a representative crystallite of BaTiO_3_ under the microwave field

**Figure 2:**
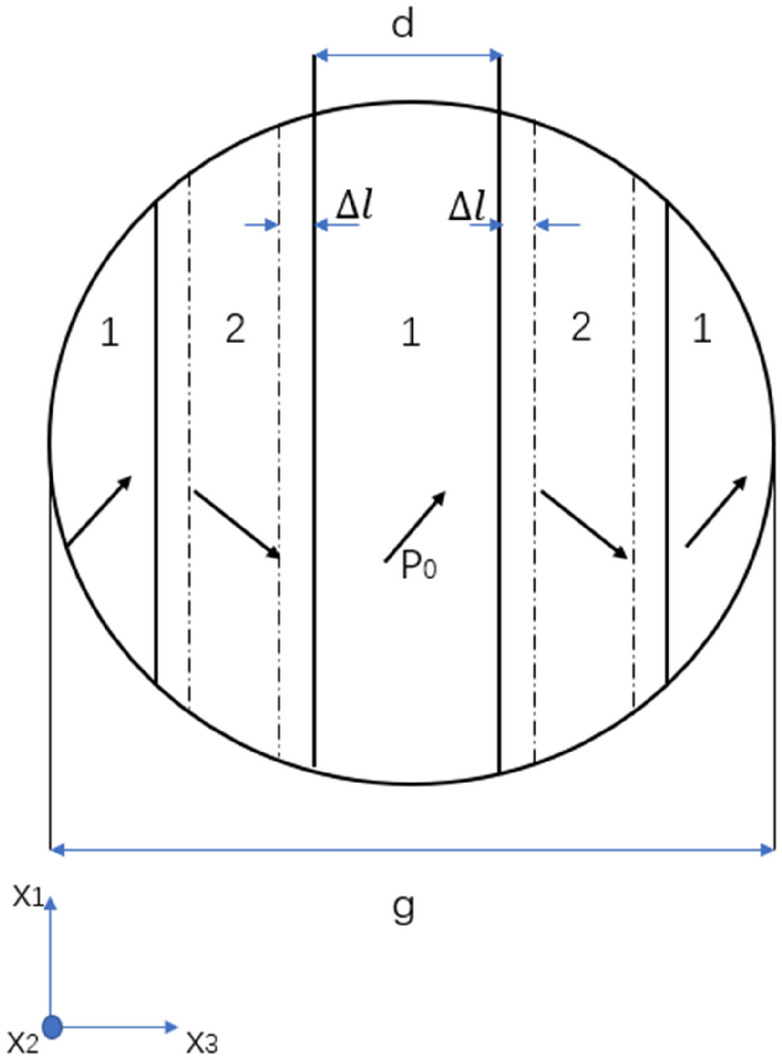
Schematic illustration of a spherical nanoparticle (simplified as a crystallite) with a laminar 90° domain structure with spontaneous polarisation ${{\varvec{P}}}_{0}$ and domain size ${\varvec{d}}$, recreated from^48^.

The 90° DW movement is considered to have the major contribution to the extrinsic field-induced strain. Therefore, the tetragonal BaTiO_3_ nanoparticle is assumed to be formed by several multi-domain crystallites that contain a laminar 90° domain structure. A conceptual schematic of a crystallite is shown in Fig. 2. $\Delta l$ is the displacement of domain walls under microwave radiation; $d$ is domain size and $g$ (identified in the figure) is the particulate size, i.e. in our case and grain size in polycrystal. The domain wall movement in such crystallite is assumed to be not affected by that in other crystallites.

Before the application of the microwave field, it is assumed that the DWs are in an equilibrium state, and all the domains are of an identical size, dielectric properties and elastic properties. Therefore, the interactions between adjacent domains are not included as it is assumed negligible compared with other factors as suggested in a previous study^49^. Moreover, the internal electric and mechanical fields are assumed to be negligible, hence no forces acting on the DWs prior to the microwave exposure^50^. When the microwave field is applied, the interactions with the magnetic component are negligible since BaTiO_3_ is non-ferrite. The alternating electric component of the microwave field is assumed to be homogenous across the whole nanoparticle in this research. It is assumed that the spontaneous polarisations in the 90° domains re-orient themselves with the direction of an applied alternating electric field. Consequently, a driving force $f_{A} \left( t \right)$ as the function of time is introduced on the DWs that enables the DW motion due to polarisation change^51^. The displacement of the DW movement is denoted as $\Delta l$ in Fig. 2. Due to the BaTiO_3_ ferroelectricity, such movement introduces an internal electric field $E$ in the nanoparticle due to the change in the polarisation direction. As a result, the internal stress field is also no longer negligible due to the newly developed internal electric field and DW movement from its equilibrium position. An additional force $f_{I}$ on the DWs is developed subsequently due to the change in the internal electric and stress fields from zero state. Due to the displacements in DWs, the electric dipole moment $\Delta p$ induced by the internal electric field, and the elastic dipole moments $\Delta v$ induced by the internal mechanical stress field are given by^52^:

3 $$\Delta p = \sqrt 2 P_{0} \Delta l$$

4 $$\Delta v = 2S_{0} \Delta l$$

where $P_{0}$ is the spontaneous polarisation, and $S_{0}$ is the spontaneous deformation. The correlation between $\Delta l$ and the dipole moments is assumed to be linear, and their internal energy $W_{electric}$ under the internal electric field $E$, and $W_{elastic}$ under mechanical stress field $T_{13}$ are:

5 $$W_{electric} = - \sqrt 2 P_{0} \Delta lE$$

6 $$W_{elastic} = - 2S_{0} \Delta lT_{13}$$

where $E$ is the internal electric field, and $T_{13}$ is the internal mechanical stress $T_{ij}$ ($i$, $j$ = 1, 2, 3) as illustrated in a cartesian coordinate system ($x_{1}$, $x_{2}$, $x_{3}$) in Fig. 2.

The expression for the DW motion is^51^:

7 $$kA_{DW} \Delta l = - \left( {\frac{{\partial W_{electric} }}{\partial \Delta l} + \frac{{\partial W_{elastic} }}{\partial \Delta l}} \right)$$

By the derivation of Eqs. (5) and (6), the force $f_{I}$ due to the internal fields per unit area $k\Delta l$ is obtained from

8 $$k\Delta l = \sqrt 2 P_{0} E + 2S_{0} T_{13}$$

where $k$ is the force constant per unit DW area, and $A_{DW}$ is the area of the DW. The displacements for the DWs from their equilibrium state under the microwave field are assumed to have the same magnitude, $\left| {\Delta l\left( t \right)} \right|$. The force $f_{I}$ per unit DW area at a random point $r$, and time $t$ can then be expressed by Eq. (8):

9 $$\begin{array}{*{20}c} {f_{I} \left( {r,t} \right) = \sqrt 2 P_{0} {\text{E}}_{1} ,\left( {r,t} \right) + 2S_{0} T_{13} \left( {r,t} \right) } \\ \end{array}$$

The DWs motion is then described by the Newton’s second law of motion:

10 $$m\Delta \ddot{l} = \overline{f}_{I} \left( t \right) + f_{A} \left( t \right)$$

where $m$ is the mass of a DW per unit area, $\overline{f}_{I} \left( t \right)$ is the mean value of the DWs driven force $f_{I}$ from the internal fields, $f_{A} \left( t \right)$ is the aforementioned exerted force on the DWs by the applied microwave field. The $\overline{f}_{I} \left( t \right)$ is then calculated by averaging the $f_{I} \left( {r,t} \right)$ over the summation of the DWs surfaces ($d\Sigma$) in a nanoparticle, as presented below^46^.

11 $$\overline{f}_{I} \left( t \right) = \frac{1}{ \Sigma }(2S_{0} \mathop \smallint \limits_{\Sigma } T_{13} \left( {r, t} \right)d\Sigma + \sqrt 2 P_{0} \mathop \smallint \limits_{\Sigma } E\left( {r,t} \right)d\Sigma )$$

The DW displacements induced internal electric field $E\left( {r,t} \right)$ and internal mechanical field $T_{13} \left( {r, t} \right)$ in Eq. (11) must firstly be calculated prior to the $\overline{f}_{I} \left( t \right)$ calculation. The internal electric field was evaluated in a quasi-static approximation for a given microwave field, and $E\left( {r,t} \right)$ is considered to be proportional to the DW displacement $\Delta l \left( t \right)$. The interal mechanical stress field $T_{ij}$ was considered as a dynamic problem as the DW motions lag behind the 2.45 GHz microwave frequency^46^. In this study, only the extrinsic effect, i.e. the DW motions, is considered as they are commonly accepted to be the primary contributor to the electro-strain compared with the intrinsic effect such as piezoelectric effect and electrostriction.

To obtain the value of the internal mechanical stress field, the change of the spontaneous deformation $S_{0}$ introduced by the DW displacements in Eq. (11) was calculated, thus the $\Delta S_{13}^{0} \left( {r,t} \right)$ and $\Delta S_{31}^{0} \left( {r,t} \right)$ were approximated by^46^:

12 $$\Delta S_{13}^{0} \left( {r,t} \right) = \Delta S_{31}^{0} \left( {r,t} \right) = S_{0} \frac{\Delta l \left( t \right)}{w}\delta \left( {V_{DW} } \right)$$

where $w$ is the thickness of a 90° DW, ${V}_{DW}$ is the volume of DWs without an applied microwave field, and $\delta \left({V}_{DW}\right)$ is the Dirac delta function for ${V}_{DW}$.

The thickness $w$ is approx. 100 Å, much smaller compared with the domain width $d$ and the particle size^48^, therefore, Eq. (12) was simplified to Eq. (13)^46^.

13 $$\begin{array}{*{20}c} {\Delta S_{13}^{0} \left( {r,t} \right) = \Delta S_{31}^{0} \left( {r,t} \right) = S_{0} \Delta l \left( t \right)\delta \left( \Sigma \right)} \\ \end{array}$$

where $\Sigma$ represents the midplanes of DWs when $\Delta l=0$, ${\delta }_{3}\left(\Sigma \right)$ is the Dirac delta function of the $\Sigma$ with a unit normal directed along the ${x}_{3}$ axis^53^.

The electric component of the oscillating microwave field is a sinusoidal function of time and the DW displacement $\Delta l\left(t\right)$ is written as

14 $$\Delta l\left( t \right) = \Delta l_{m} \sin \omega t$$

where $\Delta {l}_{m}$ is the maximum displacement of the DWs under the microwave field. The nanoparticle is assumed to be a homogeneous and isotropic dielectric medium, and the internal electric field $E\left(r,t\right)$ is proportional to $\Delta l\left(t\right)$,

15 $${\varvec{E}}\left({\varvec{r}},{\varvec{t}}\right)=-\frac{{{\varvec{P}}}_{0}}{{{\varvec{\varepsilon}}}_{0}}\frac{\Delta {{\varvec{l}}}_{{\varvec{m}}}}{{\varvec{d}}}{{\varvec{h}}}_{{\varvec{e}}}\left({\varvec{r}}\right)\mathbf{sin}{\varvec{\omega}}{\varvec{t}}$$

where ${\varepsilon }_{0}$ is the permittivity of the free space (${\varepsilon }_{0}=8.8542\times {10}^{-12}$ F/m), $d$ is the width of the DW, and ${h}_{e}\left(r\right)$ are approximated as^46^:

16 $${{\varvec{h}}}_{{\varvec{e}}}\left({\varvec{r}}\right)=\frac{\sqrt{2}}{{{\varvec{\epsilon}}}_{11}+2{{\varvec{\varepsilon}}}^{\boldsymbol{*}}}$$

where ${\epsilon }_{11}$ is first component of the dielectric permittivity tensor ${\epsilon }_{ij}$ ($i,j=\mathrm{1,2},3$) that represents the dielectric permittivity of the crystallite as illustrated in Fig. 2, and ${\varepsilon }^{*}$ is the dielectric permittivity of the nanoparticle. The force ${\overline{f} }_{I}$ was then calculated with the internal mechanical stress field ${T}_{13}\left(r, t\right)$, and the internal electric field $E\left(r,t\right)$ from Eqs. (15) and (16). The field-induced force ${f}_{A}$ is due to the effective electric field in the ${x}_{1}$ direction of the applied microwave field as illustrated in Fig. 2. The electric field component ${E}_{A1}$ is a function of time as follows:

17 $${{\varvec{E}}}_{{\varvec{A}}1}\left({\varvec{t}}\right)={{\varvec{E}}}_{{\varvec{m}}}\mathbf{sin}({\varvec{\omega}}{\varvec{t}}+\mathbf{\varnothing })$$

where ${E}_{m}$ is the amplitude of the electric component of the microwave field, and $\mathrm{\varnothing }$ is the phase lag of the DW motion relative to the oscillatory microwave field. Equation (17) is then substituted into Eq. (8) with the second part equalling zero as ${f}_{A}\left(t\right)$ is only introduced by an electric field:

18 $${{\varvec{f}}}_{{\varvec{A}}}\left({\varvec{t}}\right)=\sqrt{2}{{\varvec{P}}}_{0}{{\varvec{E}}}_{{\varvec{m}}}\mathbf{sin}({\varvec{\omega}}{\varvec{t}}+\mathbf{\varnothing })$$

Finally Eqs. (11), (15), and (18) are substituted into Eq. (10), giving:

19 $$\begin{gathered} \frac{{\Delta l_{m} }}{d}\left[ {\left( {2GS_{0}^{2} { }\overline{h}_{s} \left( \omega \right) + \sqrt 2 \left( {\frac{{P_{0}^{2} }}{{{ }\varepsilon_{0} }}} \right)\overline{h}_{e} - md\omega^{2} } \right)\sin \omega t + 2GS_{0}^{2} { }\overline{h}_{c} \left( \omega \right)\cos \omega t} \right] \hfill \\ \quad \quad = \sqrt 2 P_{0} E_{m} \left( {cos\emptyset \sin \omega t + sin\emptyset {\text{cos}}\omega t} \right) \hfill \\ \end{gathered}$$

where ${\overline{h} }_{s} ,{\overline{h} }_{c},\mathrm\,{and}\,{\overline{h} }_{e}$ are the average of ${h}_{s}\left(r\right),{h}_{c}\left(r\right) and\, {h}_{e}\left(r\right)$ over DW surface $\Sigma$, evaluated using the expressions outlined in^46^. From Eq. (19), it could be seen that the DW displacement $\Delta {l}_{m}$ is correlated with the properties and strength of the electric component of the microwave field ${E}_{m}$ and the dielectric permittivity of the nanoparticle. The value of the DW displacement $\Delta {l}_{m} \left(\omega \right)$ could be obtained by numerical computations of the ${\overline{h} }_{s}\left(\omega \right)$ and ${\overline{h} }_{c}\left(\omega \right)$.

To get an approximate estimate, a further simplification was made that the nanoparticles are sufficiently small to be considered as a multi-domain single crystal, namely a crystallite. Therefore, the crystallite as illustrated in Fig. 2 could be considered as a single BaTiO_3_ nanoparticle without any adjacent crystallite, and hence no corresponding internal electric and stress field. Consequently, Eq. (10) is simplified to:

20 $$m\Delta \ddot{l} = f_{A} \left( t \right) = \sqrt 2 P_{0} E_{m} \sin (\omega t + \emptyset )$$

It is assumed that the DW movement under the oscillatory microwave field is a simple harmonic motion with a driven force of ${f}_{A}\left(t\right)$. Neglecting the lag in the dipoles reorientation, the DW motion becomes in phase with the oscillating field. The driving force at the maximum electric field strength ${f}_{m}$ is then obtained by:

21 $$f_{m} = \sqrt 2 P_{0} E_{m} ,$$

and the amplitude $\Delta {l}_{m}$:

22 $$\Delta l_{m} = \frac{\sigma L}{Y} = \frac{{f_{m} L}}{AY} = \frac{{f_{m} L}}{{d^{2} Y}}$$

where $Y$ is the young’s modulus of the BaTiO_3_ nanoparticle and $d$ is the domain width where the area of the domain is approximated to be ${d}^{2}$.

### Epoxy embedded with a BaTiO_3_ nanoparticle

Eshelby’s inclusion approach has been employed in this study to compute the effective stress field within an epoxy matrix with the embedded BaTiO_3_ nanoparticle. General Eshelby’s inclusion problem involves ellipsoidal inclusions in an infinite linear elastic medium^54,55^. Firstly, equations based on the elastic theory to solve the eigenstrain of Elshelby’s inclusion have been utilised, in which the total strain of infinitesimal deformations is expressed as:

23 $$\varepsilon_{ij} = e_{ij} + \varepsilon_{ij}^{*}$$

where ${e}_{ij}$ is the elastic strain, and $\varepsilon_{ij}^{*}$ is the eigenstrain. The compatibility condition of total strain $\varepsilon_{ij}$ is expressed in terms of displacement ${u}_{i}$, i.e.

24 $$\varepsilon_{ij} = \frac{{u_{i,j} + u_{j,i} }}{2}.$$

The stress ${\sigma }_{ij}$ is then expressed by the Hooke’s Law,

25 $${\sigma }_{ij}={C}_{ijkl}{e}_{kl}={C}_{ijkl}\left({\varepsilon }_{kl}-{\varepsilon }_{kl}^{*}\right)={C}_{ijkl}\left({u}_{k,l}-{\varepsilon }_{kl}^{*}\right),$$

where ${C}_{ijkl}$ is the effective stiffness tensor, ${C}_{ijkl}={C}_{ijlk}$ and ${C}_{ijkl}{u}_{k,l}={C}_{ijkl}{u}_{l,k}$. In the region where ${\varepsilon }_{kl}^{*}=0$, Eq. (25) is re-arranged to:

26 $${\sigma }_{ij}={C}_{ijkl}{\varepsilon }_{kl}={C}_{ijkl}{u}_{k,l}$$

**Figure 3:**
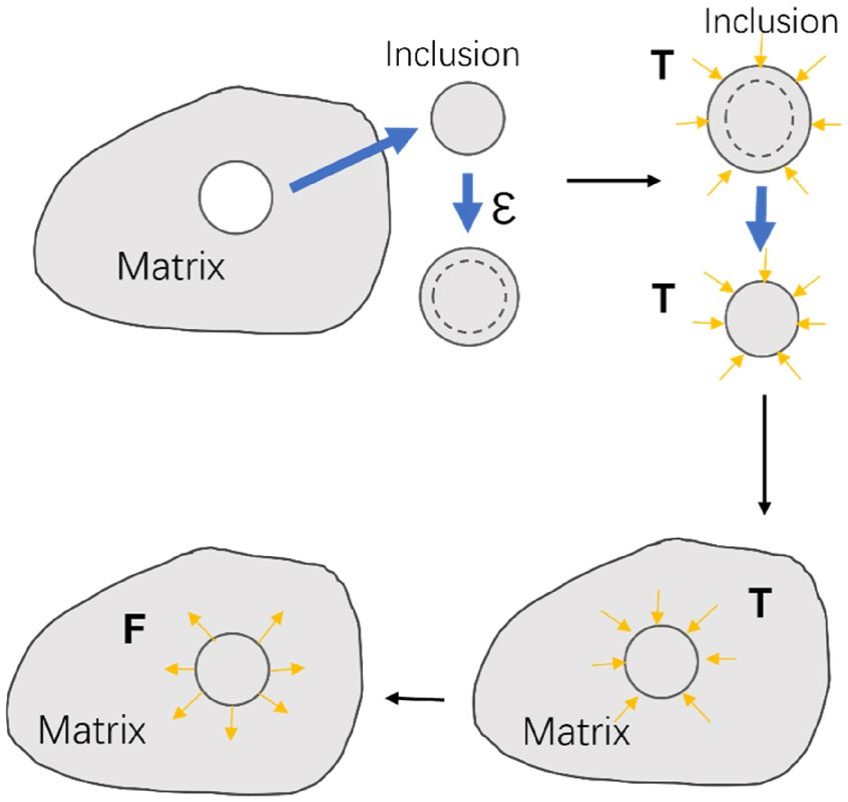
Schematic diagram of Eshelby's inclusion utilised in our analysis.

The inversed Eq. (25) is then expressed by:

27 $${\varepsilon }_{ij}-{\varepsilon }_{ij}^{*}={C}_{ijkl}^{-1}{\sigma }_{kl}={S}_{ijkl}{\sigma }_{kl},$$

where ${S}_{ijkl}$ is the compliance tensor that has the same symmetries as the stiffness tensor. The equation of equilibrium is then expressed as follows:

28 $${\sigma }_{ij,j}+{f}_{i}=0,\left(i=\mathrm{1,2},3\right),$$

where ${f}_{i}$ is the body force, and the boundary condition is:

29 $${T}_{i}={\sigma }_{ij}{n}_{j},$$

where ${n}_{j}$ is the outward unit normal vector to the boundary. The corresponding stress and strain field of a homogeneous inclusion in an infinite medium could be obtained using Green’s function method with the profile of the inclusion^56^: The Eshelby inclusion’s problem is applied as illustrated in Fig. 3. It is assumed that the inclusion has a dynamic deformation of the eigenstrain $\varepsilon (t)$ as a function of time under the microwave field. A fictitious load $T$ is applied to the inclusion to maintain the original shape. The inclusion is then placed back into the hole within the infinite epoxy matrix with the original shape and size. After removing the applied $T$, the inclusion exerts a traction force $F$ on the matrix, which equals $-T$.

It is assumed that the inclusion is in ellipsoids shape and have identical elastic properties with the matrix. Furthermore, the eigenstrain in the inclusion is assumed uniform. Therefore, the inhomogeneous inclusion of embedded BaTiO_3_ nanoparticles has been solved by transforming the inhomogeneous inclusion into a homogenous inclusion. The nanoparticle with residual strain embedded within the epoxy was transformed into a homogenous inclusion problem with a distribution of equivalent eigenstrain.

**Figure 4:**
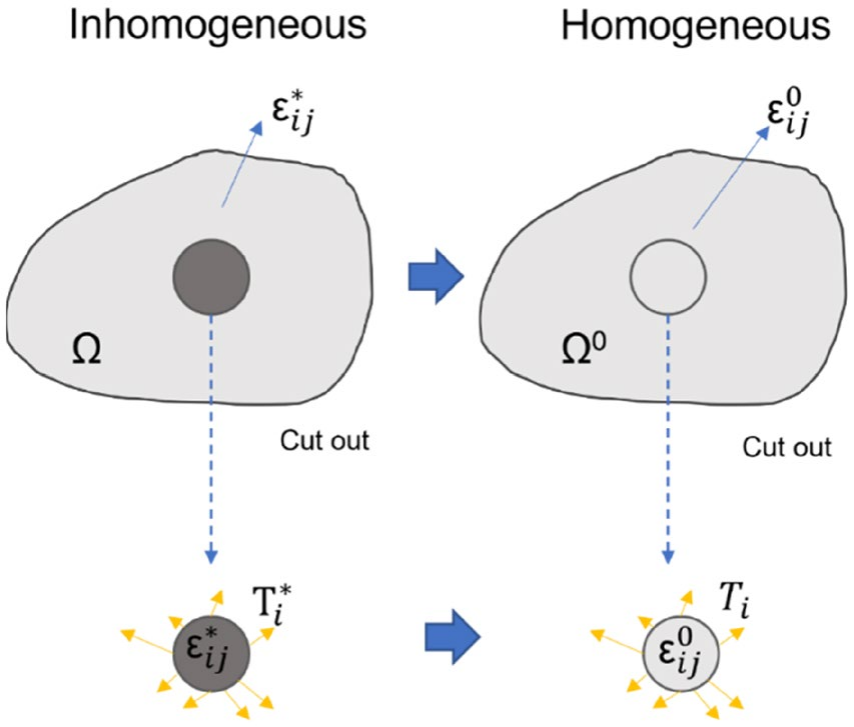
Schematic diagram of the principle of equivalent eigenstrain of transforming inhomogeneous problem to a virtual homogeneous problem.

The transformation illustrated in Fig. 4 was based on the principle of virtual work^47^. Firstly, the inclusions in both inhomogeneous and homogeneous cases in Fig. 4 are imaginarily cut-sectioned from the matrix, and the virtual works are expressed in Eq. (30), over the Ω region of the inhomogeneous problem, and Eq. (31) over the Ω^0^ region of the virtual homogeneous problem.

30 $$\mathop \smallint \limits_{v} f_{i}^{*} \delta u_{i} dv + \mathop \smallint \limits_{s} T_{i}^{*} \delta u_{i} ds = \mathop \smallint \limits_{v} \sigma_{ij}^{*} \delta {\upvarepsilon }_{ij} dv$$

31 $$\mathop \smallint \limits_{v} f_{i} \delta u_{i} dv + \mathop \smallint \limits_{s} T_{i} \delta u_{i} ds = \mathop \smallint \limits_{v} \sigma_{ij} \delta {\upvarepsilon }_{ij} dv,$$

where $\delta {u}_{i}$ is the virtual displacement, $\delta \varepsilon_{ij}$ is the virtual strain, ${f}_{i}$ is the body force, ${T}_{i}$ is the traction force on the boundary, and $\sigma_{ij}$ is the stress field in the nanoparticle. Equation (31) minus Eq. (30) gives:

32 $$\mathop \smallint \limits_{v} (f_{i} - f_{i}^{*} )\delta u_{i} dv + \mathop \smallint \limits_{s} \left( {T_{i} - T_{i}^{*} } \right)\delta u_{i} ds = \mathop \smallint \limits_{v} (\sigma_{ij} - \sigma_{ij}^{*} )\delta {\upvarepsilon }_{ij} dv.$$

As the stress state in both conditions are identical, therefore, ${{f}_{i}=f}_{i}^{*}$, and ${T}_{i}={T}_{i}^{*}$. The $\delta \varepsilon_{ij}$ can take any arbitrary value, and Eq. (32) is then expressed as follows:

33 $${{\sigma }_{ij}=\sigma }_{ij}^{*}$$

From the stress expression in Eq. (25), the stress state is obtained as Eq. (34) over Ω, and Eq. (35) over Ω^0^:

34 $$\sigma_{ij}^{*} = C_{ijkl}^{*} \left( {\varepsilon_{kl} - \varepsilon_{kl}^{*} } \right)$$

35 $$\sigma_{ij} = C_{ijkl} \left( {\varepsilon_{kl} - \varepsilon_{kl}^{0} } \right)$$

where ${C}_{ijkl}$ and $C_{ijkl}^{*}$ are the elastic constant tensors of the homogeneous and inhomogeneous inclusion, respectively, ${\varepsilon }_{kl}$ is the total strain, ${\varepsilon }_{kl}^{*}$ is the real residual strain in the inhomogeneous Ω, and ${\varepsilon }_{kl}^{0}$ is the equivalent eigenstrain in the homogeneous Ω^0^. Since ${{f}_{i}=f}_{i}^{*}$ and ${T}_{i}={T}_{i}^{*}$, the deformation of the nanoparticle in epoxy in Ω is equivalent to that of the virtual homogeneous inclusion in Ω^0^ by incorporating an equivalent eigenstrain ${\varepsilon }_{kl}^{0}$. The substitution of Eq. (33) with Eqs. (34) and (35) gives:

36 $$C_{ijkl}^{*} \left( {\varepsilon_{kl} - \varepsilon_{kl}^{*} } \right) = C_{ijkl} \left( {\varepsilon_{kl} - \varepsilon_{kl}^{0} } \right)$$

The equivalent eigenstrain in Ω^0^ is then expressed by:

37 $${\varepsilon }_{kl}^{0}=\left(1-{C}_{ijkl}^{*}{S}_{ijkl}\right){\varepsilon }_{kl}+{C}_{ijkl}^{*}{S}_{ijkl}{\varepsilon }_{kl}^{*}$$

Therefore, with the equivalent eigenstrain ${\varepsilon }_{ij}^{0}$, the stress and strain field in the nanoparticle over Ω could be calculated using Green’s function method as follows^47,56^, accounting for the displacement field for a 3D inclusion:

38 $$u_{i} \left( {\varvec{X}} \right) - \mathop \smallint \limits_{ - \infty }^{\infty } C_{jlmn} \varepsilon_{mn}^{*} \left( {\user2{X^{\prime}}} \right)G_{ij,l} \left( {{\varvec{X}} - \user2{X^{\prime}}} \right)d\user2{X^{\prime}},\user2{X,X} \in {\Omega }$$

where ${G}_{ij,l}\left({\varvec{X}}-{{\varvec{X}}}^{\boldsymbol{^{\prime}}}\right)$ is the Green’s function, and

39 $${G}_{ij,l}\left({\varvec{X}}-{{\varvec{X}}}^{\boldsymbol{^{\prime}}}\right)=\frac{\partial }{\partial {x}_{l}}{G}_{ij}\left({\varvec{X}}-{{\varvec{X}}}^{\boldsymbol{^{\prime}}}\right)-\frac{\partial }{\partial {{x}_{l}}^{^{\prime}}}{G}_{ij}\left({\varvec{X}}-{{\varvec{X}}}^{\boldsymbol{^{\prime}}}\right)$$

The strain field is then obtained from Eq. (38), i.e.

40 $${\varepsilon }_{ij}\left({\varvec{X}}\right)=-\frac{1}{2}{\int }_{-\infty }^{\infty }{C}_{klmn}{\varepsilon }_{mn}^{*}\left({{\varvec{X}}}^{\boldsymbol{^{\prime}}}\right)\left\{{G}_{ik,lj}\left({\varvec{X}}-{{\varvec{X}}}^{\boldsymbol{^{\prime}}}\right)+{G}_{jk,li}\left({\varvec{X}}-{{\varvec{X}}}^{\boldsymbol{^{\prime}}}\right)\right\}d{{\varvec{X}}}^{\boldsymbol{^{\prime}}}$$

The total strain in Eq. (37) is then expressed equivalent to the eigenstrain ${\varepsilon }_{ij}^{0}$ given by Eq. (40), which gives:

41 $${{\varvec{\varepsilon}}}_{{\varvec{p}}{\varvec{q}}}\left({\varvec{X}}\right)=-\frac{1}{2}{\int }_{-\boldsymbol{\infty }}^{\boldsymbol{\infty }}{{\varvec{C}}}_{{\varvec{k}}{\varvec{l}}{\varvec{i}}{\varvec{j}}}{{\varvec{\varepsilon}}}_{{\varvec{i}}{\varvec{j}}}^{0}\left({{\varvec{X}}}^{\boldsymbol{^{\prime}}}\right)\left\{{{\varvec{G}}}_{{\varvec{p}}{\varvec{k}},{\varvec{l}}{\varvec{q}}}\left({\varvec{X}}-{{\varvec{X}}}^{\boldsymbol{^{\prime}}}\right)+{{\varvec{G}}}_{{\varvec{q}}{\varvec{k}},{\varvec{l}}{\varvec{p}}}\left({\varvec{X}}-{{\varvec{X}}}^{\boldsymbol{^{\prime}}}\right)\right\}{\varvec{d}}{{\varvec{X}}}^{\boldsymbol{^{\prime}}}$$

**Figure 5:**
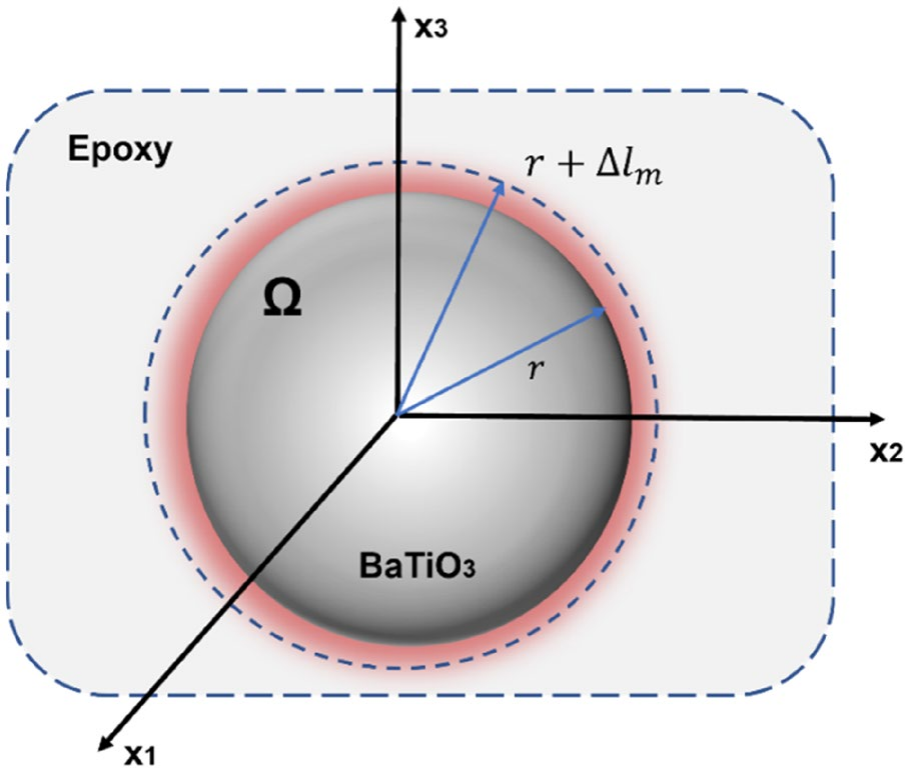
Schematic view of a BaTiO_3_ nanoparticle with radius (${\varvec{r}}$) embedded in the epoxy matrix with eigenstrain ($\Delta {{\varvec{l}}}_{{\varvec{m}}}$).

Therefore, the corresponding deformation field in the inclusion and the matrix can be calculated using Green’s function method with the equivalent eigenstrain ${\varepsilon }_{ij}^{0}$. As illustrated in Fig. 5, a BaTiO_3_ nanoparticle is embedded in the epoxy matrix, assumed to be in full bonding with the epoxy (i.e. no disbond or interfacial defect) and the epoxy is assumed to be infinite. The BaTiO_3_ nanoparticle and the epoxy are assumed to be isotropic. A nominal eigenstrain ${\varepsilon }_{ij}^{*}$, due to the DW movement is prescribed as shown in the figure. It is assumed that the eigenstrain is the collective displacements of all the domains that may exhibit different polarisation in all possible directions.

The nominal eigenstrain is denoted as ${\varepsilon }_{ij}^{*}$:

42 $${{\varvec{\varepsilon}}}_{{\varvec{i}}{\varvec{j}}}^{\boldsymbol{*}}=\boldsymbol{ }\Delta {{\varvec{l}}}_{{\varvec{m}}}/{\varvec{r}}$$

The anisotropic elastic constants of isotropic materials are ${C}_{ijkl}$, and ${C}_{ijkl}^{*}$ for Epoxy and BaTiO_3_ respectively:

43 $${{\varvec{C}}}_{{\varvec{i}}{\varvec{j}}{\varvec{k}}{\varvec{l}}}={\varvec{\lambda}}{{\varvec{\delta}}}_{{\varvec{i}}{\varvec{j}}}{{\varvec{\delta}}}_{{\varvec{k}}{\varvec{l}}}+{\varvec{\mu}}{({\varvec{\delta}}}_{{\varvec{i}}{\varvec{k}}}{{\varvec{\delta}}}_{{\varvec{j}}{\varvec{l}}}{+{\varvec{\delta}}}_{{\varvec{i}}{\varvec{l}}}{{\varvec{\delta}}}_{{\varvec{j}}{\varvec{k}}})$$

44 $${{\varvec{C}}}_{{\varvec{i}}{\varvec{j}}{\varvec{k}}{\varvec{l}}}^{\boldsymbol{*}}={{\varvec{\lambda}}}^{\boldsymbol{*}}{{\varvec{\delta}}}_{{\varvec{i}}{\varvec{j}}}{{\varvec{\delta}}}_{{\varvec{k}}{\varvec{l}}}+{{\varvec{\mu}}}^{\boldsymbol{*}}{({\varvec{\delta}}}_{{\varvec{i}}{\varvec{k}}}{{\varvec{\delta}}}_{{\varvec{j}}{\varvec{l}}}{+{\varvec{\delta}}}_{{\varvec{i}}{\varvec{l}}}{{\varvec{\delta}}}_{{\varvec{j}}{\varvec{k}}})$$

45 $${\varvec{\lambda}}=\frac{{\varvec{Y}}}{2(1+{\varvec{v}})}$$

46 $${\varvec{\mu}}=\frac{{\varvec{Y}}{\varvec{v}}}{(1+{\varvec{v}})(1-2{\varvec{v}})}$$

where $\lambda \left({\lambda }^{*}\right)$ and ${\mu (\mu }^{*})$ are Lame constants^57^, $v$ is the Poisson ratio, and ${\delta }_{ij}$ is the Kronecker delta. Inserting Eqs. (43) and (44) into Eq. (37):

47 $${\varvec{\lambda}}{{\varvec{\delta}}}_{{\varvec{i}}{\varvec{j}}}{{\varvec{\varepsilon}}}_{{\varvec{k}}{\varvec{k}}}^{0}+2\boldsymbol{ }{\varvec{\mu}}{{\varvec{\varepsilon}}}_{{\varvec{i}}{\varvec{j}}}^{0}=\left[\left(\boldsymbol{ }{\varvec{\lambda}}-{{\varvec{\lambda}}}^{\boldsymbol{*}}\right){{\varvec{\delta}}}_{{\varvec{i}}{\varvec{j}}}{{\varvec{\varepsilon}}}_{{\varvec{k}}{\varvec{k}}}+2\left({{\varvec{\mu}}-{\varvec{\mu}}}^{\boldsymbol{*}}\right){{\varvec{\varepsilon}}}_{{\varvec{i}}{\varvec{j}}}\right]+\left({{\varvec{\lambda}}}^{\boldsymbol{*}}{{\varvec{\delta}}}_{{\varvec{i}}{\varvec{j}}}{{\varvec{\varepsilon}}}_{{\varvec{k}}{\varvec{k}}}^{\boldsymbol{*}}+2{{\varvec{\mu}}}^{\boldsymbol{*}}{{\varvec{\varepsilon}}}_{{\varvec{i}}{\varvec{j}}}^{\boldsymbol{*}}\right)$$

The equivalent eigenstrain ${\varepsilon }_{kl}^{0}$ and total strain can then be re-written as ${\varepsilon }_{kl}$:

48 $${{\varvec{\varepsilon}}}_{{\varvec{k}}{\varvec{l}}}^{0}={{\varvec{\delta}}}_{{\varvec{k}}{\varvec{l}}}{{\varvec{\varepsilon}}}_{0},\boldsymbol{ }\boldsymbol{ }{{\varvec{\varepsilon}}}_{{\varvec{k}}{\varvec{l}}}={{\varvec{\delta}}}_{{\varvec{k}}{\varvec{l}}}{\varvec{\varepsilon}}$$

where $\varepsilon$ is a function of ${\varepsilon }_{0}$^56^:

49 $${\varvec{\varepsilon}}=\frac{1+{\varvec{v}}}{3(1-{\varvec{v}})}{{\varvec{\varepsilon}}}_{0}=\boldsymbol{\alpha }{{\varvec{\varepsilon}}}_{0}.$$

Therefore, Eq. (47) is:

50 $$({3}\lambda + 2\mu ) \varepsilon_{0} = [3(\lambda - \lambda^{*} ) + 2(\mu - \mu^{*} )]\varepsilon + (3\lambda^{*} + 2\mu^{*} )\varepsilon^{*}$$

And the equivalent eigenstrain $\varepsilon_{0}$ is solved by Eq. (50):

51 $${{\varvec{\varepsilon}}}_{0}=\frac{3{{\varvec{\lambda}}}^{\boldsymbol{*}}+2{{\varvec{\mu}}}^{\boldsymbol{*}}}{\left(3\boldsymbol{ }{\varvec{\lambda}}+2{\varvec{\mu}}\right)\left(1-\boldsymbol{\alpha }\right)+(3{{\varvec{\lambda}}}^{\boldsymbol{*}}+2{{\varvec{\mu}}}^{\boldsymbol{*}})\boldsymbol{\alpha }}{{\varvec{\varepsilon}}}^{\boldsymbol{*}}$$

Therefore, the strain ${\varepsilon }_{kl}$ in the epoxy is expressed by:

52 $${{\varvec{\varepsilon}}}_{{\varvec{k}}{\varvec{l}}}=\frac{(3{{\varvec{\lambda}}}^{\boldsymbol{*}}+2{{\varvec{\mu}}}^{\boldsymbol{*}})\boldsymbol{\alpha }}{\left(3\boldsymbol{ }{\varvec{\lambda}}+2{\varvec{\mu}}\right)\left(1-\boldsymbol{\alpha }\right)+(3{{\varvec{\lambda}}}^{\boldsymbol{*}}+2{{\varvec{\mu}}}^{\boldsymbol{*}})\boldsymbol{\alpha }}{{\varvec{\varepsilon}}}^{\boldsymbol{*}}$$

### Interaction theory of BaTiO_3_-epoxy nanocomposite and microwave field

The major interaction between the dielectric material and the microwave field is the polarisation of the dipoles, i.e. under an oscillatory microwave field, the dipoles re-orient themselves with the external electric field component of the microwave field. Therefore, the BaTiO_3_ nanoparticles interact with the electric field component of the microwave field, and the magnetic field effect is considered negligible. The main contribution of microwave power loss in the multi-domain BaTiO_3_ nanoparticles originates from the piezoelectric domain wall movement^58^. Compared with the BaTiO_3_ nanoparticles, the dielectric loss tangent of epoxy represents the ability to dissipate the absorbed energy is relatively smaller (in the range of 10^−3^ to 10^−4^)^59^. The mobility of the polar segments of polymer decreased significantly when the frequency reaches the GHz range^60^. Therefore, the dielectric loss in BaTiO_3_-Epoxy nanocomposites observed at a higher frequency (e.g. microwave at 2.45 GHz) is mainly attributed to the BaTiO_3_ nanoparticles.

The behaviour of the electric and magnetic components of the microwave field is governed by Maxwell’s equations coupled with boundary conditions^61^.

53 $$\nabla \times \mathbf{H}=j\omega \varepsilon \mathbf{E}$$

54 $$\nabla \times \mathbf{E}=j\omega \mu \mathbf{H}$$

55 $$\nabla \cdot \mu \mathbf{H}=0$$

56 $$\nabla \cdot \varepsilon \mathbf{E}=\rho$$

where $\mathbf{H}$ and $\mathbf{E}$ are the varying magnetic (A/m) and electric field strength (V/m), respectively, $\omega$ is the angular frequency (rad/s), $\varepsilon$ is the complex relative permittivity, $\mu$ is the complex relative permeability, and $\rho$ is the charge density. The first two equations, so-called Ampere’s law and Faraday’s law, correlate the changes in the electric or magnetic field. The last two equations are Gauss’s laws, which represents the net magnetic flux out of a region. It must be zero while the net electric flux out of a region is related to the charge density within the region^62^. The power absorbed by the material in the microwave field is dependent on many factors such as shape, size, and location of the material. For a relatively thin material that fully penetrated by the microwave field, the power dissipated $P$ (W/m^3^) as heat in the material under microwave field is expressed based on Maxwell’s Equation as follows^63^:

57 $$P = \omega \left( {\mu_{0} \mu^{\prime \prime } H_{rms}^{2} + \varepsilon_{0} \varepsilon^{\prime \prime } E_{rms}^{2} } \right)$$

where the first term $\mu_{0} \mu^{\prime\prime}H_{rms}^{2}$ describes the dissipation of power due to magnetic field and can be neglected for a non-ferrite material, e.g. BaTiO_3_, $\omega =2\pi f$, $f$ is the frequency of the applied microwave field (= 2.45 GHz), ${\varepsilon }_{0}$ is the dielectric permittivity of the free space, $\varepsilon^{\prime \prime }$ represents the imaginary part of the complex dielectric permittivity, and ${E}_{rms}$ is the root-mean-square value of the local electric field strength (V/m). Therefore, Eq. (57) is simplified to:

58 $$P = \omega \varepsilon_{0} \varepsilon^{\prime \prime } E_{rms}^{2}$$

However, the explicit evaluation of the absorbed power by the material inside the oven cavity is difficult to obtain due to the non-uniformity of the microwave distribution, and hence a varying electric field strength. The power absorbed by the material ${P}_{absorb}$, is expressed as follows:

59 $${P}_{absorb}={P}_{input}-{P}_{reflected}-{P}_{oven},$$

where ${P}_{input}$ is the input power from the magnetron, ${P}_{reflected}$ is the power reflected and attenuated, and ${P}_{oven}$ is the power absorbed by the cavity wall, waveguide, and magnetron. The increased temperature due to microwave heating also has a significant impact on the dielectric properties of the material, which in turn affect the degree of interaction of the nanocomposite with the microwave field. In previous studies, an empirical approach has been proposed to estimate the absorbed power. It is assumed that the amount of microwave energy absorbed by the material/field interaction is converted into heat during the exposure, and is expressed as follows^64,65^:

60 $$\Sigma mc\Delta T = 2\pi f\varepsilon_{0} \varepsilon^{0} E_{rms}^{2} Vt$$

where $m$ is the mass of the material, $C$ is the specific heat capacity of the material, $\Delta T$ is the temperature change, $V$ is the volume of the material, and $t$ is the microwave exposure time.

The energy transferred into the material is also significantly determined by the depth up to which the microwave field travels into it. The degree of microwave penetration in the material is defined by a factor, Penetration depth (${d}_{p}$)^66,67^, which is the distance between the surface of the material and the place inside the material where the magnitude of the field strength drops to ${e}^{-1}$ (36.8%) of that at the surface. The penetration depth ${d}_{p}$ neglecting the effects from the magnetic field for a non-ferrite material is expressed as follows^65^:

61 $${d}_{p}=\frac{c}{\omega {\sqrt{2{\varepsilon }^{^{\prime}}}\left[\left(\sqrt{1+{\left(\mathrm{tan}\delta \right)}^{2}}-1\right)\right]}^{1/2}}$$

where $c$ is the velocity of light, ${\varepsilon }^{^{\prime}}$ is the real part of the complex dielectric permittivity, and $\mathrm{tan}\delta$ is the tangent loss of the material, $\tan \delta = \varepsilon \prime \prime /\varepsilon \prime$. Generally speaking, the microwave power decays more quickly in lossy materials with higher $\mathrm{tan}\delta$ due to energy dissipation hence a lower penetration depth.

From Maxwell’s equation, it is noted that the energy of the microwave field is proportional to the square of the amplitude, which represents the maximum strength of its electric and magnetic components. For a continuous sinusoidal microwave field in the air of an empty cavity, the average intensity $I$ is the power carried by the microwave field per unit area, and is expressed as follows^68^:

62 $$I=\frac{c {\varepsilon }_{0}{E}_{m}^{2}}{2},$$

where ${E}_{m}$ is the maximum electric field strength, and the root-mean-square value of the electric field is:

63 $${E}_{rms}= \sqrt{{E}_{m}^{2}/2}.$$

Equations 62 and 63 above could only be used to calculate the electric field strength in an empty oven. When a sample is placed in the multimode microwave oven (our case), the electromagnetic field is considered to be a superposition of multiple plane waves penetrate the material from different directions. Regardless of the differences in each mode, the superposition of all the waves could be assumed to have an approximately uniform and constant distribution pattern in the material within the localised region^69^. Assumptions are made that the absorbed microwave energy is converted to heating in the material with neglected surface heat loss, thermal diffusion, and energy absorbed by the oven (e.g. magnetron and cavity wall). Therefore, the energy absorbed by the material is corresponding to the temperature rise of the material, and it is expressed by re-arranging Eq. (60):

64 $$E_{{{\text{internal}}}} = \sqrt {\frac{mc\Delta T}{{2\pi f\varepsilon_{0} \varepsilon^{\prime \prime } \Delta t}}}$$

It should be noted that the internal electric field is only localised in the material and is different from that in the surrounding cavity. Under the same assumptions that the material is surrounded by a uniform microwave field, incident microwaves are mostly transmitted and absorbed by the material while some are reflected. It is also assumed no reflected wave at the interface. An expression is presented as follows^70^:

65 $${Q}_{absorb}V={P}_{absorb}=\rho C\frac{\Delta T}{\Delta t}V={\varepsilon }_{0}\cdot {E}_{rms.external}^{2}\cdot c\cdot A\cdot \xi \cdot\Gamma$$

where $V$ is the volume of the material, $\rho$ is the density of the materials, $c$ is the phase velocity of the microwave ($3\times {10}^{8}$ m/s)^71^, $A$ is the surface area, $\xi$ is the absorption coefficient that represents the fraction of the absorbed power that generates heat, and $\Gamma$ is the transmission coefficient. Solving the equation, the time-averaged strength of the external electric field ${E}_{rms, external}$ obtained by:

66 $${E}_{rms, external}=\sqrt{\frac{\rho Ca\Delta T}{3\Delta tc{\varepsilon }_{0}\xi\Gamma }}$$

The power absorption coefficient $\xi$ is expressed as follows^72^:

67 $$\xi =\frac{{P}_{absorb}}{{P}_{0}}=1-{e}^{-2a/{d}_{p}}$$

where ${P}_{0}$ is the incident power at the surface, and $a$ is the thickness of the BaTiO_3_-epoxy sample. The transmission coefficient, $\Gamma$, is the energy fluxes per unit time and unit area at the interface. It is assumed that the microwave incident is normal to the material surface as the energy flux comes from all directions within the cavity, and the transmission coefficient $\Gamma$ is simplified as^71^:

68 $$\Gamma =\frac{4({n}_{1}/{n}_{2})}{{\left[\left({n}_{1}/{n}_{2}\right)+1\right]}^{2}}$$

where ${n}_{1}$ and ${n}_{2}$ are the refraction index of air and the material, respectively. The above equations are employed to theoretically obtain an estimate of the average and peak electric field strengths of the incident microwave with a given cavity size. Subsequently, the absorbed microwave power by the nanocomposite has been estimated.

## Materials and experiments

### Materials and fabrication

The epoxy used in this study was Araldite LY1564, a diglycidyl ether of bisphenol A (DGEBA) and the curing agent was Aradur 3487, an amine hardener, supplied by Huntsman, UK. This epoxy resin system has relatively low viscosity and high flexibility mainly for aerospace and industrial structural composites parts. The coupling agent for surface functionalisation selected in this study was 3-glycidoxypropyl trimethoxysilane (3-GPS) supplied by Sigma-Aldrich, US. Hydrogen peroxide (H_2_O_2_, 30%) and acetic acid (C_2_H_4_O_2_, 99.9%) used as functionalisation aids were supplied by Sigma-Aldrich, US, and the ethanol (C_2_H_6_O, 99.9%) used for BaTiO_3_ dispersion by Fisher Scientific International, Inc., UK. BaTiO_3_ powders were supplied from Nanostructure & Amorphous Materials Inc., US. The general properties of the powders measured by the manufacture, detailed in Table 1, below^73^. All the chemicals except BaTiO_3_ powders were used as received without further treatment.

**Table 1:** General properties of BaTiO_3_ nanoparticles^73^.

Size	200 nm
Phase	Tetragonal
Specific surface area (SSA)	6–7 m2/g Spherical
Density	5.85 g/cm3

**Figure 6:**
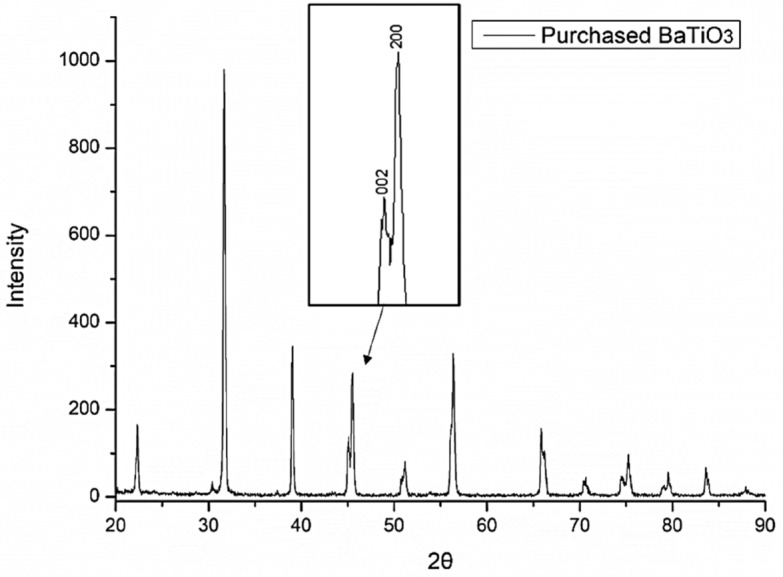
X-ray diffraction patterns of purchased BaTiO_3_ powders. The inset shows the split peaks of (002) and (200) indicating the tetragonal phase.

The phase composition of BaTiO_3_ powders has been confirmed by X-Ray diffraction (XRD) using a Siemens D5005 X-Ray Diffractometer with Cu-Kα radiation (wavelength λ of 1.54 Å, at 40 kV and 30 mA). The scanning speed was 0.5°/s in the range of 20° to 90°. The peaks analysis was performed by data analysis software Origin using a Lorentzian curve fit function. Figure 6 shows a well-defined perovskite structure of BaTiO_3_ without a noticeable second phase. The split peaks between 44°and 46° of the 2θ angle correspond to reflections (002) and (200) confirms the tetragonal phase of BaTiO_3_^74^.

#### Surface functionalisation of BaTiO_3_ and characterisation

**Figure 7:**
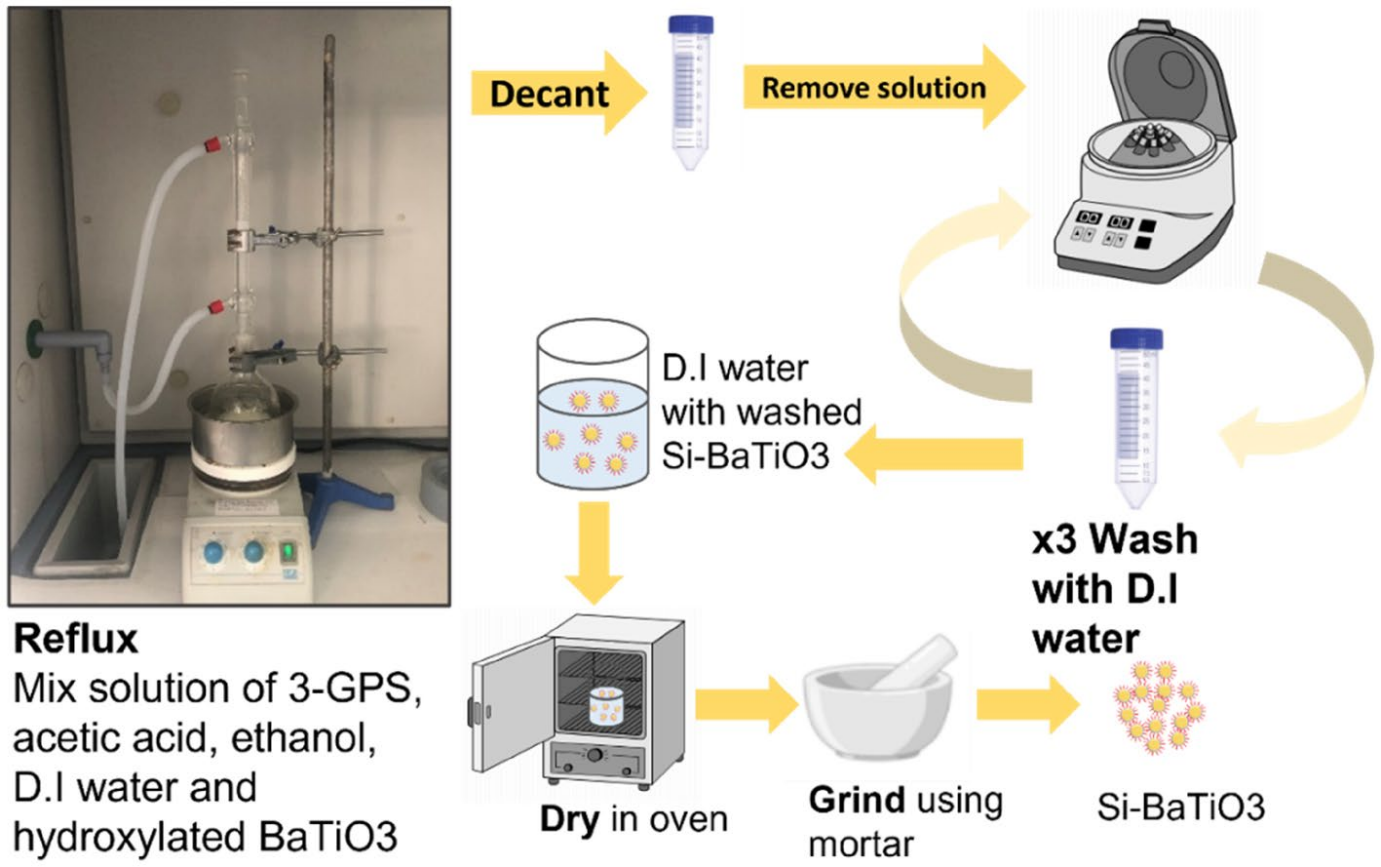
Schematic diagram of the functionalisation process of BaTiO_3_ with 3-GPS.

The BaTiO_3_ powders were prepared using combustion method, therefore, firstly they were pre-treated in H_2_O_2_ for hydroxylation process to add hydroxyl group (-OH) to the surface^75^: 10 g BaTiO_3_ nanoparticles were added into a 230 mL solution of H_2_O_2_ in a round bottom flask. The mixture was then sonicated in an ultrasonic bath for 30 min and then refluxed at the boiling temperature of 30% H_2_O_2_ solution at 108 °C^76^ at 100 rpm using a mechanical stirrer for 6 h to facilitate the process by heating without losing H_2_O_2_. The nanoparticles were retrieved by centrifuging the resulting solution at 4500 rpm for 15 min, and washed three times with deionized water. The achieved BaTiO_3_ nanoparticles were dried in an oven at 80 °C for 24 h. The reflux and particle retrieving processes were similar as in the surface functionalisation with 3-GPS as shown in Fig. 7. 3-GPS was then applied to BaTiO_3_ nanoparticles to improve the processability and filler dispersion in nanocomposites; the solution of 1 wt.% of 3-GPS with respect to BaTiO_3_ was prepared. 150 mL aqueous solution of ethanol and deionized water (9:1) was firstly mixed in a beaker. Adding the acetic acid drops using a pipette and stir vigorously after each drop until the pH value of 3.5–4 measured by a METTLER TOLEDO pH meter was reached, stirred vigorously again for 3 min to form a clear solution. The low pH values of the solution facilitate the silane functionalisation process^77^. After the addition of the 0.1 g 3-GPS solution to the acidified solution using a pipette, the mixture was left in an ultrasonic bath for 30 min to form a homogenous solution. 10 g hydroxylated BaTiO_3_ powders was then added to the silane solution, and mixed under ultrasonic bath for 10 min for better filler wetting. Finally, the mixture was refluxed at the boiling temperature of ethanol, 78 °C^78^, at 100 rpm using a mechanical stirrer for 6 h using a silicone oil bath over a hotplate. After refluxing, the BaTiO_3_ was washed three times with deionized water and retrieved using centrifugation at 4500 rpm. The silane treated BaTiO_3_ (Si-BaTiO_3_) powders were dried at 110 °C for 24 h to avoid any condensation of silanol groups at the surface. In the end, the powders were crushed in a mortar and pestle for the nanocomposites preparation. The size distribution of Si-BaTiO_3_ particles was analysed in its epoxy nanocomposite form. The surface functionalisation didn’t significantly affect the particle size as shown in the SEM images and it is approximately 200 nm (discussed in "Scanning electron microscope (SEM)." section).

The silane-treated powders were characterised by Thermogravimetric analysis (TGA) and Fourier transform infrared (FTIR) spectrometry to confirm the presence of the functional group in the following sections.

##### Fourier transform infrared (FTIR) spectrometry

**Figure 8:**
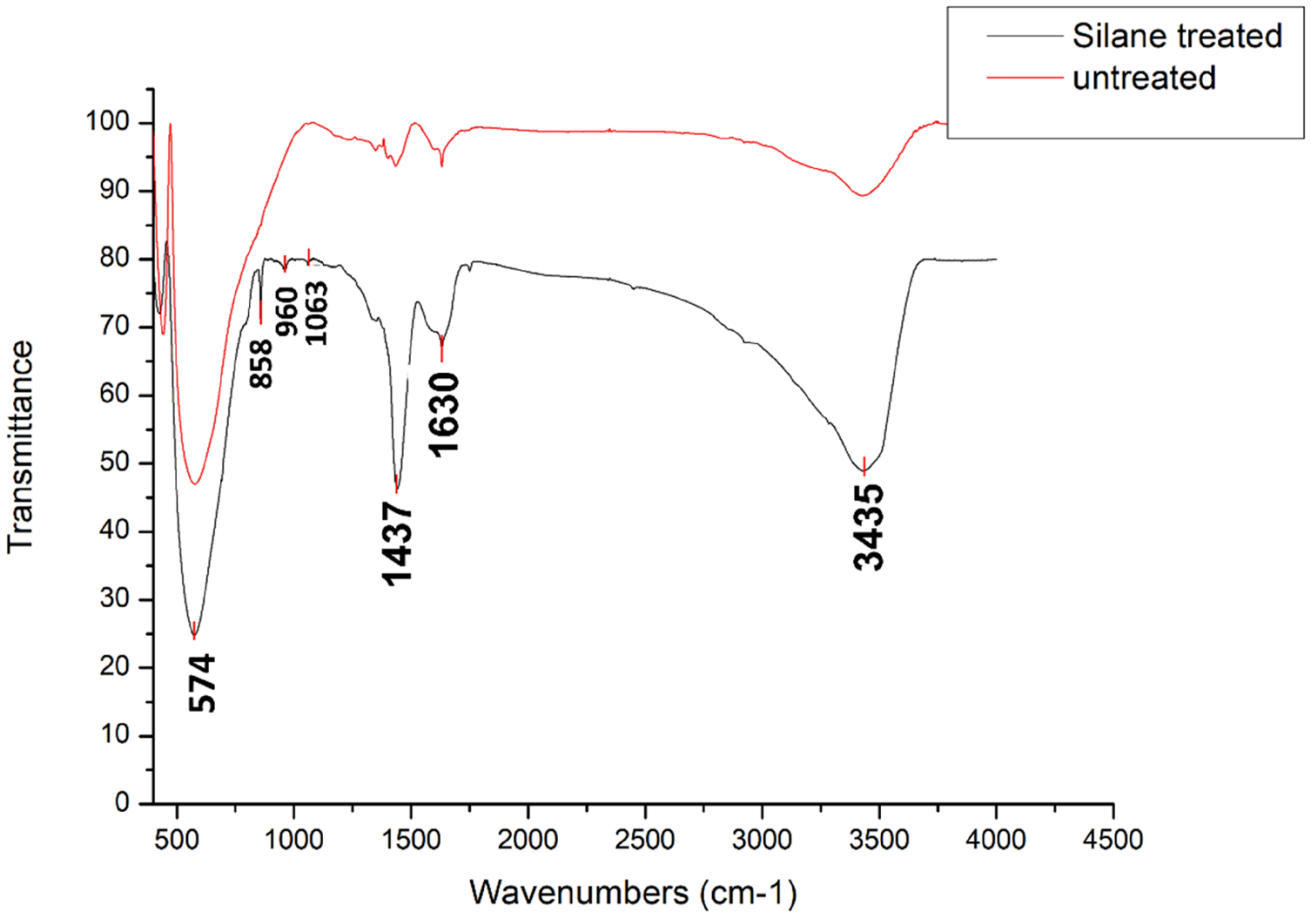
FTIR spectra of Si-BaTiO_3_ (black) and untreated (red) BaTiO_3_ powders.

FTIR is performed to detect the silane functional groups on the modified BaTiO_3_ powders in transmission mode using a Jasco FT/IR-6200 in the range of 400–4000 cm^−1^ with a resolution of 2 cm^−1^ at room temperature. The FTIR spectra of Si-BaTiO_3_ and untreated powders are presented in Fig. 8. The peaks at 1437 cm^−1^ and 1630 cm^−1^ are due to the small trace of BaCO_3,_ and physically absorbed water on BaTiO_3_ powders from the combustion fabrication process according to the manufacturer^79^. The bands at 3200–3700 cm^−1^ are attributed to hydroxyl groups OH (from Si–OH group) stretching vibrations. The bands between 850 and 1250 cm^−1^ are representations of Si–O, Si–O–C_2_H_5_, and Si–O vibrations. These new peaks confirm that the silane group are successfully grafted onto the surface of BaTiO_3_ powders^80^.

##### Thermogravimetric analysis (TGA)

**Figure 9:**
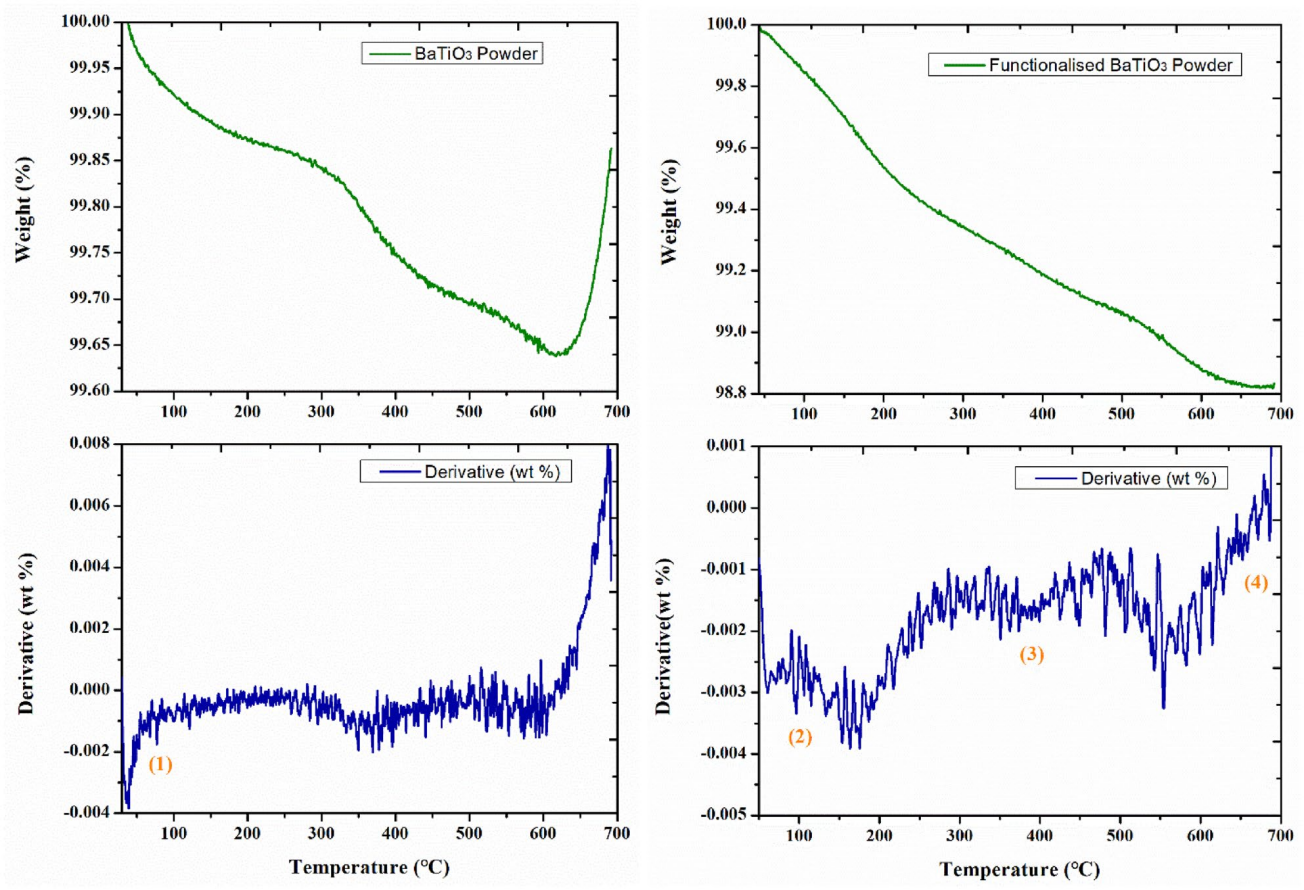
TGA curves of untreated and Si-BaTiO_3_ powders. Green lines indicate the weight percentage of the powders versus temperature, and blue lines the rate of change of the weight percentage versus temperature.

The amount of organic silane group grafted onto the surface of BaTiO_3_ powder was obtained by TGA using a TA instruments Q-500 at a rate of 20 °C/min under a dry nitrogen gas flow rate of 100 ml/min to 700 °C, shown in Fig. 9 for untreated and Si-BaTiO_3_ powders. The weight loss percentage indicated as the lowest point of the green line is 0.35% of untreated BaTiO_3_ and 1.2% for treated. Therefore, the amount of 3-GPS grafted onto the BaTiO_3_ surface was calculated to be approx. 0.85%, which is close to the previously designed 1% in the surface functionalisation process. The weight curve of 3-GPS treated BaTiO_3_ powders showed three well-defined degradations as labelled in the figure. Based on the FTIR results, the absorbed water is removed between 50 to 150 °C, (1) and (2) in the figure. The degradation step from 240 to 450 °C represents the free hydroxyl groups (–OH) and/or silane molecules that remained on the surface, labelled as (3). The pyrolysis of surface silane compounds was observed at around 565 °C, labelled as (4)^79,81^. The increase in weight between 650 and 700 °C is only observed in the untreated powder and it is attributed to the impurities in the as-received powder (supplied from US Research Nanomaterials Inc) reacting with nitrogen.

#### Fabrication of BaTiO_3_ nanocomposites

**Figure 10:**
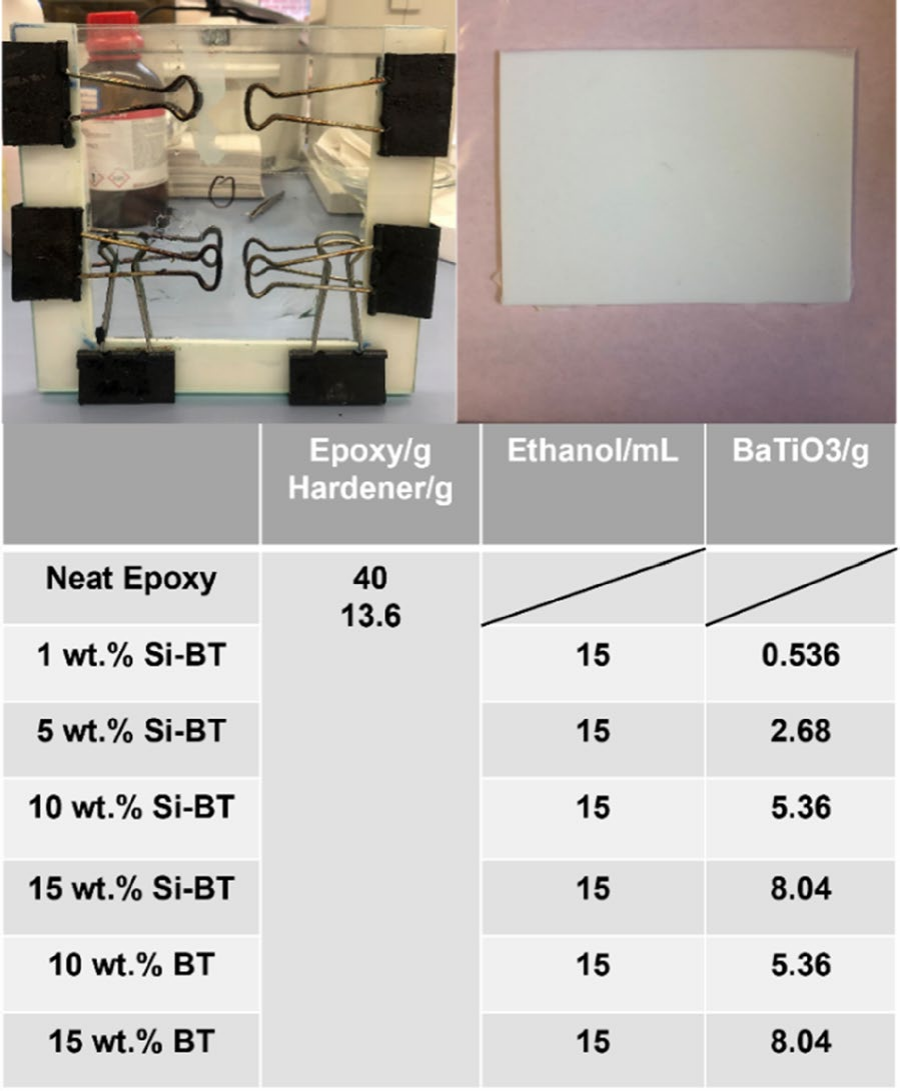
Glass mould used for nanocomposite fabrication and the cured nanocomposite samples.

The epoxy resin nanosuspension with 1, 5, 10, 15 wt.% Si-BaTiO_3_ nanoparticles and 5, 10, 15 wt.% untreated BaTiO_3_ nanoparticles were prepared as follows: Firstly, the weighed amount of BaTiO_3_ powders was added to ethanol and sonicated with an ice water bath for 2 min with a 10 s pulse to form a homogenous solution. Then a weighed amount of epoxy and the previous mixed solution were blended in a beaker using a mechanical stirrer at 300 rpm and 80 °C overnight under the fume hood to gradually remove the ethanol without precipitation of the particles. The mixture was weighed before and after the previous step to ensure the complete removal of ethanol. The curing agent was then added to the mixture with a weight ratio recommended by the company and stirred for a further 3 min. Finally, the mixture was placed in the vacuum oven at 30 °C for 1 h to remove bubbles at 29 inHg and achieve complete removal of ethanol. The whole mixture was poured into a mould made of two pieces of glass clamped with a 3 mm silicone gasket in-between, as shown in Fig. 10. A uniform thickness of each sample was achieved with the assistance of this type of glass mould. They were then cured in the oven for 8 h at 80 °C as prescribed by the manufacturer, then cooled down to room temperature. The final samples were of size 160 × 140 × 3 mm^3^, and cut to different sizes using a precision cut-off machine BRILLANT 220.

### Field induced experimental methods

#### General material characterisations

The morphological characterisation of the nanocomposites, post cure, with different weight loading between 0 wt.% and 15 wt.% were carried out using a field emission scanning electron microscope TESCAN Vega 3. A thin layer of gold is coated to each sample before testing to enable better conductivity. The thermal characterisation of the cured nanocomposites was studied using a differential scanning calorimeter (DSC Q200, TA Instruments). The DSC measurements were performed in a nitrogen flow (50 mL/min). Samples 5 × 3 × 1 mm^3^ were prepared using a Buehler ISOMAT low speed saw. The samples were first heated from 40 to 220 °C at 20 °C/min. The measured DSC curves of all epoxy nanocomposites presented a distinct exotherm peak. Thereafter, the second run at a heating rate of 20 °C/min was conducted up to 220 °C after cooling down from the first scan. The glass-transition temperature (${T}_{g}$) was determined using the inflection-point method following the IS/DIS 11,357-2. A tangent was drawn at the point of inflection in the DSC spectra from the second run, and the ${T}_{g}$ was the midpoint between onset and end-set of the drawn tangent.

**Figure 11:**
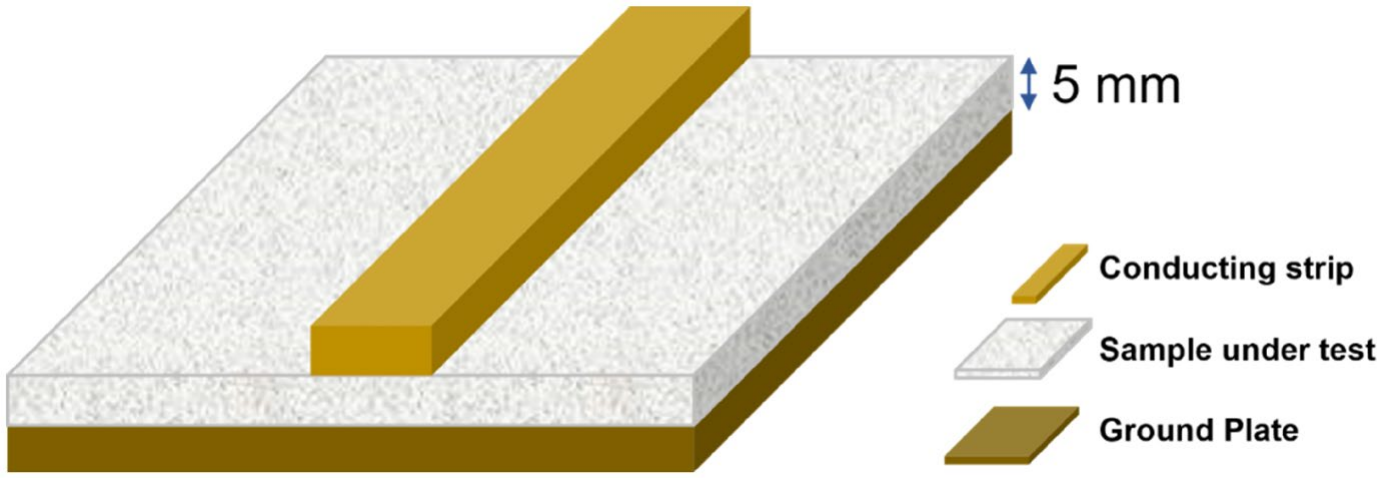
Schematic illustration of the layout of sample for microstrip line methods.

The complex relative permittivity and permeability of the neat epoxy and the nanocomposites at different weight loading were measured with increasing frequency from 0.25 to 4.50 GHz at a 0.25 step increment using a vector network analyser (VNA) with microstrip methods^82^. Flat plates samples of size 50 × 50 × 3 mm^3^ were prepared as substrates for conductive lines as shown in Fig. 11. They were then attached to a ground metal plate at the bottom. The dielectric constants of the samples were then measured using the VNA. The resonant approach can provide a more accurate values of the dielectric properties compared with the transmission line method. However, the resonant methods could only measure the dielectric properties at a single frequency instead of measuring in the frequency range^83^.

#### Field-induced strain measurements

The microwave field was selected to be the external stimulator as BaTiO_3_ exhibit a peak in a dielectric loss at the microwave frequency range. Most importantly, the design of the experiment becomes more feasible with remote stimulation from the microwave field owing to the spacious cavity. The mechanical strain evolution in the nanocomposite was investigated under a microwave field within a temperature-controlled microwave cavity Panasonic NN-SF464MBPQ running at 2.45 GHz and cavity size of 354 × 338 × 230 mm^3^. In contrast with the conventional one, this oven, equipped with inverter technology, has a circuit board that replaces the transformer, hence the output power can be adjusted linearly by varying the pulse width to ensure a more precise and continuous microwave exposure^84^. The unique flat-bed design of this model is equipped with a stationary ceramic plate that allowed more space to place the sample and its holder.

The strain field introduced by the BaTiO_3_ nanoparticles to the surrounding epoxy, activated by microwave field, was studied by real-time strain measurements on the surface of the nanocomposite samples with the incorporation of Fibre optic sensors utilising fibre Bragg gratings (FBG) technique. The sensors were placed apart in equal distance from one another within a 90 mm straight line. This is theoretically aligned with the microwave’s half wavelength (~ 60 mm) as also was experimentally observed during real-time temperature measurements. The sensors were located to ensure overlapping with at least three nodes and antinodes of the microwave cycle. The FBG sensors were fabricated by a periodic intense laser light applied onto the core of an optic fibre. The laser light exposure introduced a permanent increase in the refractive index of the fibre’s core and a fixed modulation was created subsequently. Each FBG was approx. five mm long with a grating period of one micron. Two arrays with three FBGs for each sample were fabricated. FBG arrays were then adhesively bonded to the surface of two geometrically identical 135 × 10 × 3 mm^3^ samples made of Si-BaTiO_3_ epoxy nanocomposites. The strain array was bonded to the surface by adhesives at the FBG regions while the temperature array is firstly packaged into a capillary glass tube, and then bonded parallel to the strain array. The arrays were placed at the same location with the same distance in-between. The strain experienced by the samples was transferred to the FBG sensors, and the measurements were also affected by the temperature. Therefore, the temperature array only measured the temperature change, and compensated the strain measurements accordingly.

**Figure 12:**
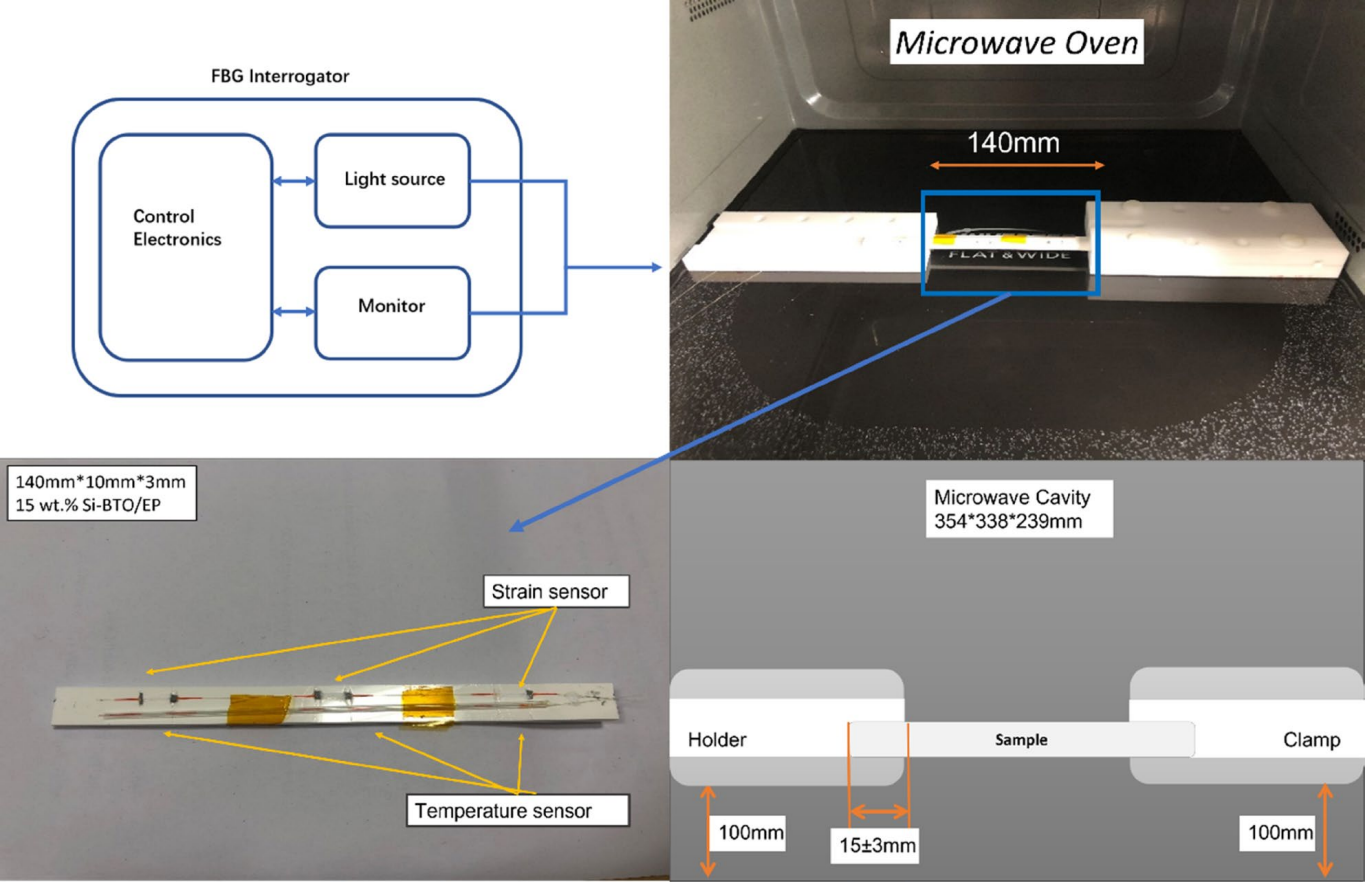
Schematic illustration of the sample of epoxy nanocomposite with BaTiO_3_ on the PTFE holder in the microwave oven with surface bonded FBG arrays connected to an interrogator.

A preliminary test via a FLIR One Pro LT Thermal Camera was carried out to inspect the temperature change in different samples under different microwave power levels. Samples were placed at a designed location as shown in Fig. 12. The exposure time was then carefully selected based on the heating profiles of each sample to control the temperature of samples well below ${T}_{g}$ during the exposure to avoid any interference in strain measurements from the post-cure shrinkage. The sample was clamped on one end by a designed sample holder made of polytetrafluoroethylene (PTFE) to ensure minimum interaction with the microwave field owing to its extremely low dielectric loss. The size and location of the load inside the cavity were two primary factors that affect the microwave field distribution^84^. To control the microwave field distribution, the sample and the holder were placed at the designed location shown in the figure, for all tests. The power level of 100 W and 440 W were initially selected to avoid temperature surges in samples within a short period of time. Exposure time was set to be 650 s for 100 W and 148 s for 440 W to limit the temperature below ${T}_{g}$ (80 °C) based on the preliminary results obtained by the thermal camera.

Neat epoxy, BaTiO_3_ nanoparticles, and adhesive used for bonding FBGs possess different thermal expansion coefficients (CTE) and microwave heating patterns. Microwave field interaction with the neat epoxy and adhesive have been investigated, thereafter. Two arrays with five FBGs of five mm long and one micron grating period were fabricated for each test. First, the adhesive response under microwave radiation was studied by two arrays of FBGs adhesively bonded to the surface of a PTFE block. It is assumed that measured strain and temperature change are solely due to the microwave heating of adhesive as PTFE has neglectable thermal response (temperature rise) under the microwave. The PTFE block is placed in the microwave oven at the designed location to locate the first FBG sensor on the left end at the same location as the one in the previous exposure on the nanocomposites. The tests have been performed under 100 W for 650 s and 440 W for 110 s. Data was recorded on the interrogator 10 s prior to the microwave exposure for each run to ensure no data is missing after the microwave starts. The effect of neat epoxy with the FBG sensors bonded to its surface was investigated as a controlled group to the test of 15 wt.% nanocomposites at 10 W for 600 s and 440 W for 60 s. Neat epoxy sample geometrically identical with 15 wt.% nanocomposites in the nanocomposite’s exposure test was placed in the designed location with surface-bonded arrays of FBGs. The whole measurements from adhesive and the neat epoxy were compared with the results from the previously performed test on the nanocomposites for better distinguishing the BaTiO_3_-epoxy nanocomposite’s response to the microwave exposure.

#### Raman spectroscopy

Raman characterisation was carried out to investigate residual stress, any indication of remnant strain field or crystal structure change in BaTiO_3_ nanoparticles embedded epoxy post microwave exposure. Neat epoxy samples and 15 wt.% BaTiO_3_ epoxy nanocomposites with the identical geometric dimension of 135 × 10 × 3 mm^3^ were characterised before and after 440 W microwave radiation of 110 s. The Raman spectra were obtained using a Raman spectroscope Horiba Scientific LabRAM HR with a 514 nm excitation wavelength laser for all samples.

## Results

### General material characterisations

#### Scanning electron microscope (SEM)

**Figure 13:**
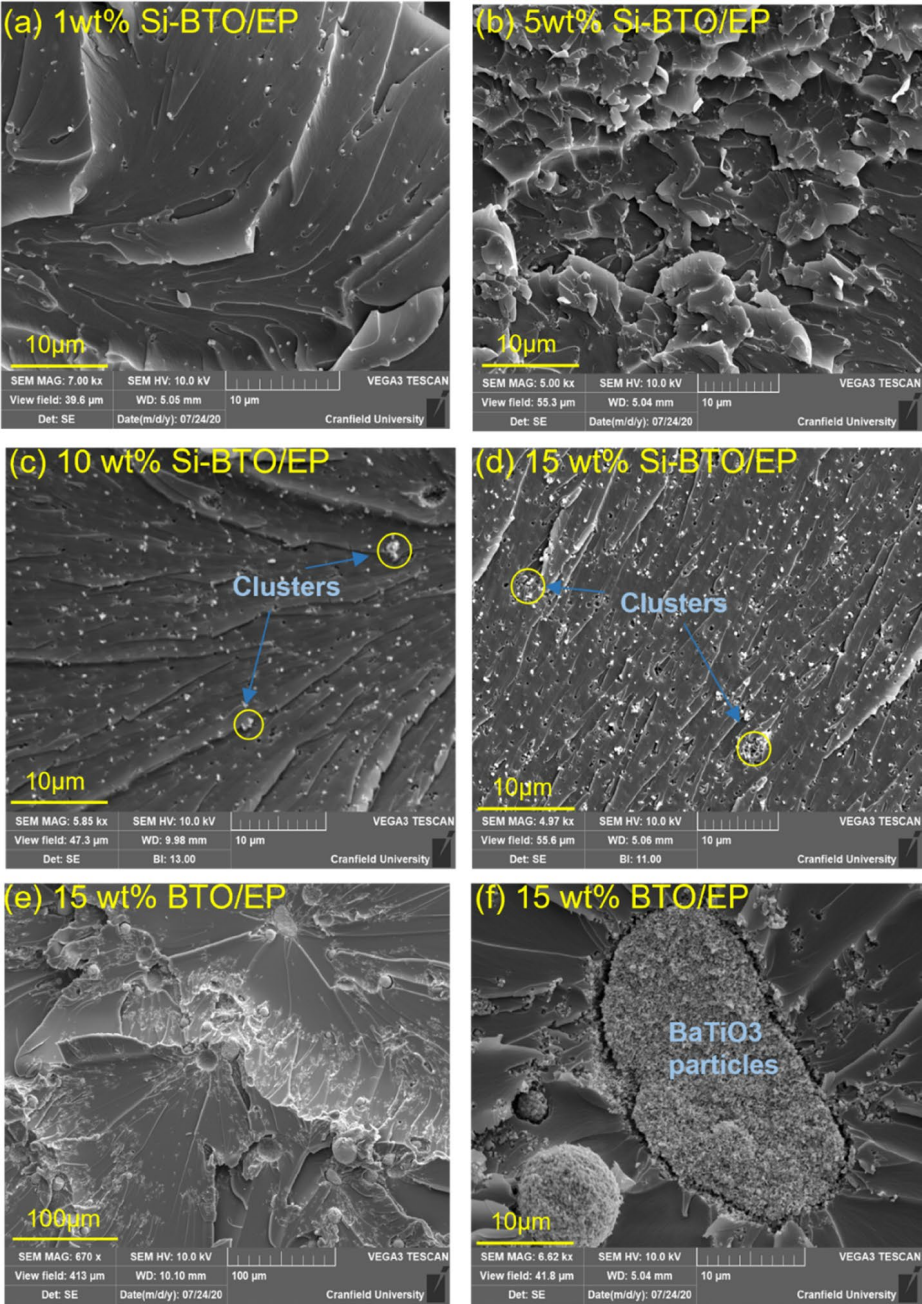
SEM images of fracture cross of epoxy nanocomposites with BaTiO_3_ at different weight loading, (**a**)–(**d**) images of 1 wt.%, 5 wt.%, 10 wt.% and 15 wt.% Si-BaTiO_3_/Epoxy nanocomposite samples. (**e**) and (**f**) 15 wt.% untreated-BaTiO_3_-epoxy nanocomposite at lower (670x) and higher (6620x) magnification.

A silane coupling agent 3-GPS was introduced to modify the BaTiO_3_ particles in this work to achieve a finer dispersion in epoxy. To examine its effectiveness, the dispersion and distribution of the nanoparticles at epoxy with different loadings have been characterised using the SEM. Different levels of aggregation events due to increased weight fraction of particles and enhancement of distribution due to surface functionalisation were present:

Figure 13a–f presents the SEM images of the fractured cross-section of the silane functionalised and non-functionalised BaTiO_3_ epoxy composites at various wt.%. It is shown that the nanoparticles are uniformly dispersed with the aid of surface functionalisation. As shown in Fig. 13a, the silane-treated particles are of the similar size of 200 nm as pristine BaTiO_3_ particles. There is no apparent aggregation of Si-BaTiO_3_ particles until small-scale clusters become visible in Fig. 13c,d when the loading of BaTiO_3_ reaches 10 wt.% and over, due to nanofillers agglomeration as circled in Fig. 13c,d. Overall, the majority of the Si-BaTiO_3_ particles were finely dispersed compared with the untreated ones. Figure 13e,f show images of the composites with 15 wt.% non-functionalised BaTiO_3_ particles at different magnification. In contrast with the functionalised BaTiO_3_ at the same weight loading 15 wt.% shown in Fig. 13d, the 15 wt.% untreated BaTiO_3_ exhibit severe agglomeration to form large clusters that have left large-scaled lamellar structures. The efficacy of the silane functional group is in good agreement with works conducted by others^85–87^.

#### DSC

The DSC was conducted to examine the effect on curing extent and ${T}_{g}$ of cured epoxy nanocomposites with varying weight fractions of Si-treated and untreated BaTiO_3_ nanoparticles.

**Figure 14:**
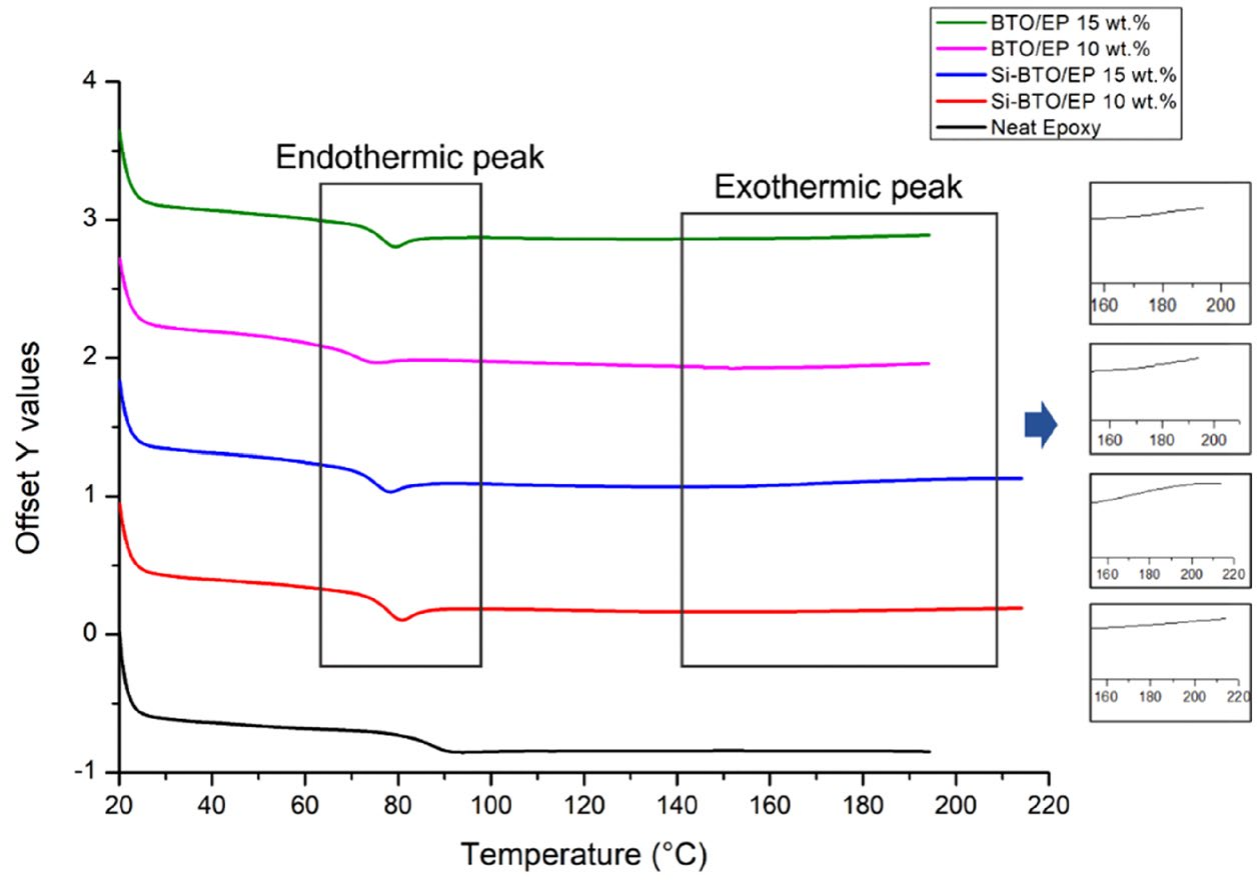
DSC spectra of neat epoxy and nanocomposites with 10 wt.% and 15 wt.% of untreated BaTiO_3_ (pink and green) and Si-BaTiO_3_ (red and blue). Weak endothermic peak around Tg and weak exothermic peak from 160 to 220 °C are identified.

Figure 14 shows the DSC spectra of the neat epoxy and nanocomposites at various BaTiO_3_ content as an attempt to provide a comparative analysis between the variation of Tg in the different multi-material systems. As observed from the insets, the DSC spectra of epoxy nanocomposites exhibit insignificant *bumps* denoted as exothermic peaks representing the post-curing process due to the impeded curing by the existence of BaTiO_3_ nanoparticles, i.e. indicating the nanocomposite material is well cured with the current weight loadings of the BaTiO_3_ particles. The ${T}_{g}$ transition for the coloured lines that represent nanocomposites are shifted slightly to lower temperatures compared with the black line of neat epoxy which is evidence of reduced ${T}_{g}$ as the nanoparticles hindered the curing process of the epoxy resin^88^. Furthermore, the weak endothermic peaks that emerge on the spectra represent the relief of the stress introduced during processing and handling or the thermal history. The thermal history and residual stress were removed from the first run of heating and the samples were post-cured during the first DSC analysis up to 200 °C. The true ${T}_{g}$ of each cured nanocomposite sample was calculated based on the DSC spectra of a second run under the same condition.

**Figure 15:**
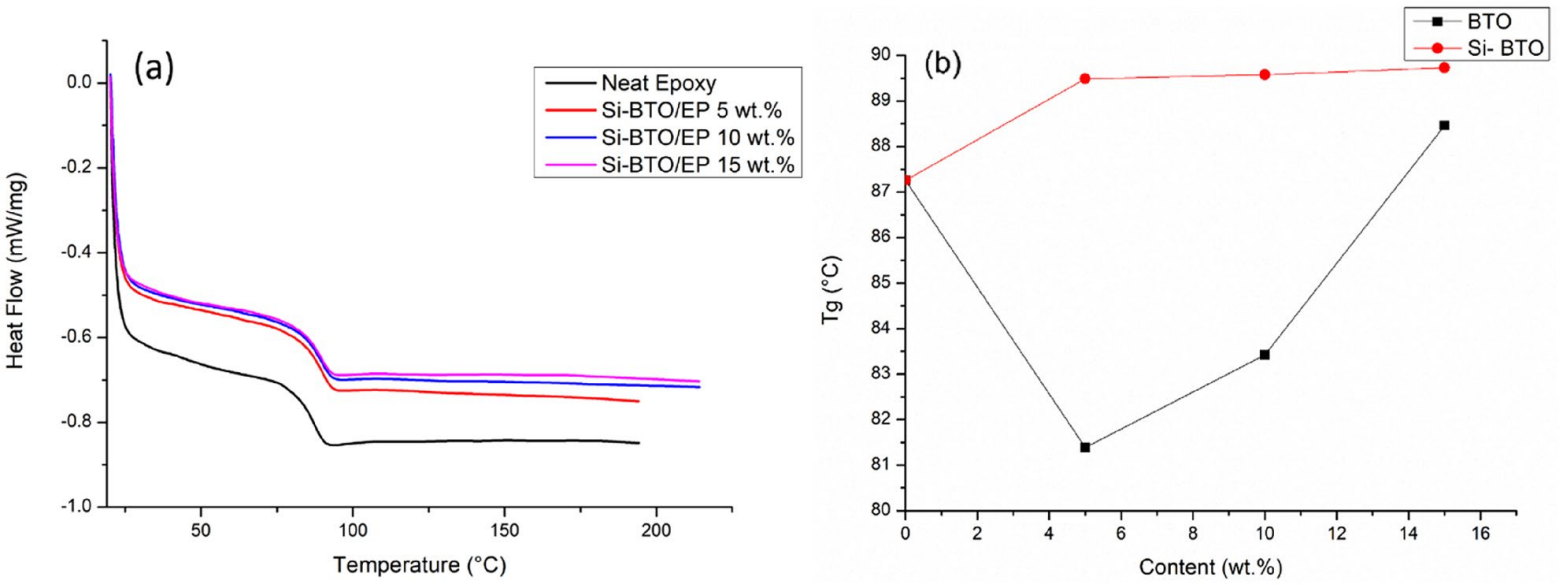
(**a**) DSC spectra of the neat epoxy (black) and Si-BaTiO_3_-Epoxy nanocomposite samples at 5 wt.% (Red), 10 wt.% (Blue), and 15 wt.% (Pink), (**b**) Tg temperature as a function of weight loading of Si-BaTiO_3_ (Red) and untreated BaTiO_3_ (Black) of the epoxy nanocomposites samples.

**Figure 16:**
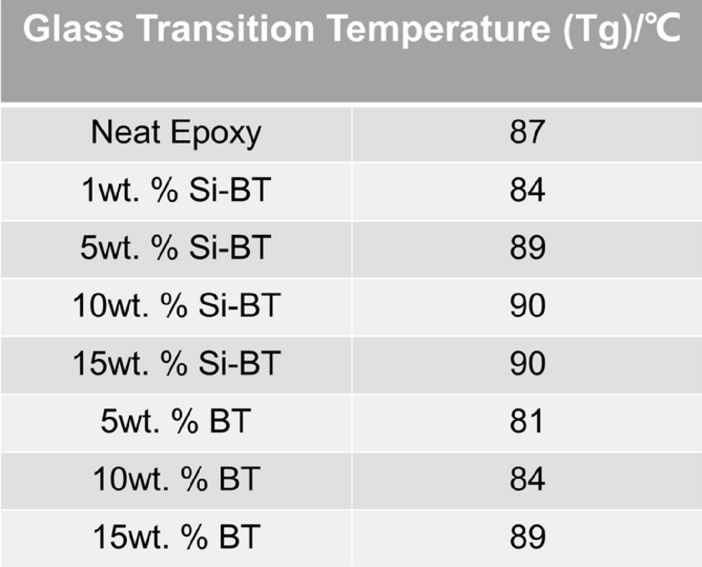
Glass transition temperatures of silane treated and untreated BaTiO_3_-Epoxy nanocomposites.

**Figure 17:**
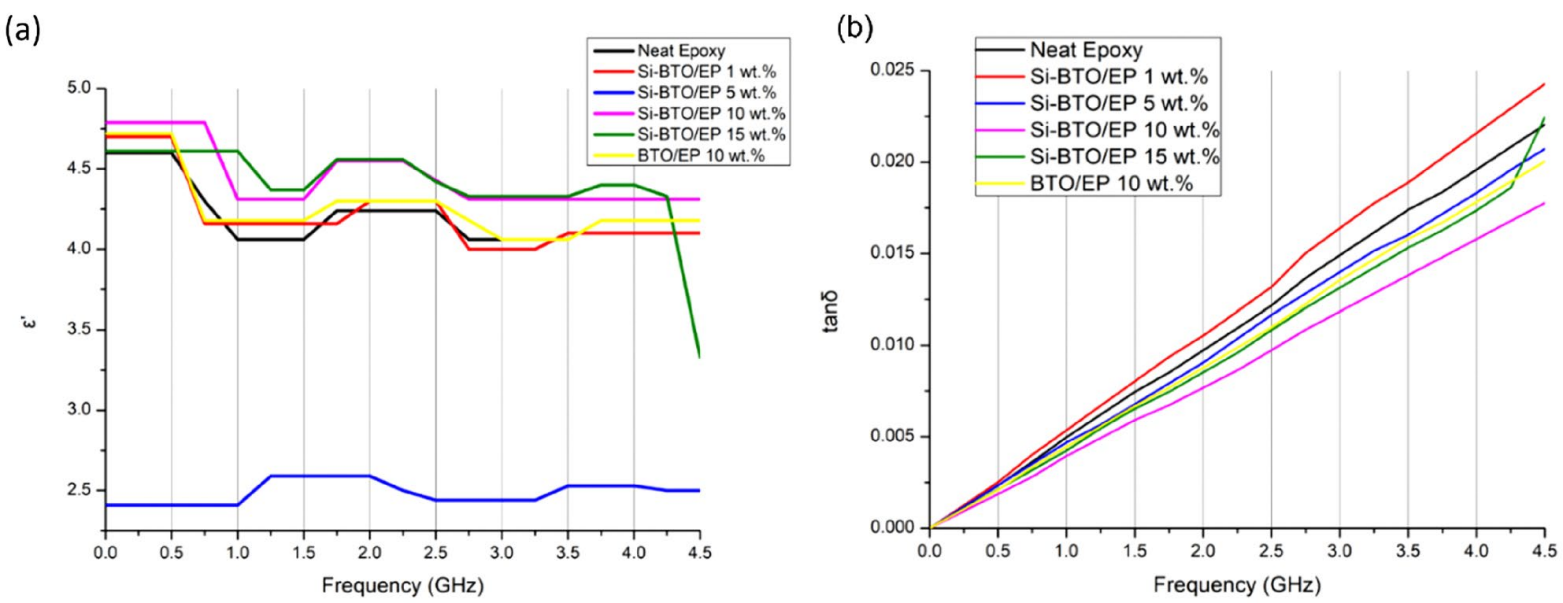
Dielectric measurements (**a**) real permittivity ${\varvec{\varepsilon}}\boldsymbol{^{\prime}}$, (**b**) loss tangent ${\varvec{t}}{\varvec{a}}{\varvec{n}}({\varvec{\delta}})$ from 0 to 4.5 GHz at room temperature of neat epoxy and BaTiO_3_-Epoxy nanocomposites at different weight loading.

Figure 15a presents the DSC spectra of a second heating scan for each epoxy nanocomposite with functionalised BaTiO_3_ after cooling down from the 220 °C previous runs. The glass temperatures per nanocomposite category have been identified in Fig. 16. It can be observed that epoxy nanocomposites are well cured without any perceptible exothermal peak. The red and black lines are drawn as a demonstration of ${T}_{g}$ variation along with the weight fraction of the nanofiller. The red line shows a gradual increase of ${T}_{g}$ with the increasing Si-BaTiO_3_ content. The ${T}_{g}$ has increased up to 90 °C compared with that of the neat epoxy, 87 °C, as shown in Fig. 17. The increased ${T}_{g}$ represents that more energy is needed from higher temperatures to complete the glass transition process, which indicates that the movement of polymer chains is inhibited by the functionalised BaTiO_3_ nanoparticles due to improved interfacial bonding. However, by adding the non-functionalised BaTiO_3_, the ${T}_{g}$ shows a remarkable drop at 5 wt.% at the beginning as marked by a black line in Fig. 15b. When the weight loading reaches 10 wt.%, the ${T}_{g}$ shows an increment surge from 81 to 84 °C. The ${T}_{g}$ of the nanocomposites reaches 89 °C at 15 wt.%. The untreated particles at 5 wt.% have a negative effect on the Tg by obstructing the formation of the cross-linking structure of epoxy due to particle agglomeration as shown previously in the SEM images. However, the addition of the nanoparticles can enhance the mobility of the polymer chain by introducing increased free volume at the filler-matrix interface^89^. For the uncured pure epoxy resin, the mobility of the polymer segments decreases and Tg increases with the temperature rise during the curing process. When the Tg finally approaches the curing temperature and becomes higher, the curing process ceases due to the lack of mobility of the segments. The free volume created by introducing more untreated nanoparticles increases the mobility of the segments during curing, and hence favours the curing process and higher Tg.

#### Dielectric properties of BaTiO_3_/epoxy composites

The microwave dielectric properties of the epoxy nanocomposites were investigated from 0.25 to 4.50 GHz at a 0.25 step increment using a VNA PNA-X N5245A via a stripline technique at room temperature. The real permittivity ${\varepsilon }^{^{\prime}}$ and dielectric loss tangent $\mathrm{tan}\delta$ calculated from Eq. (2) at room temperature are shown in Fig. 17.

The ${\varepsilon }^{^{\prime}}$ of all samples over the measured frequency range have slight reduction trends with increasing frequency due to the dipoles in the material that cannot follow the speed of the alternating field and reorient themselves with the field direction^89^. Compared with neat epoxy, the inclusion of Si-BaTiO_3_ nanoparticles at 10 wt.% and 15 wt.% give a perceptible rise to the value of ${\varepsilon }^{^{\prime}}$ through the frequency range, which are similar results as reported in other studies^90–92^. In the meantime, the 1 wt.% and non-treated 10 wt.% BaTiO_3_/Epoxy samples present similar values of ${\varepsilon }^{^{\prime}}$ with the neat epoxy due to little amount of BaTiO_3_ of 1 wt.% and aggregations of unfunctionalized 10 wt.%^92^. The silane treated samples have higher values in ${\varepsilon }^{^{\prime}}$ due to a more uniform dispersion of BaTiO_3_ nanoparticles^90,91,93^. However, there aren’t huge differences between the values of ${\varepsilon }^{^{\prime}}$ at 10 wt.% and 15 wt.%. As discussed formerly, the ferroelectricity character of tetragonal BaTiO_3_ enables a spontaneous polarisation that can be reversed by an applied field^94^. Therefore the ${\varepsilon }^{^{\prime}}$ of epoxy is enhanced due to the addition of BaTiO_3_ nanoparticles, which agrees with the findings from other researches^90,91,93,95^. Generally speaking, a substantial increase in the dielectric properties of modified epoxy is only achieved by a generous amount of BaTiO_3_ content as high as 90 wt.%^96^. The blue line that represents the dielectric response of 5 wt.% silane-treated BaTiO_3_ epoxy nanocomposites, exhibiting clearly a significantly lower value of real permittivity ${\varepsilon }^{^{\prime}}$ below the neat epoxy’s. This drastic decline in the value of ${\varepsilon }^{^{\prime}}$ is attributed to the testing errors from airgaps that were identified due to the uncertainties associated with handling the samples, between the sample and conductive line^97^ while was not observed in the other samples. Figure 17b illustrates that the loss tangent in all samples increases with increasing frequency. On the contrary with the real permittivity, the loss tangent has slightly decreased with adding BaTiO_3_ powders except from 1 wt.% content. The loss tangent of a composite system has contributions from dipole orientation, conduction loss and interfacial polarisation^98^. Silane treatment of the BaTiO_3_ powders introduce a reduction of the concentration of ionizable hydroxyl groups (-OH) on the BaTiO_3_ surface and hinders the mobility of the charge carriers on the surface. The lowered conduction loss could be the primary reason for lowered loss tangent^80^.

### In-situ strain response under microwave at different power levels

#### 100 W and 440 W microwave exposure power

**Figure 18:**
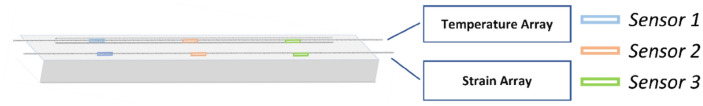
Schematic illustration of surface-bonded FBG arrays measuring in-situ temperature and strain.

**Figure 19:**
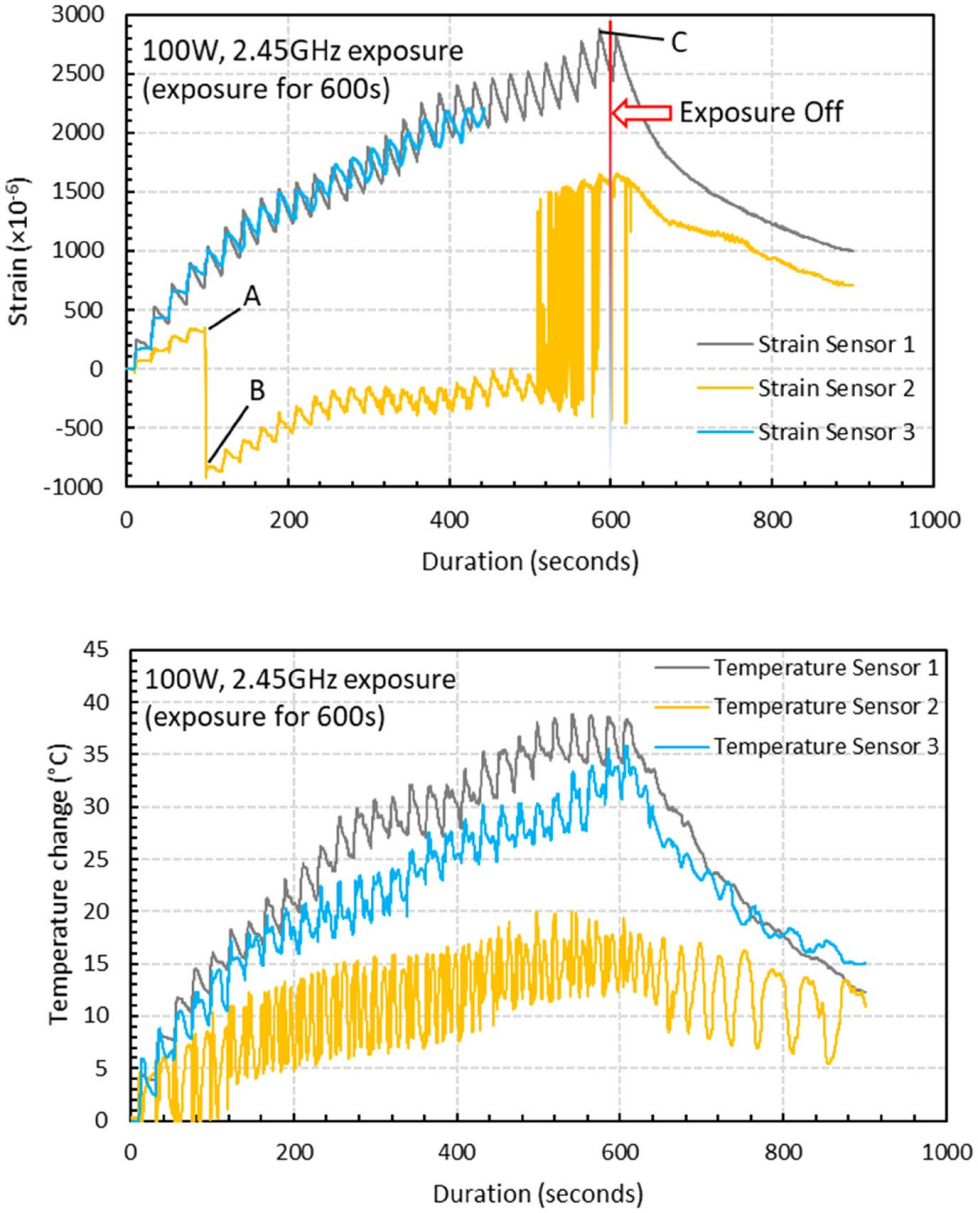
Strain and temperature change measurements of 15 wt.% silane-treated BaTiO_3_-Epoxy samples under 100 W for 600 s.

**Figure 20:**
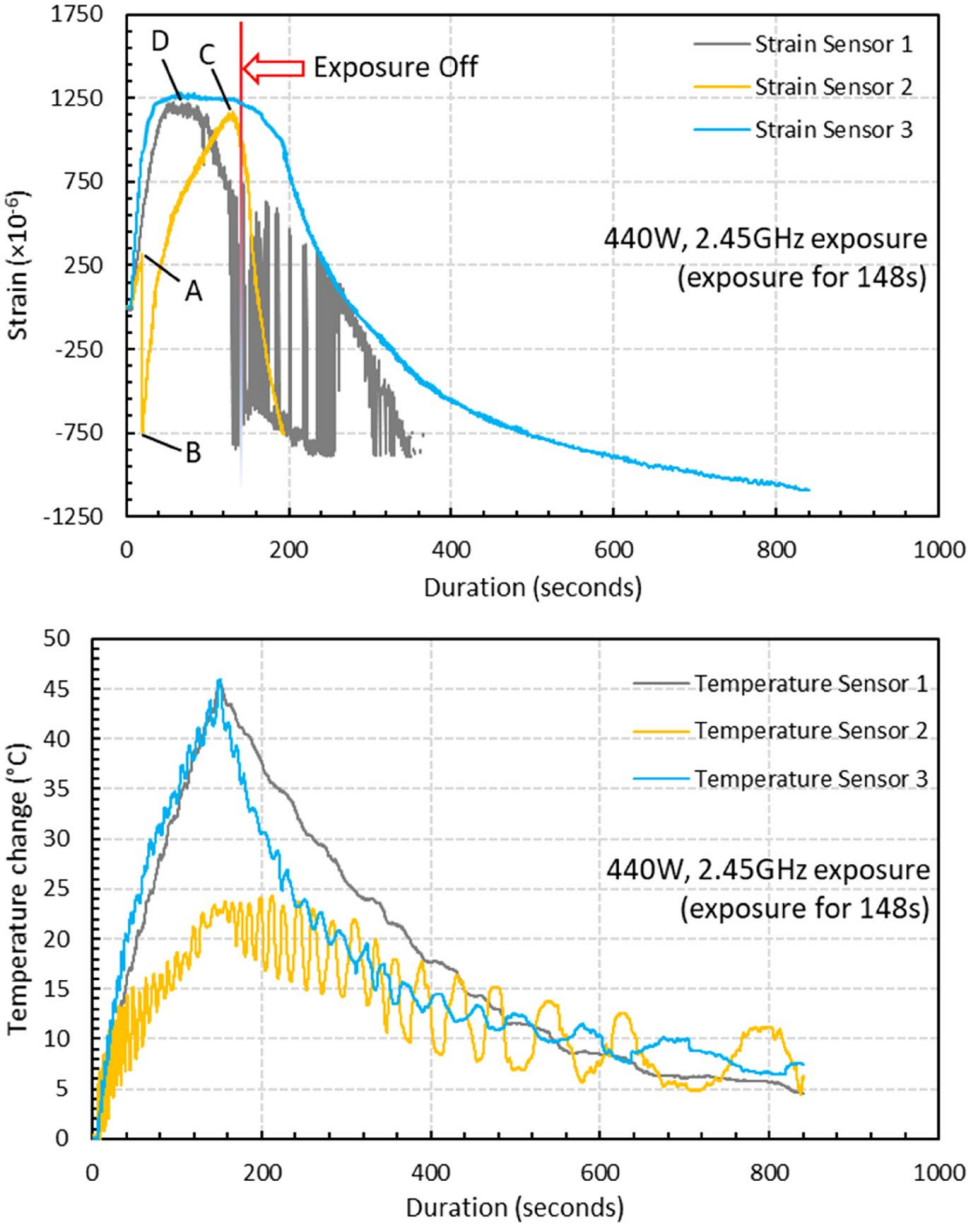
Strain and temperature change measurements of 15 wt.% silane-treated BaTiO_3_-Epoxy samples under 440 W for 148 s.

Labelling for strain and temperature FBG arrays is illustrated in Fig. 18. Field-induced strains in the nanocomposites with 15 wt.% BaTiO_3_ (the highest wt.% examined) has been investigated within a microwave exposure at 100 W and 440 W (Figs. 19 and 20, respectively) in the three specimens which exhibited nearly similar trends and magnitude (one data presented in the figures in the interests of clarity). The evolution of strain and temperature have been measured in situ by the two arrays; one array measures the strain while the other one measures the temperature. Sudden drastic fluctuations and absent data presented in FigS. 19 and 20 are due to Bragg wavelengths moving into adjacent spectral windows that were set up on the sensor interrogator.

**Figure 21:**
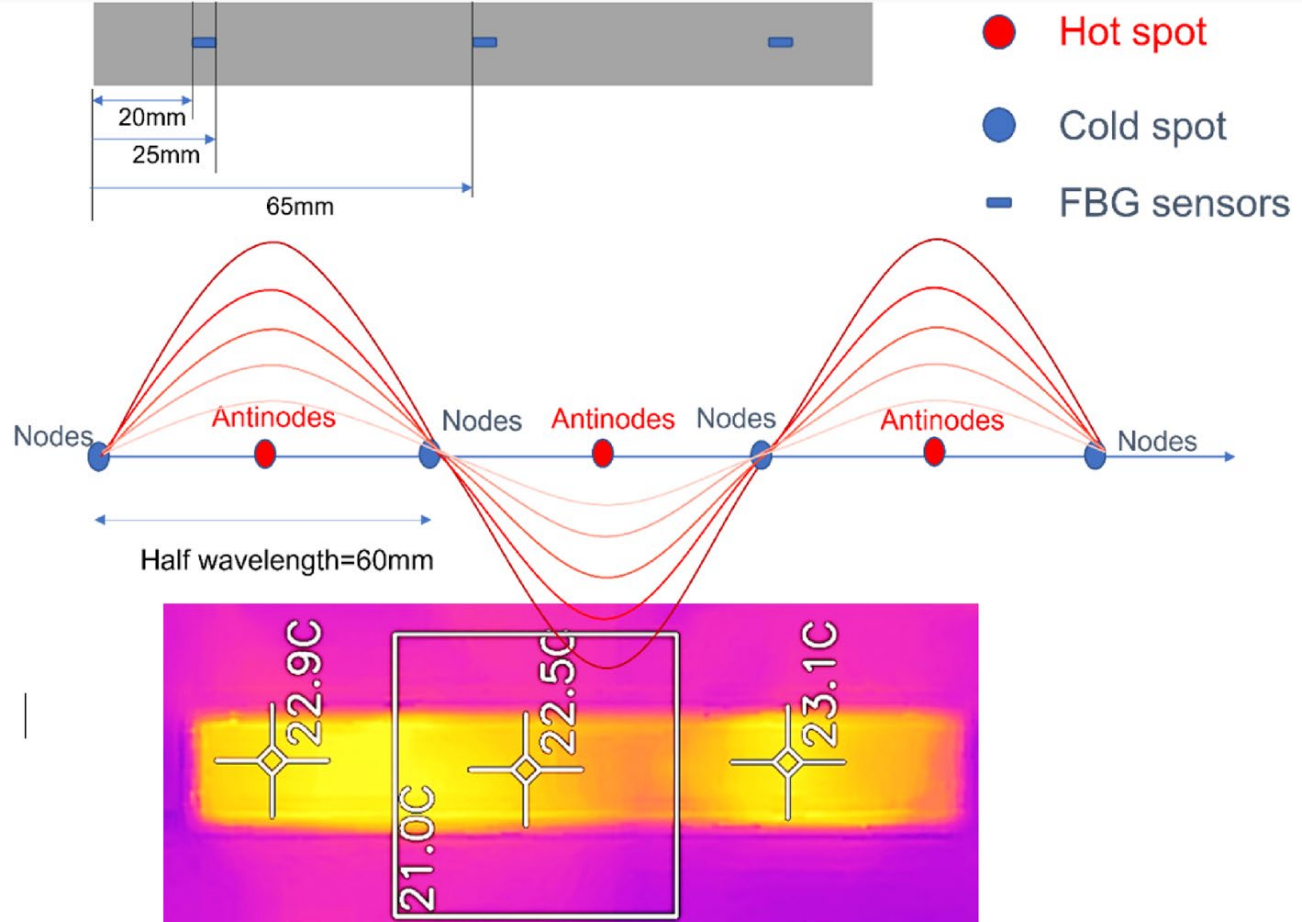
Schematic illustration of (above) the location of FBGs on the sa mple surface, and (below) standing waves formed in a microwave oven showing hot spots (antinodes) and cold spots (nodes).

The strain and temperature data exhibit a general increasing trend with the exposure. They drop gradually, immediately, after the microwave stopped. The temperature appeared to be higher at both ends of the specimen (sensor 1 and 3, located left and right respectively) compared to the middle (sensor 2), which is in accordance with the ‘hot spot’ theory due to microwave nonuniformity as illustrated in Fig. 21. Sensors 1 and 3 data also follows more similar temperature increasing trend (magnitude and rate). The microwave oven cavity is designed to be a multimode resonant cavity, similar to a closed waveguide. The microwaves generated by the magnetron are reflected back and forth by the metal walls inside the cavity and eventually forms standing waves throughout the cavity’s volume as illustrated in Fig. 21^99^. The illustration is accompanied by a thermal image captured from the sample under the microwave, showing the approximate locations of the nodes and anti-nodes. As seen, the anti-nodes (hot spots) are located at or surrounding the sensors. Under microwave exposure, the distance between the area with higher temperature (antinodes) and the cold spot with lower temperature (nodes) is a quarter of the wavelength, which is approximately 31 mm^100^. The sensors have been positioned equal distances apart, and accordingly, they fell in different temperature zones under the standing waves, identified schematically in the figure (assuming that the far-left edge of the sample represents a node).

**Figure 22:**
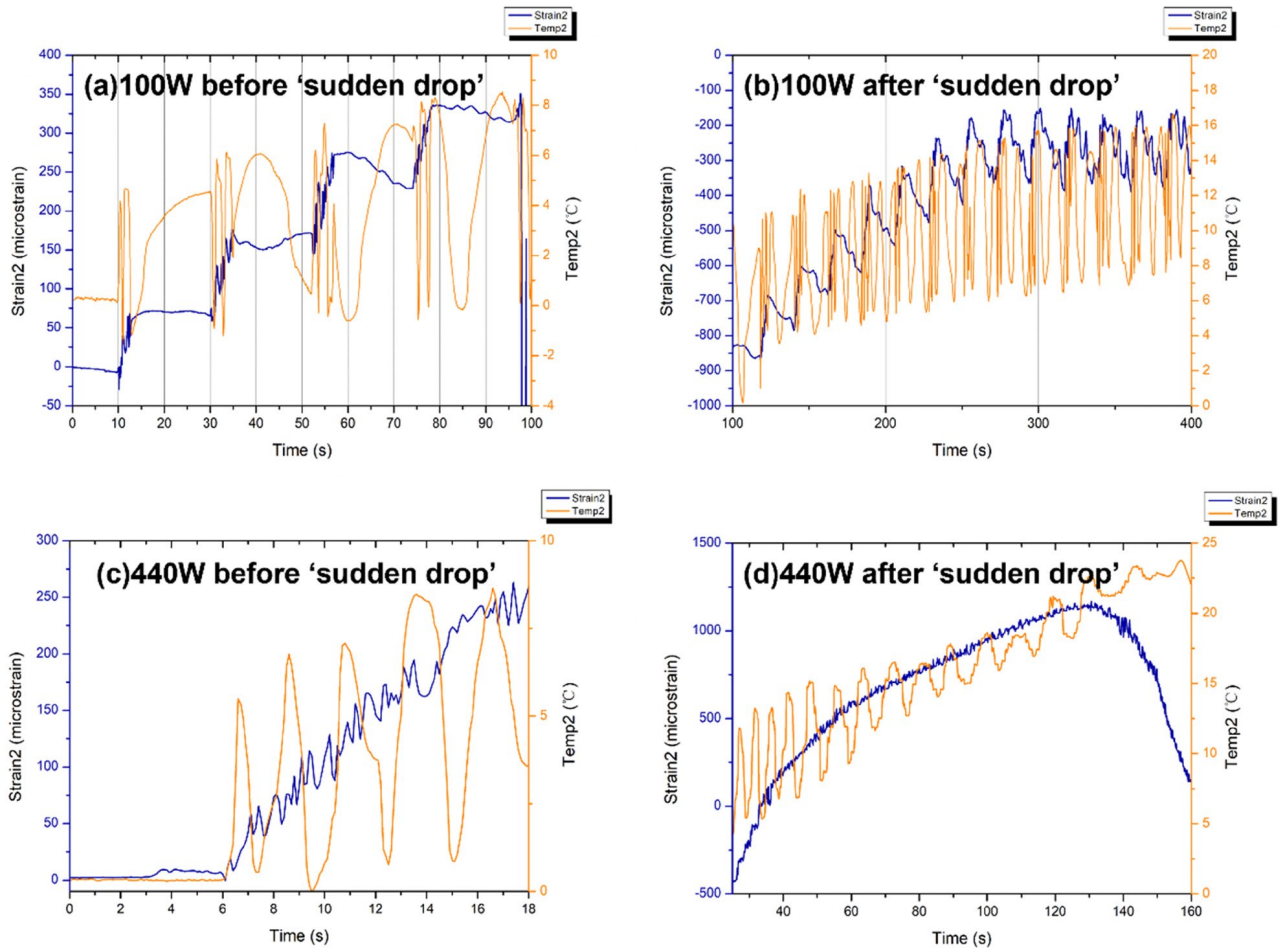
Strain and temperature variations measured by sensor 2 before and after the ‘sudden drop’ at 100 W (**a**, **b**) and 440 W (**c**, **d**).

The strain measured from a FBG sensor is derived from the change in the wavelength of reflected ultraviolet light due to grating period change. The change with strain and temperature is expressed by^101^:

69 $$\frac{\Delta \lambda }{{\lambda }_{0}}=k\cdot \varepsilon +{\alpha }_{\delta }\cdot \Delta T$$

where $\Delta \lambda$ is wavelength shift, ${\lambda }_{0}$ is the base wavelength, $k=1-p$, $p$ is the photo-elastic coefficient, $p$ =0.22, $\varepsilon$ is the strain due to both mechanical and thermal factors, $\Delta T$ is the temperature change, and ${\alpha }_{\delta }$ is the change of the refraction index. As expressed in Eq. (69), the measured data from the strain sensor are a combination of two factors. The first part is the actual strain of the material including any mechanical strain and strain due to temperature changes. The second part is the wavelength shift due to a change in the glass-refraction index of optical fibre with temperature. According to the FBG sensors manufacturer, 1 °C temperature variation induced strain of optical fibre itself is approximately equivalent to eight micro-strain. Under the 100 W and 440 W, the strain measured at the beginning of the microwave radiation in strain sensors 1 and 3 have similar trends of increasing as predicted when the temperature sensors 1 and 3 measurements have close gradients, having higher temperatures than that measured by sensor 2. A sudden drop in the strain measurements of the middle sensors (strain 2) can be observed in both cases soon after the initial surge, labelled as A-B. It instantaneously alters the strains by nearly identical 1162 and 1008 micro-strains (difference between A and B) under 100 W and 440 W, respectively. Such decline in the strain measurements is attributed to an immediate development of a compressive strain in response to the microwave exposure, as hypothesised in Sect. 2. Note that sensor 2 temperature is the lowest amongst the three sensors, in which the thermally induced strain is negligible. Such phenomenon could not be associated with the temperature rise since the temperature change is approximately 12 °C and 5.7 °C from room temperature 19 °C when the sudden drop occurs at point A, which is remarkably lower than 170 °C when post-curing shrinkage is introduced as indicated by the exothermic peak in the DSC spectra in Fig. 14, and lower than the ${T}_{g}$ to have any detrimental effect. Moreover, it is observed that unloading the specimen from the 440 W exposure results in a residual compressive strain in all sensors’ locations. This occurs after the high non-linear variation of strains in the case of 440 W, unlike the linear strain behaviour under 100 W. This is analogous to mechanical field introduction in which unloading an elastic–plastic specimen beyond its elastic regime under tensile loading may introduce a compressive residual strain distribution depending on the hardening behaviour (i.e. kinematic or isotropic). Further investigation was conducted on the phenomena observed, described as follows:

Different FBG arrays have been adhesively bonded at the same location on the surface of two specimens made of 15 wt.% with identical geometric dimensions and the location of the two specimens are placed at the exact location inside the microwave cavity. Accordingly, the distribution of microwaves in both cases is considered to be identical, also verified using thermal camera. The strain and temperature change measurements of sensor 2 under 100 W and 440 W before and after the ‘sudden drop’ is presented in Fig. 22a–d. It can be observed from Fig. 22a,b that the measured strains demonstrated a steady step-wise increase with the increasing temperature that fluctuated within a constant range of approximately 8 °C. The fluctuations in temperature change exhibit a periodic pattern that is approximately in phase with every step of the increment in strain. Under 440 W as shown in Fig. 22c,d, the increase in the strain and temperature data follow a similar trend as that under 100 W but at a higher rate.

**Figure 23:**
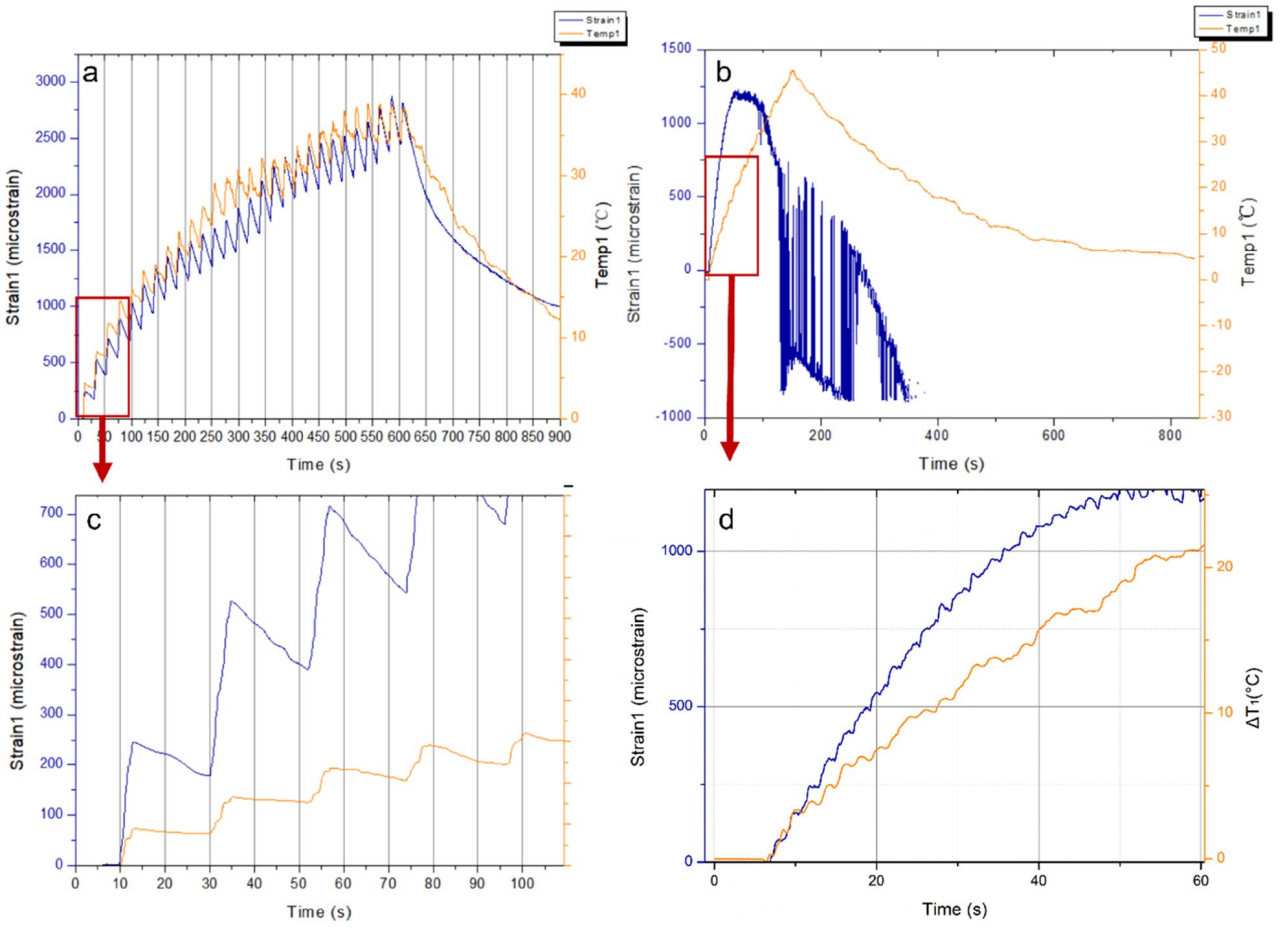
Strain and temperature measurements of FBG sensor 1 of (**a**) and (**c**) 15 wt.% BaTiO_3_/Epoxy nanocomposite sample under 100 W for 600 s, (**b**) and (**d**) 15 wt.% BaTiO_3_-Epoxy nanocomposite sample under 440 W for 148 s.

In contrast with the middle sensors, the strain and temperature increments from sensor 1 under 100 W and 440 W are predominately synchronized as presented in Fig. 23. It could be observed from Fig. 23a,c that the cycle time for each step is approximate 20 s and each step is approximately 100 micro-strain. A similar trend is also observed in the first 50 s of the microwave exposure under 440 W from Fig. 23b. However, the cycle time for each step shown in Fig. 23d falls sharply to 2 s accompanying a higher increment of 300 micro-strain compared with the 100 W data which are attributed to the increased heating rate at higher microwave power.

*Adhesive FBG data:*

Despite the widely known utilisation of FBG strain sensors, there exists a layer of adhesive and protective coating that may affect the data from the nanocomposite^102^. The FBG sensors are adhesively bonded to the surface of specimens by PERMABOND 920 Cyanoacrylate. The dielectric and thermal properties of the adhesive and neat epoxy are presented in Table 2.

**Table 2:** Dielectric properties and coefficient of thermal expansion of PTFE, Adhesive and neat epoxy.

	Real permittivity	Loss tangent	Coefficient of linear thermal expansion (CTE) microstrain/°C
PTFE	2.1^103^	0.0004^103^	124^104^
Adhesive	2.5^105^	< 0.02^105^	90^105^
Epoxy	4.25	0.01	75^106^

**Figure 24:**
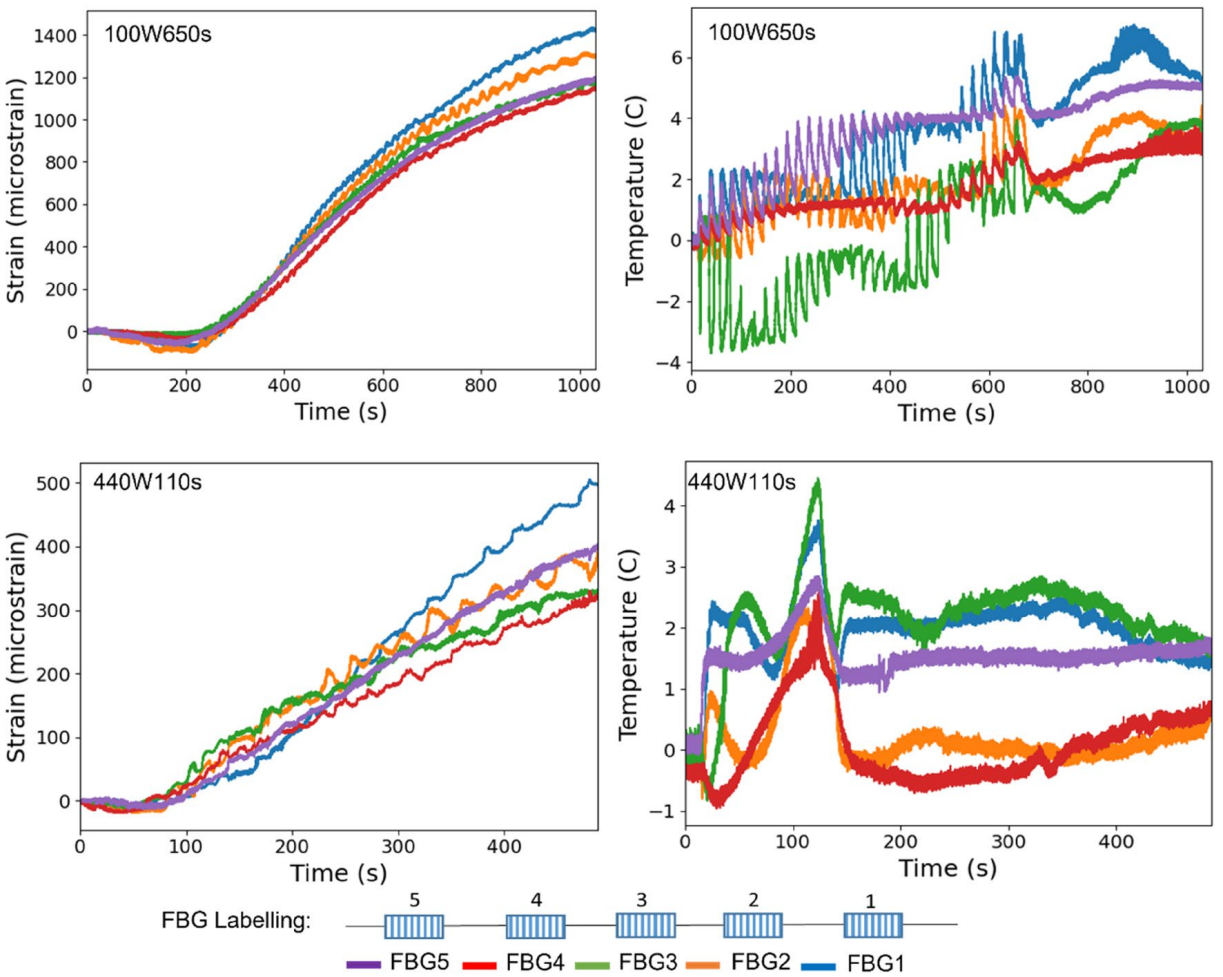
Strain and Temperature evolution measurements via FBG sensors in adhesive (Permabond 50) bonded on the surface of the PTFE holder under 100 W for 650 s and 440 W for 110 s.

Five FBG sensor arrays, 20 mm apart, were utilised to quantify the adhesive’s response distribution. Figure 24 presents the strain and temperature change measured via sensors bonded to a PTFE block with adhesive under 100 W and 440 W. It can be observed from Fig. 24 that temperature increases in the adhesive with microwave exposure time at both power levels as predicted while the temperature rise has a higher rate under 400 W. The maximum temperature changes are around 7 °C and 4.5 °C for 100 W and 440 W, respectively. The insignificant amount of temperature rise is the direct evidence of relative less interaction of adhesive and microwave. The strain values steadily increase with the temperature rise in both cases due to the thermal expansion of PTFE and adhesive. The calculated thermal strain of PTFE based on Fig. 24 and Table 2 under 100 W is approx. 868 micro-strain at 7 °C temperature change. The calculated value is almost consistent with the measured value of strain sensor 1 which is approx. 960 micro-strain at similar temperature variation range. Unlike 100 W, the measured strain of sensor 1 in 440 W shown as the blue line (when microwaves are removed) is approximately 10 micro-strain, which is much lower than 458 microstrain, the calculated thermal strain of PTFE at the same temperature change (3.7 °C).

At the microwave exposure, the adhesive solely interacts with the microwave, and is heated up owing to its higher dielectric loss compared with the PTFE. However, the PTFE have a higher CTE than the adhesive. Consequently, the measured strain under 100 W is mainly attributed to the thermal expansion of PTFE from the conduction of heat. The strain sensors under 100 W showed strain increase initiated after 200 s, indicating that a substantial amount of time is required to trigger the heating conduction between the adhesive and PTFE. On the contrary, 440 W has a higher rate of temperature rise within 110 s exposure. Based on the analysis of the calculated and measured strain, the strain under 100 W is mainly attributed to PTFE thermal expansion while the strain under 440 W is dominated by the thermal expansion of adhesive. Under 440 W, a similar trend in temperature rise is noted between sensors 1 and 3, as well as sensor 2 and 4, which is described by the ‘hot spot’ theory due to non-uniform microwave field.

*Neat Epoxy FBG data:*

**Figure 25:**
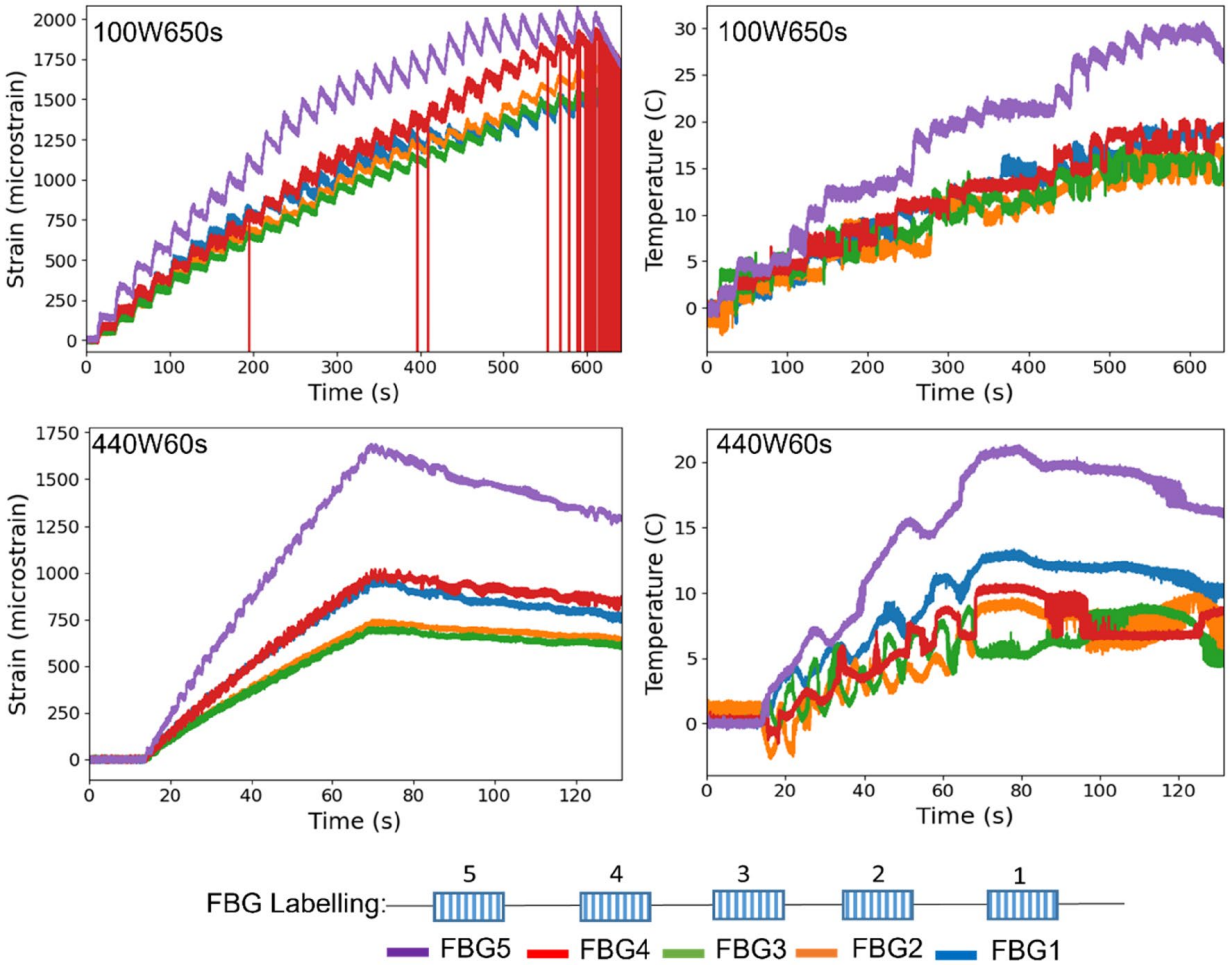
Strain and temperature evolution of the neat epoxy sample under 100 W and 440 W.

**Figure 26:**
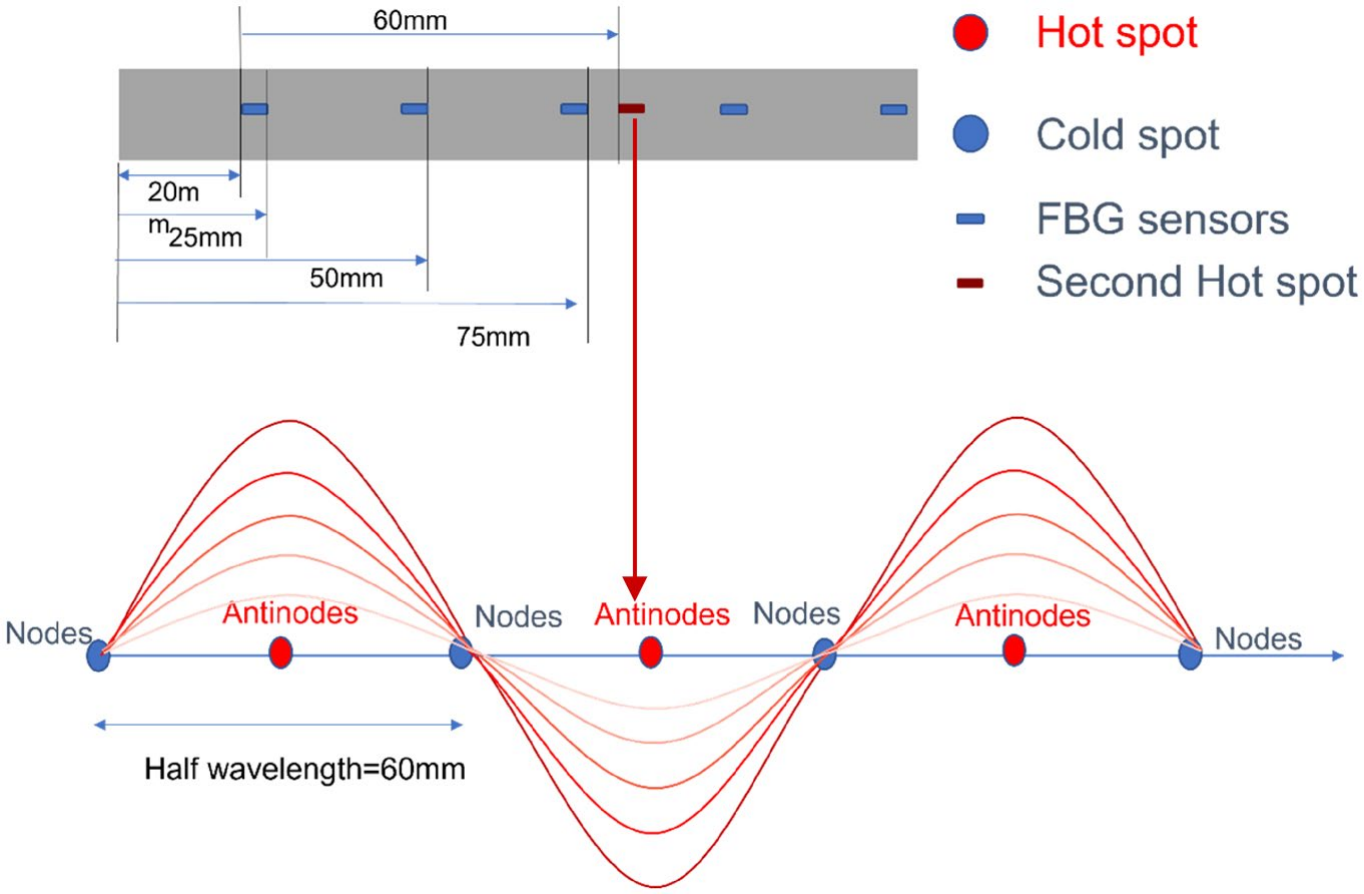
Schematic illustration of the location of FBG sensors on the neat epoxy surface, 20 mm apart, and standing waves formed in a microwave oven showing hot spots (antinodes) and cold spots (nodes).

Strain and temperature evolution of the neat epoxy with adhesively bonded arrays together under microwave are presented in Fig. 25. The microwave running time has been estimated to maintain the temperature well below the ${{\varvec{T}}}_{{\varvec{g}}}$ of the BaTiO_3_-epoxy nanocomposite. The temperature increases as a function of the exposure duration (equivalently, microwave energy). Sensor 5 data present a higher rate of temperature variation compared with the other sensors conforming to the ‘hot spot’ theory. The region in which sensor 5 is located within the zone with a higher rate of temperature rise that is denoted as a ‘hot spot’ zone presented in Fig. 26, hence the next hot spot shown in Fig. 26 (red square symbol) is half (60 mm) of the microwave wavelength (120 mm) is located between sensors 3 and 2. As schematically demonstrated, the rest of the FBG sensors are located out of the hot spot region, and therefore they develop similar rates of temperature under microwave.

Strain and temperature response of the neat epoxy with bonded sensor arrays is investigated under microwave (Fig. 25). The temperature increases as a function of microwave exposure time and starts to drop as soon as the microwave stops as predicted. No sudden drop such as that observed in the nanocomposite is observed for the neat epoxy which indicates the phenomenon linked with the embedded BaTiO_3_. Sensor 5 presents a higher rate of temperature change compared with the other sensors, corresponding to the ‘hot spot’ theory discussed formerly, and illustrated in Fig. 26. As schematically shown in Fig. 26, the other FBG sensors are located out of the hot spot region, and therefore develop similar rates of temperature variation under microwave.

The temperature change in both cases reached the highest value of 30 °C compared with that measured for the adhesive only. The measured strain follows a increasing trend with the temperature change, which is a resultant of the thermal expansion from the adhesive and neat epoxy. Maximum strain and temperature values measured by left-side sensor for adhesive on PTFE (sensor 5), adhesive on epoxy (sensor 5), and adhesive on nanocomposite (sensor 1) are tabulated in Table 3, summarised from the data shown in Figs. 19 and 20, under 100 W and 440 W exposure.

**Table 3:** Strain and temperature change measurements under 100 W and 440 W of three categories: FBG sensors bonded by adhesive to PTFE, FBG sensors bonded by adhesive to the neat epoxy, and FBG sensors bonded by adhesive to the epoxy nanocomposite with BaTiO_3_.

100 W			440 W		
Strain/*10^−6^	ΔT/°C	Time/s	Strain/*10^−6^	ΔT/°C	Time/s
*Adhesive + PTFE*					
790	5.0	650	70	2.8	110
Adhesive + epoxy					
375	5.0	40	320	5.0	14
2080	30.0	600	1700	21.0	60
*BaTiO*_*3*_*-epoxy nanocomposite + Adhesive*					
243	5.0	31	330	5.0	15
2230	30.0	445	1225	21.0	58

The measured strain of the first two categories (adhesive on PTFE and adhesive on the neat epoxy) are temperature compensated which means the effect of thermal expansion from the optical fibre itself is eliminated. Approximately 8 micro-strain is achieved by a 1 °C change in temperature according to the manufacture. The true strain of the epoxy nanocomposites samples is then lower than the measured strain from sensor 1:

For the case of 100 W exposure, the measured strain of adhesive on PTFE is mainly attributed to the thermal expansion of the PTFE, as formerly discussed. When the FBG arrays are adhesively bonded to a neat epoxy sample, the measured strain drops by approx. 52% compared to that (790 micro-strain) from the sensor adhesively bonded on PTFE at the same temperature variation of 5 °C. It is due to the lower CTEs of epoxy and adhesive compared to that of the PTFE. Furthermore, at this temperature change (5 °C), the measured strain without temperature compensation of epoxy nanocomposites loaded with BaTiO_3_ drops 35% compared with the neat epoxy. However, as temperature change rises to 30 °C, the measured strain in both cases tends to be similar. Under 440 W, the value of strain is mostly dominated by adhesive as described earlier. When the temperature change firstly reaches 5 °C at the beginning of the microwave exposure, the neat epoxy and nanocomposites have similar strain (320 and 330 micro-strain, respectively).

### Raman characterisation

Raman spectroscopy was utilised to examine any residual/remnant strain induced by the particles to the surrounding epoxy matrix, or any crystal structure transformation of BaTiO_3_ particles that might be introduced by the microwave exposure. The middle region of the samples where the ‘sudden drop’ in strain occurred was of particular interest, hence it was the region where the laser was focused to obtain the Raman spectra. Another run at microwave power of 1000 W for 50 s was performed for such effect.

**Figure 27:**
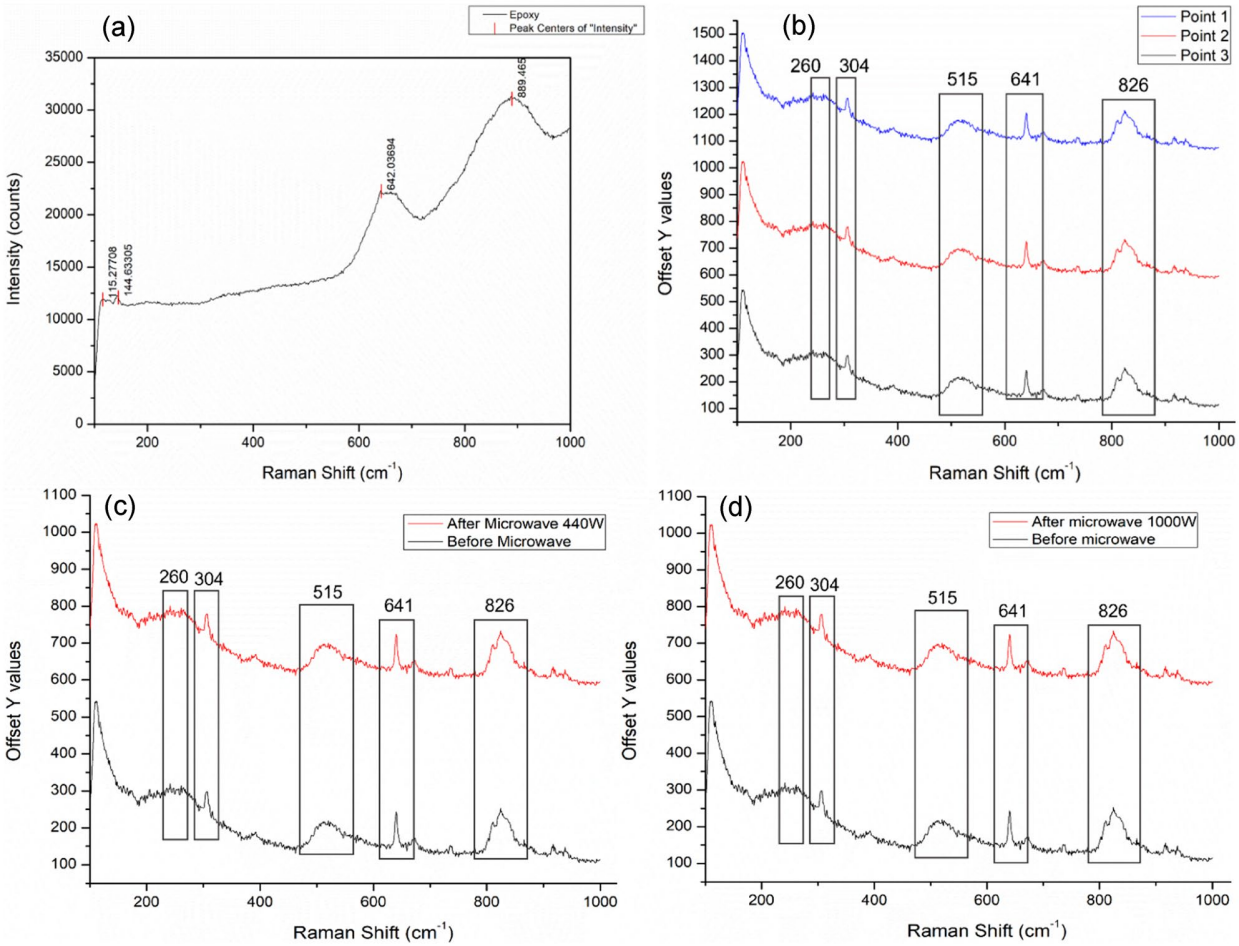
Raman spectra of (**a**) neat epoxy, (**b**) three random points of 15 wt.% epoxy nanocomposites with Si-BaTiO_3_ before microwave exposure, (**c**) 15 wt.% epoxy nanocomposites with Si-BaTiO_3_ before and after microwave exposure of 440 W for 150 s (**d**) 15 wt.% epoxy nanocomposites with Si-BaTiO_3_ before and after microwave exposure of 1000 W for 60 s.

Raman characterisations have initially been performed on the neat epoxy and BaTiO_3_-epoxy nanocomposite samples with 15 wt.% prior to microwave to establish baseline curves for identification of possible change in the position of Raman peaks for the samples post microwave exposure. Raman spectra of the epoxy are shown in Fig. 27a. Broader peaks at 600–800 cm^−1^ and 800–1000 cm^−1^ are presented in Fig. 27a for the neat epoxy indicating an amorphous structure. The Raman spectra on three random points within the region of interest of the 15 wt.% Si-BaTiO_3_ epoxy nanocomposite (Fig. 27b) present a good consistency with 260 cm^−1^ and 515 cm^−1^ as characteristic peaks of BaTiO_3_**,** and 304 cm^−1^ indicating the tetragonal phase of BaTiO_3_ representing the out-of-phase and axial motion of the two oxygen atoms facing oppositely^107,108^. The stretching mode of Si–O from silane functionalisation on the particle surface is presented as peaks at 641 cm^−1^ and 826 cm^−1^^109^. Raman spectra of the samples before and after microwave exposure at 440 W at room temperature are shown in Fig. 27c. Another run at 1000 W for 50 s was performed with Raman performed at the same location as that at 440 W, presented in Fig. 27d. No conspicuous differences are observed, and it could be noticed that the 304 cm^−1^ still emerges without recognizable changing in peak height, which implies no changes in the crystal structure of BaTiO_3_ after the microwave exposure, and no or slight remnant strain field.

## Discussion

### Compressive strain field induced in BaTiO_3_-epoxy nanocomposite under microwave exposure

**Figure 28:**
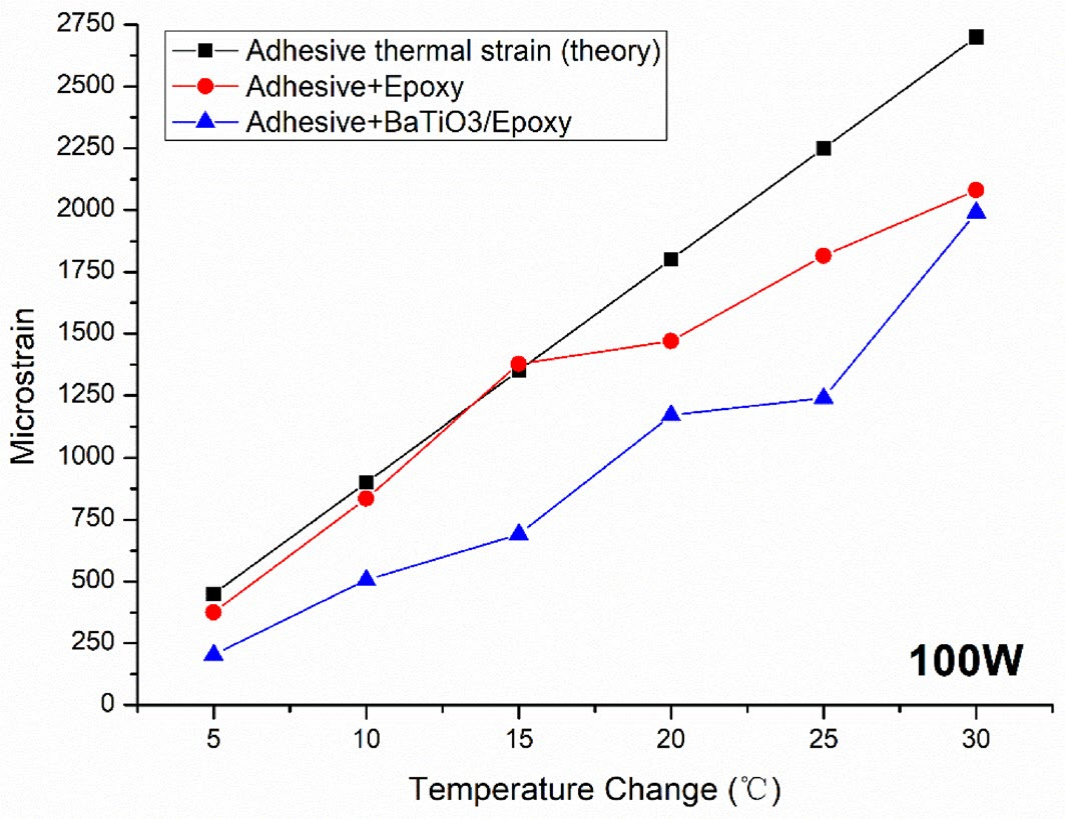
Evolution of micro-strain with temperature rise—theoretically calculated thermal expansion of adhesive (black), actual strain data from sensor 5 of adhesive bonded onto neat epoxy (red), and actual strain data from sensor 1 of adhesive bonded to 15 wt.% BaTiO_3_-epoxy nanocomposite (blue) under 100 W at 5 °C, 10 °C, 15 °C, 20 °C, 25 °C, and 30 °C.

**Figure 29:**
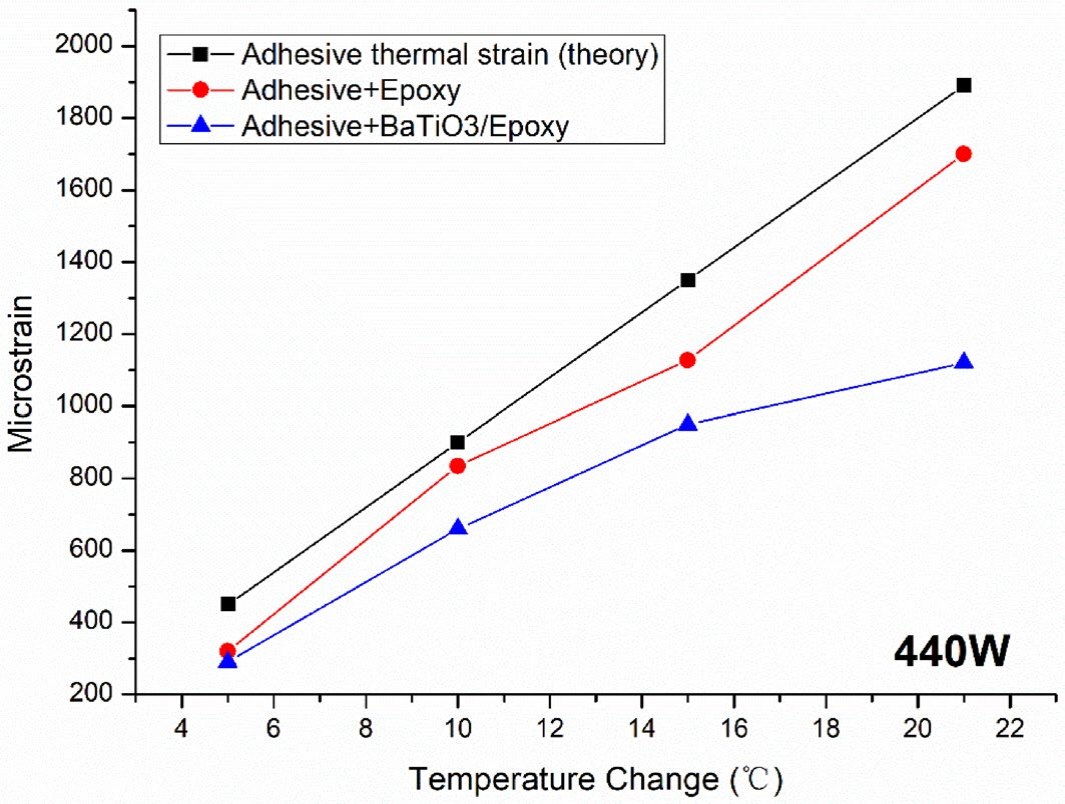
Evolution of micro-strain with temperature rise—theoretically calculated thermal expansion of adhesive (black), actual strain data from sensor 5 of adhesive bonded onto neat epoxy (red), and actual strain data from sensor 1 of adhesive bonded to 15 wt.% BaTiO_3_-epoxy nanocomposite (blue) under 440 W at 5 °C, 10 °C, 15 °C, and 21 °C.

It was observed from the FBG test results that the overall strain measurements of the 15 wt.% BaTiO_3_-epoxy nanocomposites are relatively smaller compared to those of the neat epoxy under 100 W and 440 W. The strain measurements of the nanocomposites from the beginning of the microwave exposure (ΔT = 5 °C) to a later stage (100 W: ΔT = 30 °C 440 W: ΔT = 21 °C) from sensor 1 data are presented as blue triangle marks in Figs. 28 and 29. The strain measurements of the nanocomposites shown in Figs. 19, 20, and Table 3 have not been corrected to account for the temperature effect of fibre refraction index, while the results presented as blue triangle symbols in Figs. 28 and 29 are corrected values accounting for such effect. Strain measurements of the neat epoxy are also presented as red circle symbols to evaluate the mechanical strain induced by dielectric heating under the exposure.

It could be observed from the figures that the measured strain of the nanocomposite (blue triangles) are generally smaller than that of the neat epoxy (red circles). Notably, the difference increases with increasing temperatures under 440 W.

**Figure 30:**
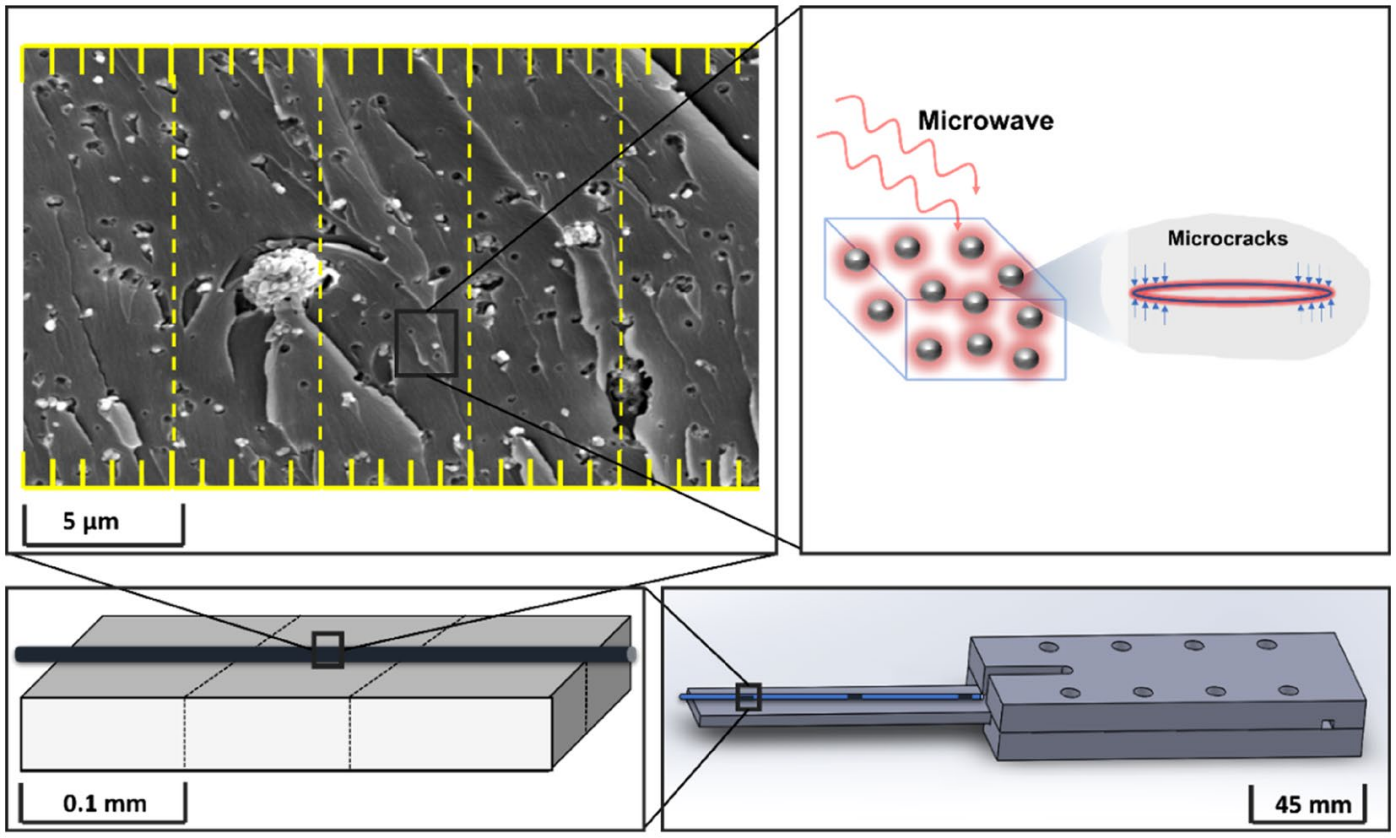
Schematic of the 15 wt.% BaTiO_3_-epoxy nanocomposite under exposure at different scales.

It is well known that the mechanical properties such as elastic modulus, Poisson's ratio, and shear modulus^110–112^ and the CTE^113–116^ are strongly dependent upon the microcracks in the matrix. Camahort et al*.*^117^ correlated a reduction of the CTEs of thermal cycled composite laminates in the specimens with microcracks identified under the microscope. A theoretical study of the CTEs in cracked laminates demonstrated that CTEs change as a function of the internal stress field^115^. This discrepancy in CTEs under different internal stress fields is due to the fact that the internal stresses induced by thermal cycling could be either tensile or compressive. When the stress field is tensile, the microcracks further opened up and cause a reduction in the value of CTE. Therefore, this correlation between the microcracks and CTEs might provide an explanation of the reduced strain measurements presented previously. Under microwave, domain wall movements are triggered in the BaTiO_3_ nanoparticles, hence stress fields around the microcracks might be changed and lead to a different CTE of the nanocomposite as illustrated in Fig. 30.

It is found by many studies that the CTE of composite material is highly dependent upon its constituents^118,119^. A decrease in the CTE of the nanocomposite material is observed and is attributed to a relative lower CTE of the fillers compared with that of the epoxy matrix [Dittanet, P., The use of nanosilica in epoxy resins, in Polymer Science and Engineering. 2008, Lehigh University]. The CTE of BaTiO_3_ and Epoxy is 1 microstrain/°C and 75 microstrain/°C [Khaleque, T., et al., Tailoring of Thermo-Mechanical Properties of Hybrid Composite-Metal Bonded Joints. Polymers, 2021. 13(2)]. The curing of BTO/Epoxy samples starts simultaneously with the temperature rising due to the microwave heating effect. However, under relatively lower temperatures, the curing effect is neglectable (exothermic peak can be seen from the DSC graph as curing is an exothermic reaction). The temperature of microwave heating is controlled below 50 °C which is below the exothermic peak around 170 °C. Therefore, the curing shrinkage effect is not significant during microwave radiation.

**Figure 31:**
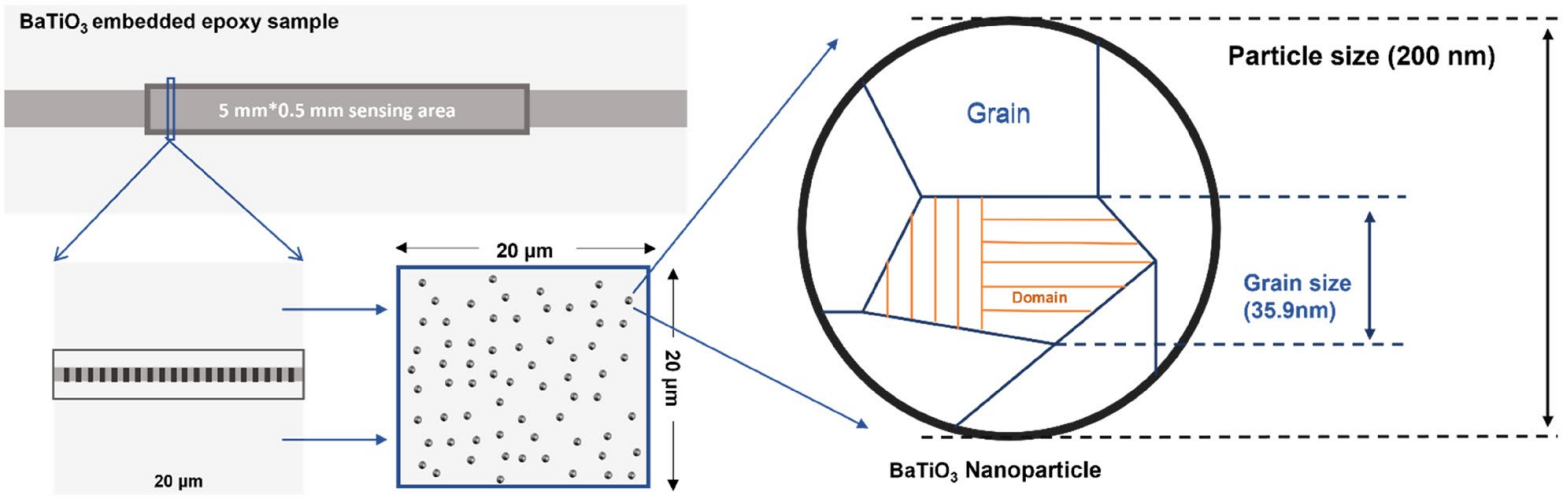
Nanocrystalline structure of BaTiO_3_ nanoparticles well-dispersed within the area over which the extrinsic strains are measured by the FBG sensors.

The average crystallite size of the purchased BaTiO_3_ nanoparticles are determined by XRD results using Scherrer’s equation^120^:

70 $${\text{Crystallite size}} = \frac{k\lambda }{{B\left( {2\theta } \right){\text{cos}}\left( \theta \right)}}$$

where $k$ is the dimensionless shape factor of the crystallite, $\lambda$ is the X-ray wavelength, $2\theta$ is the peak position, $B\left(2\theta \right)$ is the half-width of the maximum intensity of the peak and $\theta$ is the Bragg diffraction angle. A round-up value of 1.0 is selected in this study. The mean crystallite size is 35.9 nm, and it indicates that the corresponding grain size is 35.9 nm in the 200 nm BaTiO_3_ nanoparticles as shown in Fig. 31. Note that the strain measured by the sensors is averaged over a 5 mm × 0.5 mm region (i.e. sensor size). Such sensing area covers approximately 890,000 200 nm-particles, or alternatively 70 particles over a 20 × 20 µm^2^ area.

**Figure 32:**
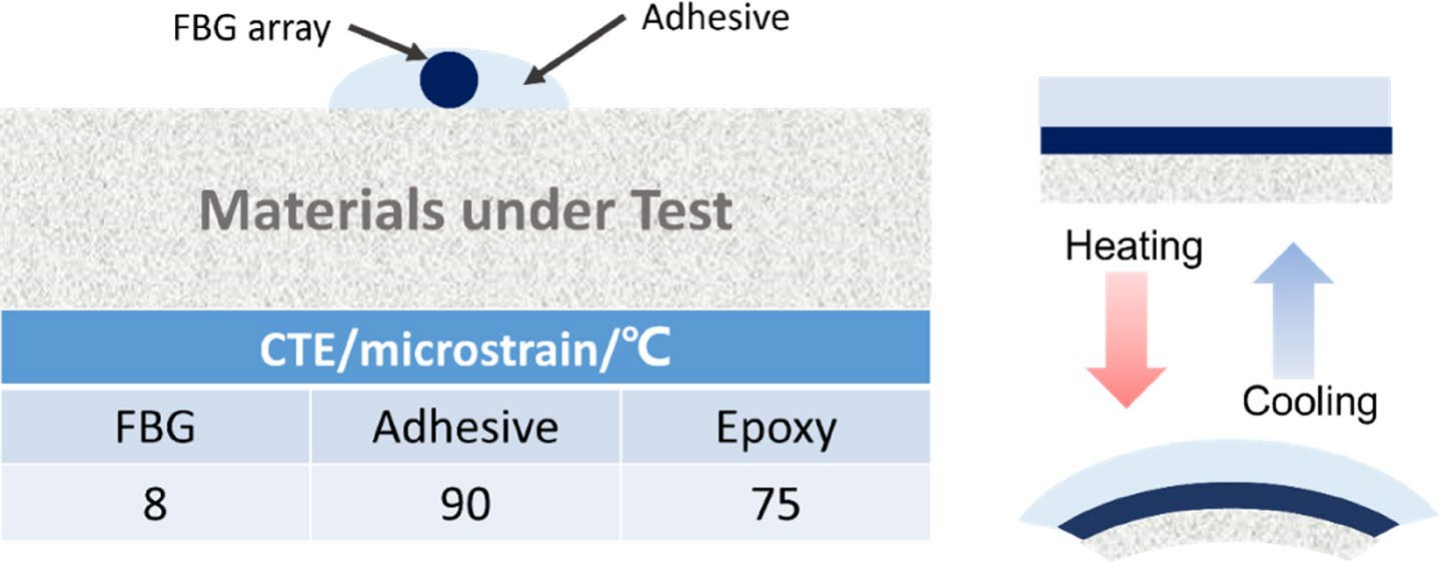
Schematic diagram of adhesively bonded FBG arrays to the sample’s surface and non-uniform thermal expansion due to mismatch in CTEs.

The black square marks in Figs. 28 and 29 represent the theoretically calculated values of linear thermal expansion of the adhesive used for bonding the FBG arrays. It can be observed that the strains in the neat epoxy are slightly lower than those measured by the thermal expansion of the adhesive at an identical temperature. A schematic diagram of the cross-section of an FBG array bonded to the sample is presented in Fig. 32. As discussed for Figs. 24 and 25, the adhesive exhibits lower interaction with microwave, hence a poorer dielectric heating effect. Thus, when the exposure initiated, the neat epoxy is firstly heated up due to the microwave selective heating based upon its different dielectric loss. The energy is then transferred to the adhesive via heating conduction from the epoxy, which initiates a thermal expansion in the adhesive. However, the epoxy possesses a relatively lower CTE in comparison with that of the adhesive. Consequently, the thermal expanding rates are different due to the mismatch in CTEs. As such, the measured strain in the FBGs is a result of thermal expansion of the adhesive constrained (via bonding) by the epoxy, causing the FBG arrays to bend with increasing temperature and bend back when the temperature drops. This behaviour might also be an explanation of the ‘zig-zag’ step-wise increment observed in Fig. 23. When the temperature ramping rate is slower under 100 W, the heat transfer between the epoxy and the adhesive is steadier compared with that under 440 W. Subsequently, the strain measurements exhibit a clearer step-wise increment under 100 W.

It is also noteworthy that the unanticipated finding of a ‘sudden drop’ during real-time strain measurement of the middle FBG sensor (presented in Figs. 19 and 20) occurs at the beginning of the microwave exposure at 1045 micro-strain under 100 W and 1261 micro-strain under 440 W, which is a clear implication of a phenomenologically induced compressive strain in the multi-material system around the middle FBG sensor. The true reason for the occurrence of this phenomenon remains unclear, however since the temperature effects are excluded and the phenomenon is only seen in the nanocomposite (and not in the neat epoxy), such high extrinsic strain variation is strongly attributed to the collective domain wall movements due to the reorientation of dipoles at high frequency in each domain.

### Effect of non-uniformity of the microwave cavity

As the microwave travels through the material, the microwave power decays due to the absorption by the material as it penetrates. As a result, the distribution of the microwave field within the material is non-uniform. For the 15 wt.% BaTiO_3_-epoxy nanocomposites, the penetration depth calculated by Eq. (61) is approximately 0.86 m, based on the measured dielectric properties at 2.5 GHz. This value indicates that approximately 63% of the energy from the incident microwave is dissipated at this distance under the sample surface. Therefore, for a nanocomposite with 3-mm thickness, it would be a reasonable assumption to make that the microwave fully penetrates the sample. Apart from the non-uniformity caused by the penetration depth of the microwave, there is also another factor that affects the non-uniform electric field distribution in the material, which is the standing waves formed in the microwave oven, extensively studied^121–123^. When the microwave field transmits and reflects within the cavity of the microwave oven, the standing waves form, and the ‘hot spot’ phenomenon manifests. The distance between two ‘hot spots’ is half of the wavelength of the incident microwave, which is approximately 60 mm. Therefore, the temperature change measured by FBG sensors exhibit different heating rates along with the 130 mm sample.

### Effect of absorbed microwave power

The power absorbed by the material is evaluated by the method of the energy balance of the microwave heating effect. At power 100 W, the average power per unit area in the 354 × 338 mm^2^ microwave oven is calculated as follows:

71 $$I=\frac{P}{A}=\frac{100}{0.354*0.338}\approx 835.8\mathrm{ W}/\mathrm{m}2$$

From Eqs. (62) and (63), the peak electric field strength $E$_m_ and root-mean-square ${E}_{rms}$ of the incident microwave field is:

72 $${E}_{m}={\left(\frac{2I}{c{\varepsilon }_{0}}\right)}^{1/2}=793.5\mathrm{ V}/\mathrm{m}$$

73 $${E}_{rms}=\sqrt{{E}_{m}^{2}/2}=561.1 \mathrm{V}/\mathrm{m}$$

Similarly, for the 440 W microwave power level, the peak electric field strength ${E}_{m}$ is 1.67 × 10^3^ V/m, and the ${E}_{rms}$ is 1.18 × 10^3^ V/m. The estimated electric field is the power absorbed by the air. However, it is assumed that when the microwave field generation is initiated by the magnetron, the electric field strength within the microwave oven is assumed to be equivalent to the value of the ${E}_{rms}$ as the dimension of the sample (4 cm^3^) is relatively smaller than the cavity size (27,520 cm^3^). The energy absorbed as a function of time calculated based on the ${E}_{rms}$ calculated above is estimated as follows:

74 $$P_{estimated} = 2\pi f\varepsilon_{0} \varepsilon^{\prime \prime } E_{rms}^{2} Vt$$

The strain and temperature measurements as a function of energy absorbed in Joules are presented below, under 100 W and 440 W.

**Figure 33:**
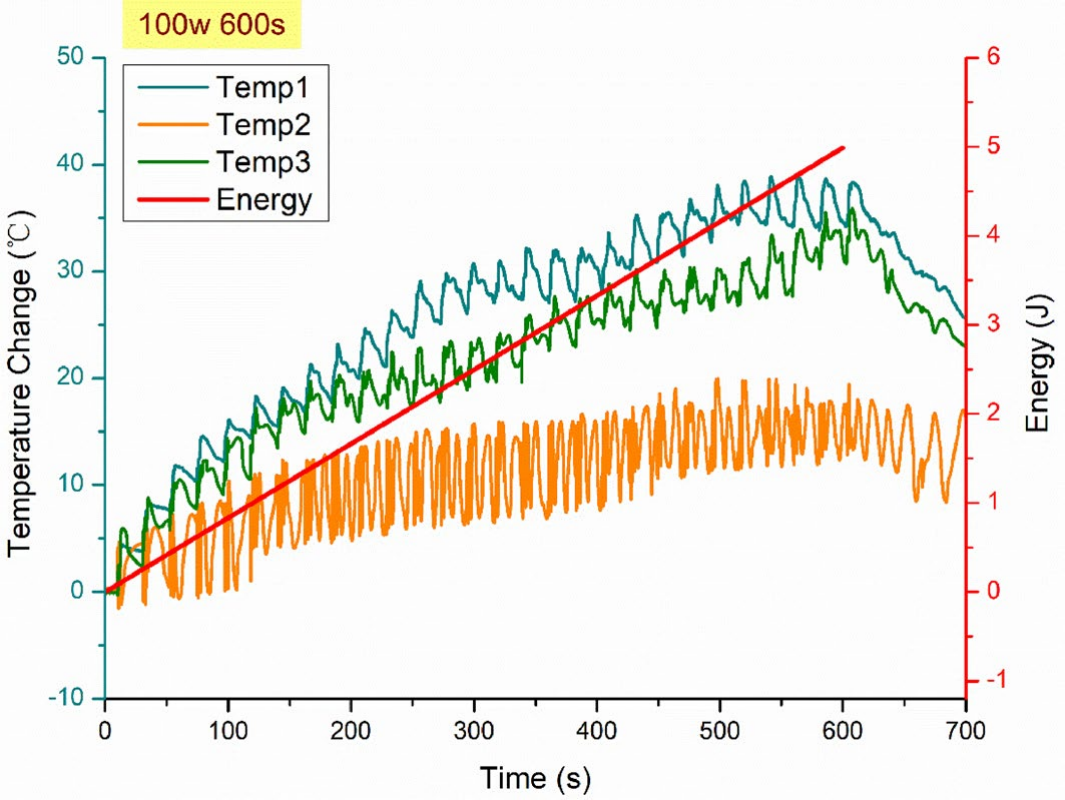
Temperature change measurements (micro-strain) by FBG sensors (left), and energy absorbed (right) versus time (s) under 100 W.

**Figure 34:**
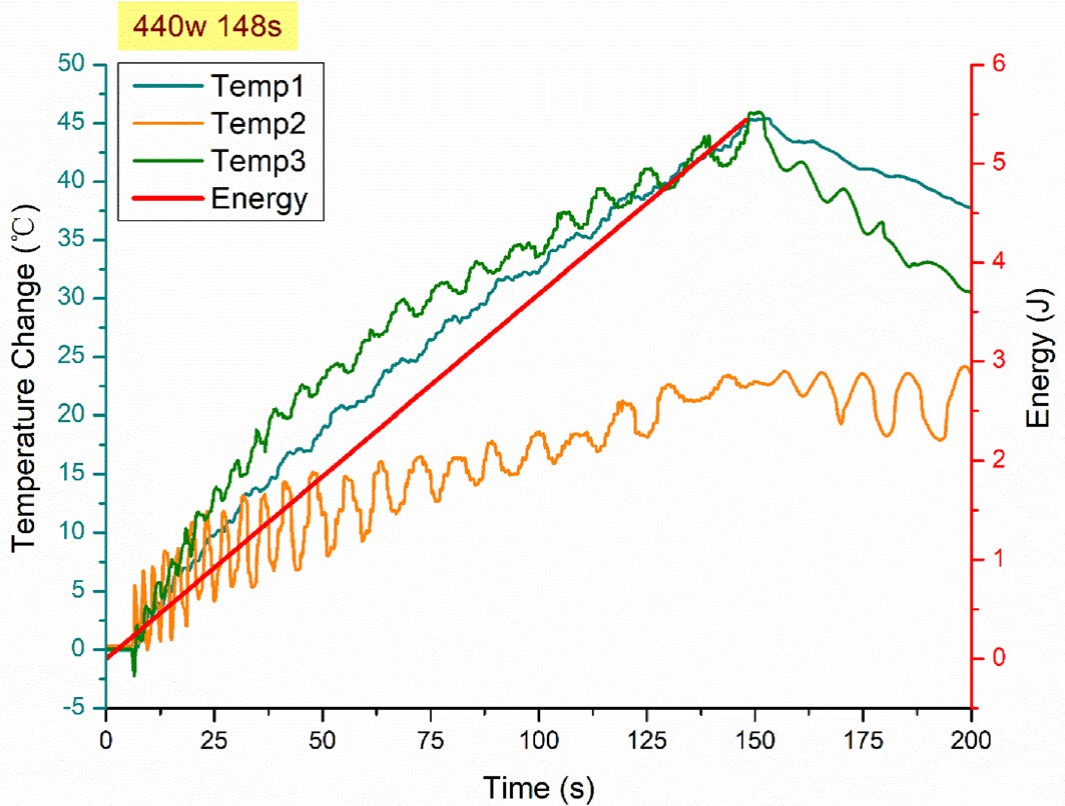
Temperature change measurements (micro-strain) by FBG sensors (left), and energy absorbed (right) versus time (s) under 440 W.

It could be observed from Figs. 33 and 34 that, the energy absorption rate is close to the rate of temperature rise in the FBG sensors. At the beginning of the microwave exposure, the temperature rise exhibits a similar rate compared with the energy absorption rate. As the exposure time increases, the temperature rise becomes significantly slower. The ‘hot spot’ theory due to the non-uniformity of microwave fields is proposed to be the possible explanation of this phenomenon as described formerly.

The internal electric field inside the sample could only be estimated based on the assumption that 100% energy transfers into heat. Therefore, the external electric field of the incident microwave at the surface of the BaTiO_3_-Epoxy sample, ${E}_{rms, external}$ is obtained from Eq. (66), and employed to be the ${E}_{rms}$ to solving the Eq. (22) to estimate the eigenstrain of the BaTiO_3_.

75 $${E}_{rms}=\sqrt{\frac{\rho Ca\Delta T}{3\Delta tc{\varepsilon }_{0}\xi\Gamma }}\approx 12.1\times \sqrt{\frac{\Delta T}{\Delta t}}$$

where calculated $\rho$ by given weight and volume is 1.3 g/cm^3^, $C$ calculated from the DSC spectra is 1.238 J.g^-1^.°C^−1^, $a$ is sample thickness 3 mm, $c$ is the speed of microwave (3 × 10^10^ cm/s), ${\varepsilon }_{0}$ is the permittivity of the free space (8.85 × 10^−14^ F/cm), and $\xi$ and $\Gamma$ is calculated by Eq. (67), Eq. (68), as follows:

76 $$\xi =1-{e}^{-\frac{2a}{{d}_{p}}}\approx 0.007$$

77 $$\Gamma =\frac{4({n}_{1}/{n}_{2})}{{\left[\left({n}_{1}/{n}_{2}\right)+1\right]}^{2}}\approx 0.874$$

where ${d}_{p}$ is calculated to be 850 mm, ${n}_{1}$ is 1 for air and ${n}_{2}=\sqrt{{\varepsilon }^{^{\prime}}}=\sqrt{4.42}$.

At the beginning of the exposure when the microwave incident the sample at $t=10s$ under 100 W, the rate of temperature rise $\frac{\Delta T}{\Delta t}$ is obtained using the linear fit of the temperature change measurements from FBG sensor 1, which is 0.05.

78 $${E}_{rms}=2.71 V/cm$$

The eigenstrain of BaTiO_3_ nanoparticle movements based on the simplified model of Eqs. (21) and (22) at 100 W, ${\varepsilon }_{BaTiO_{3}}$ is:

79 $${\varepsilon }_{100}=\frac{\Delta {l}_{m}}{L}=\frac{{f}_{m}}{{d}^{2}Y}= \frac{\sqrt{2}{P}_{0}{E}_{m}}{{d}^{2}Y}=65\times {10}^{-6}$$

where ${P}_{0}$ is 0.26 C/m^2^, $Y$ is the young’s modulus of BaTiO_3_, which is assumed 13.6 × 10^8^ N/cm^2^^52^.

The domain width $d$ is highly dependent on the grain/crystallite size, therefore, it is estimated to be 4 × 10^−5^ cm with the crystallite size assumed to be equal to the nanoparticle size 200 nm based on the measurements in previous studies^124^, and the strain ${\varepsilon }_{440}$ at $t=7s$ under 440 W with the rate of temperature rise of 0.3, is $144\times {10}^{-6}$. This value could only represent the approximate amplitude of the induced DW displacement under such electric field that impinges the sample surface.

### Post-microwave residual effect in the BaTiO_3_/epoxy nanocomposites

As discussed in Sect. 3, the electric field-induced strain is predominately attributed to the irreversible domain wall movement in multi-domain ferroelectric crystals. Therefore, when the microwave field is removed, a residual stress field is hypothesised, and Raman spectroscopy was employed in this study to probe any possible residual strain effects that originate from microwave field activated BaTiO_3_ nanoparticles. The Raman peaks shift indicated the changes in the atomic spacing of the material, namely the strain information^125^. In the meantime, it was also discovered that the altered stress state surrounding the BaTiO_3_ could introduce a crystal structure change due to the piezoelectricity effect exhibit in ferroelectric materials. Raman data did not indicate a significant difference between the Raman spectra of 15 wt.% nanocomposite samples with and without microwave treatment.

## Conclusions

The quantitative research on microwave field-nanocomposite interaction was conducted to study the ferroelectric materials’ response in high-performance (rigid) epoxy composite that offers reversible microwave activated electro-strains introduced by a second dielectric phase (BaTiO_3_). The data presented a pioneering investigation as no investigations have been reported, thus far, on the micromechanical (extrinsic) strain response of rigidly constrained ferroelectric materials in polymer under external electromagnetic fields. The research has characterised the thermal and dielectric properties of ferroelectric nanoparticles (BaTiO_3_) modified polymer (epoxy) at weight loading up to 15 wt.%, and subsequently investigated the extrinsic strain state induced by the BaTiO_3_ nanoparticles’ dipole displacements onto their surrounding epoxy, however observed at the phenomenological level in the multi-material system. Silane surface functionalisation of the nanoparticles was utilised to uniformly disperse and improve interfacial particle–matrix bonding quality. FBG sensors-based technique was employed, in-situ with the microwave exposure, for real-time monitoring of the strain and temperature response of the nanocomposite subjected to a 2.45 GHz microwave field at controlled exposure power and energy, followed by theoretical constitutive equations underpinning field-material interactions, and the remnant extrinsic strain investigations using Raman spectroscopy. The key remarks on the investigations conducted in this article are as follows:

1. A time-dependent strain field is introduced, proportional to the exposure time/energy, in the multi-domain BaTiO_3_ nanoparticles under the stimulation of a microwave field, linearly under 100 W and non-linearly under 440 W. The strain is theoretically described via development of multi-physics constitutive expressions for the nanoparticle possessing a multi-domain crystallite based on an estimated electric field strength exhibiting at the geometric boundary of the particle.
2. The principle of virtual work was utilised, phenomenologically for the theoretical development, to estimate the strain within the epoxy matrix embedded with BaTiO_3_ subjected to the microwave exposure, able to underpin the in-situ induced micro-straining performance of the BaTiO_3_-epoxy nanocomposite.
3. A sudden drop in micro-strain data by the value of approx. 1000 compressive micro-strains was observed in the BaTiO_3_-epoxy composites at the beginning phase of the exposure under the different exposure powers examined. Such phenomenon has not been reported thus far in the existing literature, however investigated herein using different characterisation and theoretical development at micromechanical level. The drop indicated an instantaneously induced compressive strain achieved within a small fraction of a second, which is recovered afterwards with the increasing exposure energy, followed by a gradual increase during the exposure. No arcing in the microwave was observed, and no damage in any part of the FBG, adhesive, composite and PTFE; The temperature variation due to the exposure was kept well below the ${T}_{g}$, and the DSC investigations presented a fully cured nanocomposite. The theoretical–experimental study of the response led to the fact that such effect is caused by the DW movement response of the BaTiO_3_-epoxy composite under microwave exposure, which is attributed to a mechanical compressive field to its surrounding polymer, with its effect phenomenologically observed by the in-situ FBG technique.
4. The nanocomposite exhibited linear and highly non-linear strain variations under the 100 W and 440 W exposure, respectively. Residual compressive strains were developed post 440 W exposure, offering an analogous response to the mechanical field pre-straining which takes the strain hardening materials (e.g. polycrystalline) beyond its elastic regime. Such strain hardening response in the case of 440 W is attributed to the semi-crystalline phase that BaTiO_3_ had introduced to the nanocomposite.
5. To achieve the full benefit from the electro-strain behaviour of the BaTiO_3_ fillers, uniform dispersion of the nanoparticles in the epoxy matrix was considered to be the primary determinant factor. Large agglomerates were observed in non-functionalised BaTiO_3_-epoxy nanocomposites due to the attraction between the nano-scale particles, and their intrinsic incompatibility with hydro-carbon epoxy matrix. Uniform dispersion of the surface functionalised BaTiO_3_ nanoparticles ensures a uniform application of the domain wall movements triggered stress field to their surrounding epoxy matrix. Furthermore, it also avoids the possible negative attributes from large agglomerations to the mechanical performances, as the aggregation of nanoparticles might act as a weak spot compared with the uniform dispersed nanoparticles. The efficiency of silane functionalisation on the uniform dispersion of BaTiO_3_ nanoparticles was demonstrated.
